# Effectiveness and Safety of Oral Anticoagulants in Older Patients With Atrial Fibrillation: A Systematic Review and Meta-Analysis

**DOI:** 10.3389/fphar.2020.583311

**Published:** 2020-09-09

**Authors:** Maxim Grymonprez, Stephane Steurbaut, Tine L. De Backer, Mirko Petrovic, Lies Lahousse

**Affiliations:** ^1^ Pharmaceutical Care Unit, Department of Bioanalysis, Ghent University, Ghent, Belgium; ^2^ Centre for Pharmaceutical Research, Vrije Universiteit Brussel, Jette, Belgium; ^3^ Department of Cardiology, Ghent University Hospital, Ghent, Belgium; ^4^ Department of Geriatrics, Ghent University Hospital, Ghent, Belgium; ^5^ Department of Epidemiology, Erasmus Medical Center, Rotterdam, Netherlands

**Keywords:** atrial fibrillation, oral anticoagulant, increased age, multimorbidity, polypharmacy, fall, frailty, dementia

## Abstract

**Background and Objective:**

Atrial fibrillation (AF), the most common cardiac arrhythmia, typically increases with age. Oral anticoagulants (OACs) are the cornerstone of treatment to reduce the associated risk for systemic thromboembolism. Four large randomized controlled trials (RCTs) have shown that non-vitamin K antagonist oral anticoagulants (NOACs) are non-inferior to vitamin K antagonists (VKAs) in preventing stroke and systemic embolism, as well as regarding their risk for major bleeding. However, as vulnerable geriatric patients with AF were largely underrepresented in these trials, physicians are faced with the challenge of choosing the right anticoagulant for geriatric patients in real-life clinical practice. In this vulnerable patient group, NOACs tend to be underused or underdosed due to concerns of excessive fall-related intracranial bleeding, cognitive impairment, multiple drug-drug interactions, low body weight or impaired renal function. As life expectancy continues to rise worldwide, the number of geriatric patients substantially increases. Therefore, there is an urgent need for a critical appraisal of the added value of NOACs in geriatric patients with AF at high thromboembolic and bleeding risk.

**Methods and Results:**

This systematic review provides an overview of the literature on the impact of increased age (≥75 years), multimorbidity, polypharmacy, increased falling risk, frailty and dementia on the effectiveness and safety of NOACs as compared to VKAs, after searching the Medline database. Moreover, a meta-analysis on the impact of increased age ≥75 years old was performed after pooling results from 6 *post hoc* analyses of RCTs and 6 longitudinal observational cohort studies, highlighting the superior effectiveness (hazard ratio (HR) 0.83, 95% confidence interval (CI) [0.74–0.94] for stroke/SE; HR 0.77, 95%CI [0.65–0.92] for mortality) and non-inferior safety (HR 0.93, 95%CI [0.86–1.01] for major bleeding; HR 0.58, 95%CI [0.50–0.67] for intracranial bleeding; HR 1.17, 95%CI [0.99–1.38] for gastrointestinal bleeding) of NOACs versus VKAs in older AF patients.

**Conclusion:**

Across geriatric subgroups, apixaban was consistently associated with the most favourable benefit-risk profile and should therefore be preferred in geriatric patients with AF. However, research gaps on the impact of increased falling risk, frailty and baseline dementia were identified, requiring careful consideration while awaiting more results.

## Introduction

As life expectancy continues to rise worldwide, the number of geriatric patients substantially increases (Beard et al., 2016). In older patients ≥75 years old, multimorbidity, polypharmacy, recurring falling incidents, frailty and dementia tend to rise in prevalence and tend to coincide (Jaspers Focks et al., 2016; Piccini et al., 2016; Steffel et al., 2016; Rao et al., 2018; Martinez et al., 2018; Alexander et al., 2019). Although high age, frequently defined in studies as ≥75 years, is not a de facto criterion for a geriatric profile, it has been independently associated with higher risks of systemic thromboembolism, major bleeding, intracranial bleeding and mortality (Wolf et al., 1991; Oldgren et al., 2011; Halvorsen et al., 2014; Kato et al., 2016; Chao et al., 2020; Kirchhof et al., 2020). Moreover, the incidence and prevalence of atrial fibrillation (AF), the most frequent cardiac arrhythmia worldwide, typically increases with age (Heeringa et al., 2006; Lee et al., 2017). Oral anticoagulants (OACs) are crucial to reduce the associated risk of systemic thromboembolism in non-valvular AF (hereby referenced as AF) (Steffel et al., 2018). Four large phase III randomized controlled trials (RCTs) (RE-LY trial for dabigatran (Connolly et al., 2009), ROCKET AF trial for rivaroxaban (Patel et al., 2011), ARISTOTLE trial for apixaban (Granger et al., 2011), ENGAGE AF-TIMI 48 trial for edoxaban (Giugliano et al., 2013)) have shown that non-vitamin K antagonist oral anticoagulants (NOACs) are at least non-inferior for stroke prevention and for the risk of bleeding events as compared to vitamin K antagonists (VKAs) (Connolly et al., 2009; Patel et al., 2011; Granger et al., 2011; Giugliano et al., 2013; Ruff et al., 2014). However, concerns have risen regarding the effectiveness and safety of NOACs in real-life clinical practice in patients with multiple comorbidities and concomitant medication use, especially vulnerable geriatric patients with AF who were largely underrepresented in these trials (Lee et al., 2012). Consequently, NOACs tend to be underused or underdosed in these patients due to concerns of excessive fall-related intracranial bleeding, cognitive impairment with suboptimal therapy adherence, multiple drug-drug interactions (DDIs), low body weight or impaired renal function (Viscogliosi et al., 2017; Oqab et al., 2018; Proietti et al., 2019; Madhavan et al., 2019; Besford et al., 2020; Kapoor et al., 2020; Sanghai et al., 2020). Therefore, there is an urgent need for a critical appraisal of the added value of NOACs in geriatric patients with AF at high thromboembolic and bleeding risk.

This systematic review will provide an overview of the literature on the impact of increased age (≥75 years), multimorbidity, polypharmacy (≥5 drugs) (Masnoon et al., 2017), high falling risk, frailty and dementia on the effectiveness and safety of NOACs versus VKAs in geriatric patients with AF. Moreover, a meta-analysis on the impact of increased age ≥75 years old on NOAC versus VKA effectiveness and safety will be performed. Thereby, this overview will help guide physicians in their OAC choice for vulnerable older patients with AF.

## Methods

A thorough literature search was performed using the Medline database by one reviewer (MG) (see supplemental materials, **eTable 1**). Articles related to oral anticoagulant use for stroke prevention in adult patients with non-valvular AF and increased age (≥75 years), multimorbidity, polypharmacy (≥5 drugs), high falling risk, frailty and baseline dementia were selected. Only studies longitudinally comparing the effectiveness and safety of NOACs (dabigatran, rivaroxaban, apixaban and/or edoxaban) compared to VKAs (warfarin, phenprocoumon and/or acenocoumarol) during a mean/median follow-up of at least 3 months in these patient subgroups were included. Studies regarding OAC use for non-AF indications (e.g. venous thromboembolisms or mechanic heart valves) were excluded if no separate results of patients with AF were provided. Effectiveness and safety outcomes of interest were stroke or systemic embolism (stroke/SE), major bleeding (overall, intracranial and/or gastrointestinal) and all-cause mortality. RCTs (original trial or *post hoc* analyses), longitudinal observational cohort studies and meta-analyses written in English were included for a qualitative synthesis, while reviews, cross-sectional studies, case reports, editorials or conference proceedings were left out of consideration. For a quantitative synthesis (meta-analysis), only *post hoc* analyses of RCTs and longitudinal observational cohort studies regarding the impact of increased age ≥75 years old on NOAC versus VKA effectiveness (stroke/SE, mortality) and safety (major, intracranial and gastrointestinal bleeding) were included. Studies including even older AF patients (e.g. ≥80, 85, or 90 years old) were not included in the meta-analysis, due to concerns of channelling bias (Alcusky et al., 2020) in the introduction years and selective prescribing (of NOACs to more comorbid patients) later on, and more frequent inappropriate NOAC dosing in observational studies (Shinohara et al., 2019; Raposeiras-Roubín et al., 2020) in the oldest AF patients. However, these results were included in an additional subgroup analysis. No restriction of publication date was used.

**Figure 1:**
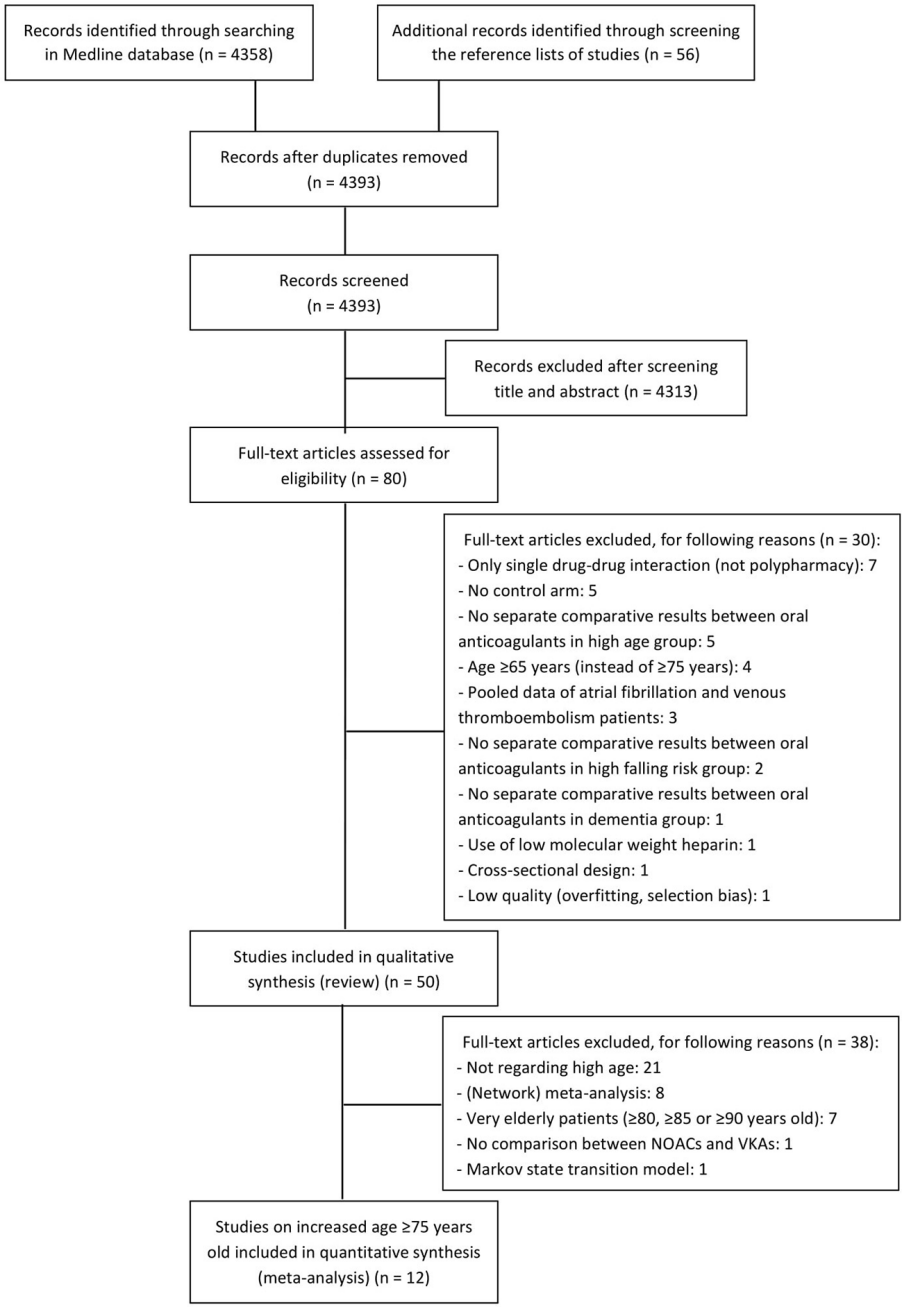
PRISMA flow diagram.

On April 24, 2020, 4358 articles were identified. Additional articles of interest were identified by screening the reference list of studies. After screening title and abstract, 80 articles were selected by one reviewer. After reading the full-text, 50 articles were selected for the qualitative synthesis and 12 for a quantitative synthesis (i.e. 6 *post hoc* analyses of RCTs, 6 observational studies) (**Figure 1**). An overview of the included studies with study design, patient characteristics and outcome measures are displayed in tables (**eTables 2–7**).

For the impact of increased age ≥75 years old, a meta-analysis was performed using a random effects model with inverse-variance weighting with the metafor package in R (R version 3.6.1 with RStudio version 1.2.5001), by pooling results based on the logarithmic adjusted hazard ratios (HRs) and standard error. Data on the study characteristics (design, setting and duration), baseline characteristics of included patients (total number and age), intervention (e.g. NOAC versus VKA) and the abovementioned effectiveness and safety outcomes of interest were extracted from the original publications or supplemental materials. Effect sizes were presented as HR with 95% confidence interval (95%CI) for the outcome of interest of NOAC versus VKA users in forest plots using the forestplot package in R. A two-sided p-value of <0.05 was considered statistically significant. Heterogeneity was tested using the I²-statistic and Cochran’s Q-test, based on a restricted maximum-likelihood estimator. To assess the risk of bias of each study included in the meta-analysis, the quality assessment tool “QUALSYST” from the “Standard Quality Assessment Criteria for Evaluating Primary Research Papers from a Variety of Fields” was used (**eTable 8**) (Kmet et al., 2004). With this tool, 14 items of each quantitative study were scored on the study and outcome levels depending on the degree to which the specific criteria were met or reported (“yes” = 2, “partial” = 1, “no” = 0). Items not applicable to a particular study design were marked “n/a” and were excluded from the calculation of the summary score. A percentage was calculated for each paper by dividing the total sum score obtained across rated items by the total possible score. Studies were included if scoring at least 80% on the quality assessment tool. The risk of publication bias at the outcome level for the studies included in the meta-analysis was assessed through funnel plot asymmetry and Egger’s regression test. This work has been performed according to the Preferred Reporting Items for Systematic Reviews and Meta-Analyses (PRISMA) guidelines (PRISMA checklist included in supplemental materials, **eTable 9**).

## Results

### Increased Age

#### Randomized Studies

Several *post hoc* analyses and meta-analyses of the pivotal phase III RCTs have been performed, illustrating similar stroke/SE and mortality risks with reduced dose NOACs in AF patients ≥75 years old as compared to warfarin, whereas significantly lower stroke/SE and mortality risks with standard dose NOACs were observed (**eTable 2**) (Ruff et al., 2014; Sadlon and Tsakiris, 2016; Kim et al., 2018; Caldeira et al., 2019; Malik et al., 2019). Furthermore, besides a significantly lower intracranial bleeding risk and a similar major bleeding risk for both standard and reduced dose NOACs (Ruff et al., 2014; Sadlon and Tsakiris, 2016; Kim et al., 2018; Caldeira et al., 2019; Malik et al., 2019), a similar to significantly higher gastrointestinal bleeding risk for reduced and standard dose NOACs respectively has been illustrated (Kim et al., 2018; Malik et al., 2019). However, substantial heterogeneity was detected in these meta-analyses for the bleeding risk assessment in older patients (I²-value ranging from 84% (Malik et al., 2019) to 94%) (Kim et al., 2018), potentially attributed to differences in the safety profile of individual NOACs (Sadlon and Tsakiris, 2016; Kim et al., 2018; Caldeira et al., 2019; Malik et al., 2019). Indeed, in the individual *post hoc* analyses of RCTs, an increased bleeding risk for dabigatran and rivaroxaban was observed in older AF patients, as opposed to lower risks for apixaban and edoxaban.

In a subgroup analysis of the RE-LY trial, a significant interaction between age and treatment for major and gastrointestinal bleeding was seen for dabigatran (Eikelboom et al., 2011). In AF patients ≥75 years old, similar major bleeding and significantly higher gastrointestinal bleeding risks were seen for both dabigatran doses (Eikelboom et al., 2011). In AF patients 80–84 years old, significantly higher major bleeding and major extracranial bleeding risks, and a similar intracranial bleeding risk was observed for standard dose dabigatran (150 mg), whereas a significantly lower intracranial bleeding, similar major bleeding and significantly higher extracranial bleeding risk was noted for reduced dose dabigatran (110 mg) as compared to warfarin (Lauw et al., 2017). The point of reversal from lower to higher major bleeding rates along the age spectrum was estimated to be >77 years for dabigatran 150 mg and >80 years for dabigatran 110 mg. For extracranial major bleeding, this reversal point was >74 years and >76 years respectively. Based on these results, an age of 75–80 years was implemented as a criterion to consider dose reduction and ≥80 years of age was implemented as a dose reduction criterion for dabigatran (Boehringer Ingelheim, 2010; Eikelboom et al., 2011). Nonetheless, these results illustrate the potentially worse safety outcomes for dabigatran in older patients, especially regarding the gastrointestinal bleeding risk. Moreover, worse safety outcomes have been observed for rivaroxaban in AF patients ≥75 years old, as a *post hoc* analysis of the ROCKET AF trial documented significantly higher gastrointestinal bleeding risks, whereas similar major bleeding and intracranial bleeding risks for rivaroxaban as compared to warfarin were noted (Halperin et al., 2014). Similarly, in the Japanese J-ROCKET AF trial, rivaroxaban use in older AF patients was associated with a similar major bleeding risk (Hori et al., 2014). On the contrary, apixaban use has been associated with a significantly lower major bleeding, intracranial bleeding and major bleeding risk as compared to warfarin in AF patients ≥75 years old in a subgroup analysis of the ARISTOTLE trial (no report of gastrointestinal bleeding risk) (Halvorsen et al., 2014). Even in an exploratory analysis among AF patients ≥80 years old, superior safety results were observed. Likewise, edoxaban use in AF patients ≥75 years old was associated with a similar (standard dose edoxaban) to significantly lower (reduced dose edoxaban) major bleeding risk and a significantly lower intracranial bleeding risk as compared to warfarin in a *post hoc* analysis of the ENGAGE AF-TIMI 48 trial, although a significantly higher gastrointestinal bleeding risk was observed (Kato et al., 2016). Results were consistent in patients ≥80 and ≥85 years old.

Based on the abovementioned results, network meta-analyses have specifically compared the efficacy and safety of NOACs in AF patients ≥75 years old (Lin et al., 2015; Sadlon and Tsakiris, 2016; Malik et al., 2019; Deng et al., 2020). Despite a similar stroke/SE risk (Lin et al., 2015; Sadlon and Tsakiris, 2016; Malik et al., 2019), these indirect head-to-head comparisons between NOACs have highlighted significantly lower major bleeding risks for apixaban and edoxaban as compared to dabigatran (both doses) and rivaroxaban, except for a similar risk between edoxaban and dabigatran 110 mg (Lin et al., 2015; Sadlon and Tsakiris, 2016; Malik et al., 2019). No significant differences in major bleeding were observed when indirectly comparing apixaban to edoxaban, and dabigatran to rivaroxaban (Lin et al., 2015; Malik et al., 2019). Importantly, rivaroxaban was associated with a significantly higher risk for intracranial bleeding as compared to other NOACs (Lin et al., 2015; Malik et al., 2019). Moreover, a network meta-analysis that estimated the rank probability of OACs in AF patients ≥75 years old, which reflects the hierarchy of drugs on efficacy and safety, showed that apixaban ranked best on both stroke/SE prophylaxis (followed by rivaroxaban, edoxaban, dabigatran 110 mg and warfarin) and major bleeding risk (followed by edoxaban, dabigatran 110 mg, warfarin, and rivaroxaban) (Deng et al., 2020). In another network meta-analysis, although dabigatran 150 mg ranked best on efficacy outcomes followed by apixaban, apixaban also ranked best on safety measures while dabigatran 150 mg the worst (Malik et al., 2019).

In conclusion, these *post hoc* analyses and meta-analyses of RCTs have shown that apixaban is associated with the best efficacy and safety profile of all OACs in older AF patients, followed by edoxaban (Lin et al., 2015; Sadlon and Tsakiris, 2016; Malik et al., 2019; Deng et al., 2020).

#### Observational Studies

As older AF patients included in RCTs may have been relatively less comorbid and more compliant, there are concerns regarding the extrapolation of these results to real-life clinical practice. Moreover, the number of very old patients (≥85 years old) was limited in these RCTs. Therefore, post-surveillance observational studies are equally important in the evaluation of the effectiveness and safety of NOACs in older AF patients. Several have been performed in different age strata, however, mostly without edoxaban data, and have described comparable results as the randomized studies, illustrating the non-inferior to superior effectiveness and safety of NOACs over VKAs, the benefit of OAC continuation over discontinuation and the superior safety profile of apixaban (**eTable 2**).

In terms of effectiveness, NOACs had an equal stroke/SE risk as compared to VKAs in AF patients ≥75, ≥80, ≥85, and ≥90 years old (Avgil-Tsadok et al., 2016; Lai et al., 2018; Giustozzi et al., 2019; Hohmann et al., 2019; Nishida et al., 2019; Mitchell et al., 2019; Russo et al., 2019; Shinohara et al., 2019; Alcusky et al., 2020). Some studies even described a significantly lower stroke/SE (Deitelzweig et al., 2019; Kim et al., 2019) and ischemic stroke risk (Mitchell et al., 2019; Deitelzweig et al., 2019; Chao et al., 2020), as opposed to a higher stroke/transient ischemic attack (TIA) risk in one small Italian study (Poli et al., 2019) and a borderline increased ischemic stroke/TIA risk for apixaban in another study due to off-label underdosing (Alcusky et al., 2020). Mortality rates in NOAC users were similar (Nishida et al., 2019; Mitchell et al., 2019) to even significantly lower (Deitelzweig et al., 2019; Kim et al., 2019; Poli et al., 2019; Russo et al., 2019; Alcusky et al., 2020; Chao et al., 2020) as compared to warfarin. In terms of safety, NOACs were associated with a similar (Giustozzi et al., 2019; Mitchell et al., 2019; Nishida et al., 2019; Poli et al., 2019; Russo et al., 2019; Chao et al., 2020) to lower (Kim et al., 2019; Shinohara et al., 2019; Nishida et al., 2019; Chao et al., 2020; Wong et al., 2020) major bleeding, a similar (Hohmann et al., 2019; Kim et al., 2019) to significantly higher (Mitchell et al., 2019; Wong et al., 2020) gastrointestinal bleeding and a lower (Hohmann et al., 2019; Kim et al., 2019; Mitchell et al., 2019; Chao et al., 2020; Wong et al., 2020) intracranial bleeding risk (except for a similar risk in one study) (Russo et al., 2019) as compared to VKAs in AF patients ≥75, ≥80, ≥85 and ≥90 years old (Shinohara et al., 2019; Hohmann et al., 2019; Nishida et al., 2019; Mitchell et al., 2019; Giustozzi et al., 2019; Russo et al., 2019; Kim et al., 2019; Poli et al., 2019; Chao et al., 2020). Interestingly, in AF patients ≥90 years old, the use of NOACs as compared to no anticoagulation was associated with a significantly lower risk for the composite effectiveness endpoint (stroke/SE, pulmonary embolism and death), and a borderline similar risk for major bleeding and intracranial bleeding (Raposeiras-Roubín et al., 2020). On the contrary, VKAs as compared to no anticoagulation were associated with a similar risk for the composite effectiveness endpoint, but a significantly higher risk for major bleeding and intracranial bleeding (Raposeiras-Roubín et al., 2020). This differential safety profile was also illustrated in a Markov state transition model, demonstrating a lack of net clinical benefit for warfarin as compared to no anticoagulation after the age of 87, whereas only after the age of 92 for apixaban (Shah et al., 2019). In other words, even the oldest AF patients appear to still benefit from NOACs instead of discontinuing anticoagulation.

Moreover, in line with randomized studies, differences in safety outcomes between NOACs were seen. Apixaban was associated with a significantly lower major bleeding and intracranial bleeding risk as compared to VKAs in ≥75 and ≥80 year old AF patients (Deitelzweig et al., 2019; Hohmann et al., 2019; Alcusky et al., 2020; Wong et al., 2020). Importantly, as the ARISTOTLE trial did not provide data on the gastrointestinal bleeding risk of apixaban in older AF patients, observational studies were reassuring, illustrating a similar (Wong et al., 2020). to significantly lower (Deitelzweig et al., 2019; Hohmann et al., 2019) gastro-intestinal bleeding risk of apixaban as compared to VKAs in older AF patients. Dabigatran was associated with a similar (Avgil-Tsadok et al., 2016; Deitelzweig et al., 2019; Alcusky et al., 2020) to significantly lower (Wong et al., 2020) major bleeding risk, a similar (Lai et al., 2018; Deitelzweig et al., 2019; Hohmann et al., 2019; Wong et al., 2020) to a significantly higher (Avgil-Tsadok et al., 2016) gastrointestinal bleeding risk, and a significantly lower (Avgil-Tsadok et al., 2016; Lai et al., 2018; Deitelzweig et al., 2019; Wong et al., 2020) intracranial bleeding risk as compared to warfarin in ≥75 in ≥75, ≥80 and ≥85 year old, ≥80 in ≥75, ≥80 and ≥85 year old, and ≥85-year-old AF patients (Avgil-Tsadok et al., 2016; Lai et al., 2018; Deitelzweig et al., 2019; Hohmann et al., 2019; Alcusky et al., 2020; Wong et al., 2020). On the contrary, rivaroxaban was associated with a similar (Alcusky et al., 2020) to significantly higher (Deitelzweig et al., 2019; Wong et al., 2020) major bleeding risk, a similar (Lai et al., 2018) to significantly higher (Deitelzweig et al., 2019; Hohmann et al., 2019; Wong et al., 2020) gastrointestinal bleeding risk, and a similar (Lai et al., 2018) to significantly lower (Deitelzweig et al., 2019; Wong et al., 2020) intracranial bleeding risk as compared to warfarin in ≥75 in ≤75, ≤80 and ≤85 year old, ≥80 in ≤75, ≤80 and ≤85 year old, and ≥85 in ≤75, ≤80 and ≤85 year old year-old AF patients (Lai et al., 2018; Deitelzweig et al., 2019; Hohmann et al., 2019; Alcusky et al., 2020; Wong et al., 2020). In a head-to-head comparison between NOACs in AF patients ≥80 years, apixaban was associated with a significantly lower risk of stroke/SE, major bleeding, gastrointestinal bleeding and mortality as compared to dabigatran and rivaroxaban, and even a significantly lower risk of intracranial bleeding as compared to rivaroxaban (Deitelzweig et al., 2019).

In conclusion, observational studies have illustrated the non-inferior to superior effectiveness and safety profile of NOACs as compared to VKAs in older AF patients, with most reassuring data on apixaban. Importantly, even in the oldest AF patients ≥90 years old, NOAC use was still beneficial over OAC discontinuation (Raposeiras-Roubín et al., 2020).

#### Meta-Analysis

**Figure 2:**
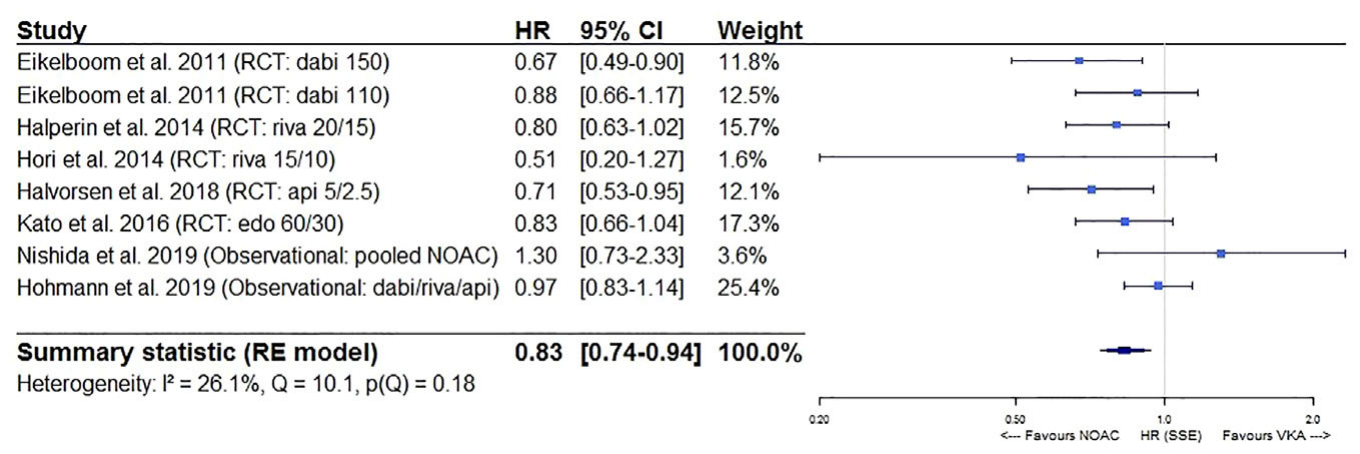
Forest plot of the risk of stroke or systemic embolism of NOACs versus VKAs in elderly atrial fibrillation patients ≥75 years old. Api 5/2.5, apixaban 5 mg (standard dose) and 2.5 mg (reduced dose); CI, confidence interval; Dabi 150, dabigatran 150 mg (standard dose); Dabi 110, dabigatran 110 mg (reduced dose); Edo 60/30, edoxaban 60 mg (standard dose) and 30 mg (reduced dose); HR, hazard ratio; NOAC, non-vitamin K antagonist oral anticoagulant; RCT, randomized controlled trial (*post hoc* analysis); RE model, random effects model; Riva, rivaroxaban; Riva 20/15, rivaroxaban 20 mg (standard dose) and 15 mg (reduced dose); Riva 15/10, rivaroxaban 15 mg (standard dose) and 10 mg (reduced dose); Stroke/SE, stroke/systemic embolism; VKA, vitamin K antagonist.

**Figure 3:**
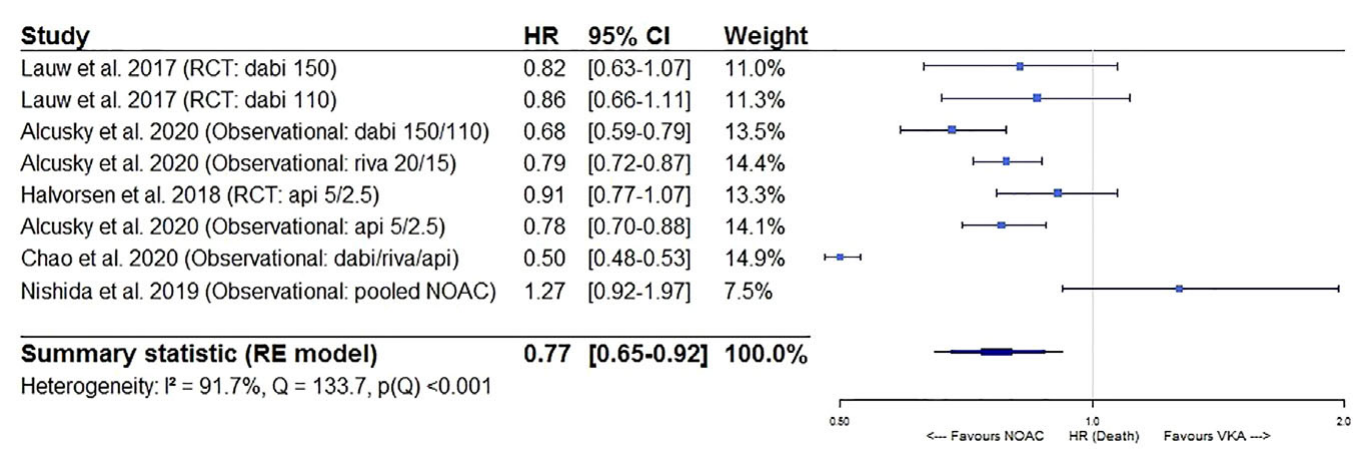
Forest plot of the risk of all-cause mortality of NOACs versus VKAs in elderly atrial fibrillation patients ≥75 years old. Api 5/2.5, apixaban 5 mg (standard dose) and 2.5 mg (reduced dose); CI, confidence interval; Dabi 150, dabigatran 150 mg (standard dose); Dabi 110, dabigatran 110 mg (reduced dose); Death, all-cause mortality; HR, hazard ratio; NOAC, non-vitamin K antagonist oral anticoagulant; RCT, randomized controlled trial (*post hoc* analysis); RE model, random effects model; Riva, rivaroxaban; Riva 20/15, rivaroxaban 20 mg (standard dose) and 15 mg (reduced dose); VKA, vitamin K antagonist.

After pooling the results of 6 *post hoc* analyses of RCTs and 6 observational studies in a meta-analysis, a significantly lower stroke/SE and all-cause mortality risk of NOACs versus VKAs in AF patients ≥75 years old was observed (HR 0.83, 95%CI [0.74-0.94], I² 26.1% for stroke/SE; HR 0.77, 95%CI [0.65-0.92], I² 91.7% for mortality) (**Figures 2** and **3**). The considerable heterogeneity noted for mortality outcomes may be due to heterogeneous mortality results in two observational studies (Nishida et al., 2019; Chao et al., 2020). When performing a sensitivity analysis excluding these two studies, a significantly lower all-cause mortality risk was still present and heterogeneity was low (HR 0.79, 95%CI [0.73-0.86], I² 34.7%, **eFigure 1**).

**Figure 4:**
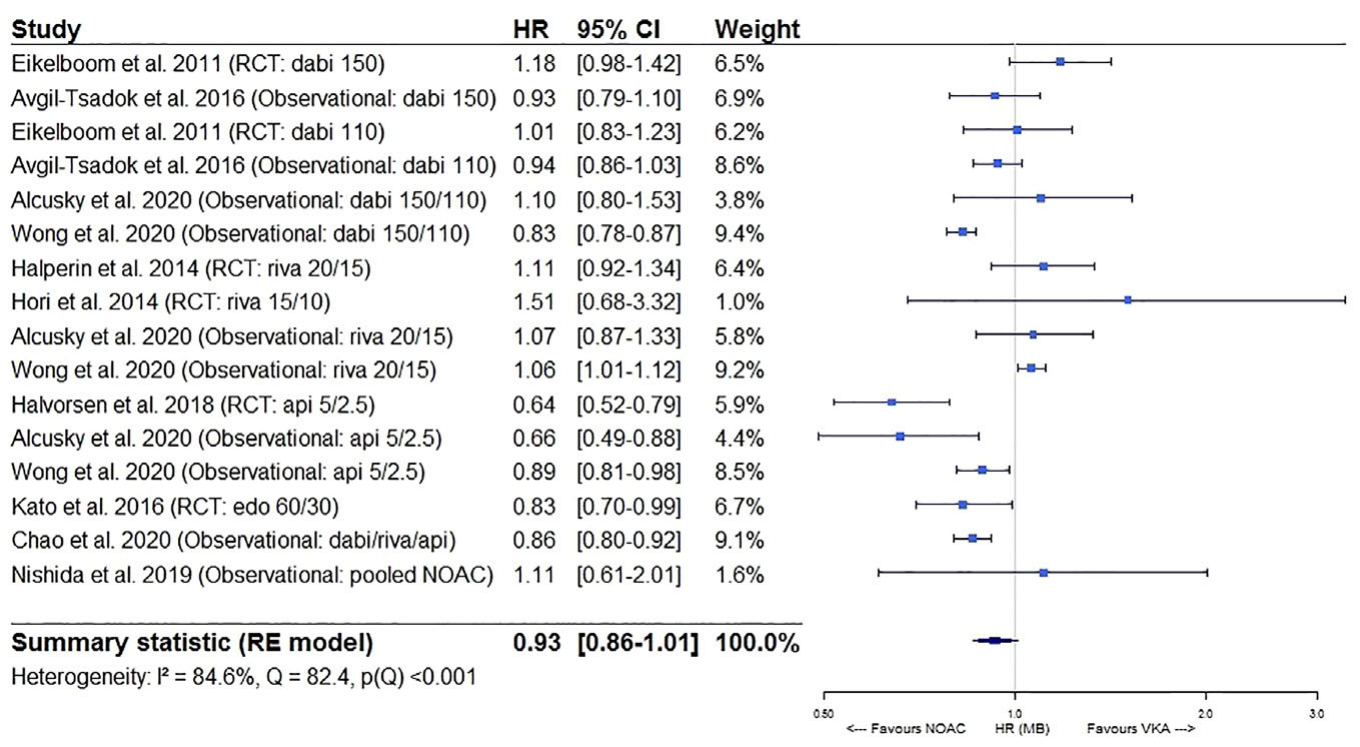
Forest plot of the risk of major bleeding of NOACs versus VKAs in elderly atrial fibrillation patients ≥75 years old. Api 5/2.5, apixaban 5 mg (standard dose) and 2.5 mg (reduced dose); CI, confidence interval; Dabi 150, dabigatran 150 mg (standard dose); Dabi 110, dabigatran 110 mg (reduced dose); Edo 60/30, edoxaban 60 mg (standard dose) and 30 mg (reduced dose); HR, hazard ratio; MB, major bleeding; NOAC, non-vitamin K antagonist oral anticoagulant; RCT, randomized controlled trial (*post hoc* analysis); RE model, random effects model; Riva, rivaroxaban; Riva 20/15, rivaroxaban 20 mg (standard dose) and 15 mg (reduced dose); Riva 15/10, rivaroxaban 15 mg (standard dose) and 10 mg (reduced dose); VKA, vitamin K antagonist.

Major bleeding risks were similar between NOACs and VKAs (HR 0.93, 95%CI [0.86–1.01]), although substantial heterogeneity was present (I² 84.6%), probably due to differential safety profiles of the different types of NOACs used in older AF patients as discussed above (**Figure 4**). Indeed, when performing a sensitivity analysis specifically comparing dabigatran and rivaroxaban to VKAs, major bleeding risks were similar (HR 1.00, 95%CI [0.92–1.09]) with lower but still substantial heterogeneity detected (I² 76.8%) (**eFigure 2A**), although driven by heterogeneous results from observational studies (I² 0.00% for results from RCTs, I² 82.6% for results from observational studies) (**eFigures 2B, C**). When specifically comparing apixaban and edoxaban to VKAs, major bleeding risks were significantly lower (HR 0.77, 95%CI [0.65–0.91], I² 70.9%) (**eFigure 2D**).

**Figure 5:**
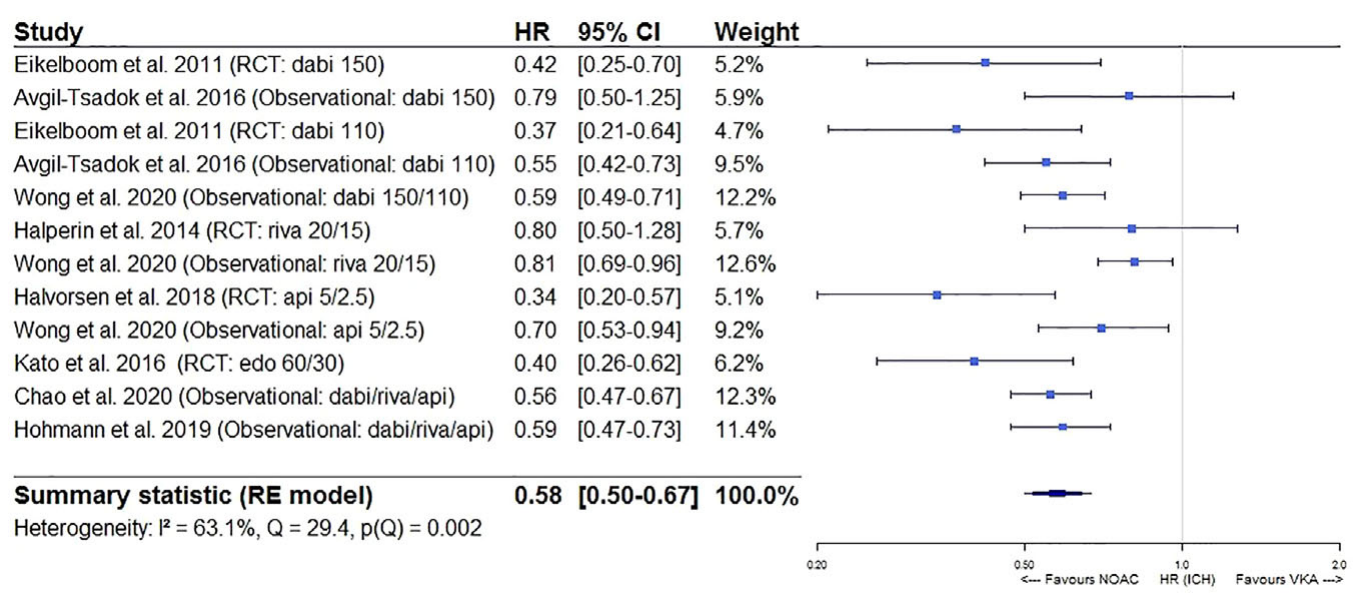
Forest plot of the risk of intracranial bleeding of NOACs versus VKAs in elderly atrial fibrillation patients ≥75 years old. Api 5/2.5, apixaban 5 mg (standard dose) and 2.5 mg (reduced dose); CI, confidence interval; Dabi 150, dabigatran 150 mg (standard dose); Dabi 110, dabigatran 110 mg (reduced dose); Edo 60/30, edoxaban 60 mg (standard dose) and 30 mg (reduced dose); HR, hazard ratio; ICH, intracranial bleeding; NOAC, non-vitamin K antagonist oral anticoagulant; RCT, randomized controlled trial (*post hoc* analysis); RE model, random effects model; Riva, rivaroxaban; Riva 20/15, rivaroxaban 20 mg (standard dose) and 15 mg (reduced dose); VKA, vitamin K antagonist.

**Figure 6:**
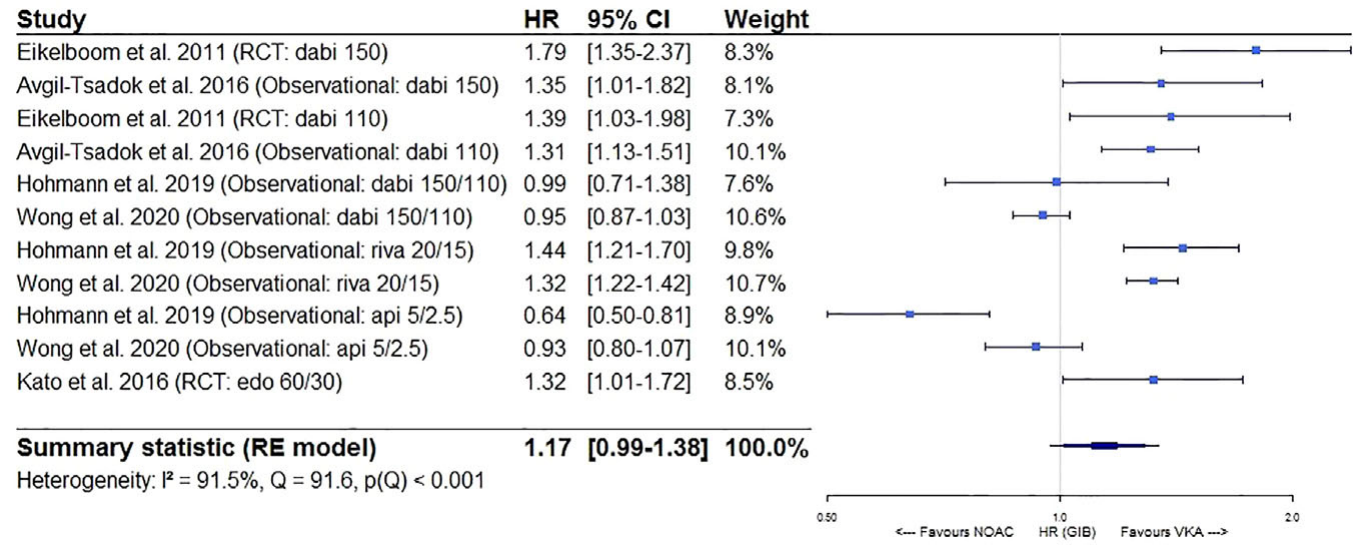
Forest plot of the risk of gastrointestinal bleeding of NOACs versus VKAs in elderly atrial fibrillation patients ≥75 years old. Api 5/2.5, apixaban 5 mg (standard dose) and 2.5 mg (reduced dose); CI, confidence interval; Dabi 150, dabigatran 150 mg (standard dose); Dabi 110, dabigatran 110 mg (reduced dose); Edo 60/30, edoxaban 60 mg (standard dose) and 30 mg (reduced dose); GIB, gastrointestinal bleeding; HR, hazard ratio; NOAC, non-vitamin K antagonist oral anticoagulant; RCT, randomized controlled trial (*post hoc* analysis); RE model, random effects model; Riva, rivaroxaban; Riva 20/15, rivaroxaban 20 mg (standard dose) and 15 mg (reduced dose); VKA, vitamin K antagonist.

Furthermore, a significantly lower intracranial bleeding (HR 0.58, 95%CI [0.50–0.67], I² 63.1%) and a borderline similar gastrointestinal bleeding risk (HR 1.17, 95%CI [0.99–1.38], I² 91.5%) were observed for NOACs as compared to VKAs (**Figures 5** and **6**). In a sensitivity analysis specifically comparing results from dabigatran, rivaroxaban, and edoxaban to VKAs, a significantly higher gastrointestinal bleeding risk (HR 1.28, 95%CI [1.13–1.46], I² 82.6%) was demonstrated (**eFigure 3A**), with substantial heterogeneity driven by dabigatran results (I² 81.9% for dabigatran, I² 0.00% for rivaroxaban, not performed for edoxaban as only one study was available) (**eFigures 3B, C**). However, when comparing apixaban to VKAs, a similar gastrointestinal bleeding risk (HR 0.78, 95%CI [0.54–1.13], I² 86.0%) was observed (**eFigure 3D**).

Moreover, in a subgroup analysis, results from observational studies investigating very old AF patients (≥80, ≥85, or ≥90 years old) were additionally included in the meta-analyses on the effectiveness and safety outcomes of interest. Seven additional observational cohort studies were included (four including AF patients ≥80 years old (Deitelzweig et al., 2019; Kim et al., 2019; Russo et al., 2019; Shinohara et al., 2019), two including AF patients ≥85 years old (Lai et al., 2018; Poli et al., 2019), and one including AF patients ≥90 years old) (Giustozzi et al., 2019). Similar trends were observed, although the major bleeding risk was significantly lower for NOACs as compared to VKAs in AF patients ≥75, ≥80, ≥85, or ≥90 years old (HR 0.92, 95%CI [0.84-0.998], I² 89.1%) (**eFigures 4A–E**).

No publication bias was suspected based on visual inspection of funnel plots (**eFigures 5A–E**), except for mortality outcomes, but this was probably due to considerable heterogeneity in study results. Indeed, after excluding the two most heterogeneous observational studies (Nishida et al., 2019; Chao et al., 2020) in the abovementioned sensitivity analysis, publication bias was no longer suspected (**eFigure 5F**).

In conclusion, NOAC use in AF patients ≥75 years old was associated with a superior effectiveness and a non-inferior safety profile as compared to VKAs in our meta-analysis based on randomized and observational studies, which is in line with the abovementioned RCT-based meta-analyses in older AF patients.

### Multimorbidity

Unfortunately, studies investigating the impact of multimorbidity based on the number of baseline comorbidities, are limited, as only one study has been published so far (**eTable 3**). In this *post hoc* analysis of the ARISTOTLE trial, apixaban use in AF patients with moderate multimorbidity (3–5 comorbidities) was associated with a significantly lower stroke/SE and major bleeding risk, and a similar mortality risk as compared to warfarin, whereas in highly multimorbid AF patients (≥6 comorbidities), all outcome risks were similar (Alexander et al., 2019). More studies investigating the impact of multimorbidity based on the absolute number of baseline comorbidities are needed, although these preliminary results illustrate the preserved efficacy and safety of apixaban, even in patients with high multimorbidity.

A high clinical risk score, such as a high CHADS2, CHA_2_DS_2_-VASc, or HAS-BLED score, can also be used as a proxy to identify patients with multimorbidity, although comorbidities not included in these risk scores are not acknowledged. Several randomized and observational studies have reported outcome rates of NOACs versus VKAs in AF patients with a high clinical risk score, illustrating comparable results as seen in the overall pivotal phase III RCTs (Connolly et al., 2009; Granger et al., 2011; Patel et al., 2011; Giugliano et al., 2013) and studies on increased age, namely the superior efficacy of apixaban and standard dose dabigatran, the (mostly) superior safety of apixaban, non-inferior safety of dabigatran and edoxaban, and non-inferior (in randomized studies) to inferior (in observational studies) safety of rivaroxaban as compared to warfarin (**eTable 3**). Indeed, significantly lower stroke/SE, major bleeding and intracranial bleeding risks, and a similar mortality risk were observed for apixaban-treated patients with a CHADS2 or CHA_2_DS_2_-VASc score of ≥3 as compared to warfarin in a *post hoc* analysis of the ARISTOTLE trial (Granger et al., 2011; Lopes et al., 2012). In the RE-LY trial, dabigatran use in AF patients with a CHADS2 score of ≥3 was associated with a similar (110 mg) to significantly lower (150 mg) stroke/SE risk, a similar major bleeding risk (both doses), a significantly lower intracranial bleeding risk (both doses) and a similar mortality risk (both doses) as compared to warfarin (Connolly et al., 2009; Oldgren et al., 2011). Likewise, non-inferior stroke/SE and major bleeding risks in AF patients with a CHADS2 score of ≥3 were observed in the ROCKET AF trial (Patel et al., 2011) and J-ROCKET AF trial (Hori et al., 2014) for rivaroxaban, and in the ENGAGE AF-TIMI 48 trial (Giugliano et al., 2013) for edoxaban.

In line with these randomized studies, four observational cohort studies also examined the impact of multimorbidity based on high CHA_2_DS_2_-VASc (4–5, ≥6) (Mentias et al., 2018; Hernandez et al., 2018), HAS-BLED (≥4) (Wong et al., 2020), Gagne comorbidity (3–4, ≥5) (Mentias et al., 2018), and/or Charlson Comorbidity Index scores (≥4) (Hohmann et al., 2019). In AF patients with multimorbidity, NOAC use was associated with similar (Mentias et al., 2018; Hohmann et al., 2019) to significantly lower (Hernandez et al., 2018) stroke/SE and mortality risks, and significantly lower (Hohmann et al., 2019; Wong et al., 2020) intracranial bleeding risks as compared to warfarin (Hernandez et al., 2018; Mentias et al., 2018; Hohmann et al., 2019; Wong et al., 2020). On safety outcomes, both apixaban and dabigatran were associated with similar to significantly lower major bleeding and gastrointestinal bleeding risks compared to warfarin, as opposed to similar to significantly higher major bleeding and gastrointestinal bleeding risks for rivaroxaban across studies (Mentias et al., 2018; Hernandez et al., 2018; Hohmann et al., 2019; Wong et al., 2020).

In conclusion, despite at least non-inferior effectiveness outcomes, these observational studies highlight the potential worse safety profile of rivaroxaban as opposed to non-inferior to superior safety profiles of apixaban and dabigatran in AF patients with multimorbidity. These results are in line with the abovementioned results in older AF patients, although safety results of dabigatran appeared to be better in AF patients with multimorbidity due to similar to significantly lower gastrointestinal bleeding risks in observational studies.

### Polypharmacy

Post hoc analyses of two phase III RCTs (the ARISTOTLE (Jaspers Focks et al., 2016) and ROCKET AF trial (Piccini et al., 2016)) have been performed on the impact of polypharmacy, illustrating the at least equal efficacy of apixaban and rivaroxaban, non-inferior to superior safety of apixaban, and non-inferior to inferior safety of rivaroxaban as compared to warfarin (**eTable 4**). Indeed, similar stroke/SE and mortality risks were observed for apixaban- and rivaroxaban- versus warfarin-treated AF patients with polypharmacy (Jaspers Focks et al., 2016; Piccini et al., 2016). Apixaban use in patients with 6–8 and ≥9 drugs was associated with a significantly lower intracranial bleeding and similar gastrointestinal bleeding risk as compared to warfarin (Jaspers Focks et al., 2016), whereas rivaroxaban use in patients with 5–9 and ≥10 drugs was associated with a similar intracranial bleeding risk (no report on gastrointestinal bleeding) (Piccini et al., 2016). Intriguingly, a significant interaction between the number of comedication use and both apixaban and rivaroxaban was present for major bleeding (Jaspers Focks et al., 2016; Piccini et al., 2016). For apixaban, the safety benefit was attenuated in AF patients with the highest number of concomitant medications, as a significantly lower major bleeding risk was observed in patients with 6–8 drugs, whereas an equal risk in patients with ≥9 drugs (Jaspers Focks et al., 2016). For rivaroxaban, a significantly higher major bleeding risk was observed in patients with 5–9 drugs as compared to warfarin, whereas a similar risk in patients with ≥10 drugs (Piccini et al., 2016).

Pooling the results of both RCTs, two meta-analyses illustrated that NOACs were associated with a superior efficacy (significantly lower stroke/SE and all-cause mortality risk) and non-inferior safety (similar major bleeding risk) in AF patients with polypharmacy (≥5 drugs) as compared to warfarin, which is in line with results of our meta-analysis on increased age (Harskamp et al., 2019; Kim et al., 2019).

Furthermore, two observational cohort studies on polypharmacy (≥7 drugs (Hohmann et al., 2019) and ≥5 to ≥10 drugs (Martinez et al., 2019)) illustrated results in line with the abovementioned randomized studies and provided limited reassuring data on dabigatran use in patients with polypharmacy. Similar (Hohmann et al., 2019) to significantly lower (Martinez et al., 2019) stroke/SE and significantly lower (Hohmann et al., 2019) intracranial bleeding risks were observed for NOACs as compared to VKAs in these studies. In one observational study, apixaban was associated with a significantly lower gastrointestinal bleeding and similar other major bleeding risk, dabigatran with a similar gastrointestinal bleeding and lower other major bleeding risk, whereas rivaroxaban with a significantly higher gastrointestinal bleeding and similar other major bleeding risk as compared to phenprocoumon (Hohmann et al., 2019). However, the other observational study, though industry-sponsored, observed similar major bleeding risks with rivaroxaban as compared to warfarin in patients with ≥5 and ≥10 drug used (Martinez et al., 2019).

Overall, results on the impact of polypharmacy were consistent as observed in AF patients with multimorbidity, highlighting the preserved effectiveness of NOACs, the non-inferior to superior safety of apixaban and dabigatran, and the opposing non-inferior to inferior safety of rivaroxaban. However, as both randomized and observational data on apixaban use in patients with polypharmacy was most reassuring, apixaban use also appears to be the first choice in patients with polypharmacy, as seen in older AF patients. Nonetheless, the attenuated safety benefit of apixaban in patients with the highest number of concomitant medications should warrant caution and close monitoring.

### High Falling Risk

A high falling risk or recent fall does not automatically contraindicate OAC use. In a Markov decision analytic model using data on stroke and major bleeding rates in both non-anticoagulated and VKA-treated AF patients ≥65 years old with or without falls, the role for continuing instead of omitting OACs was examined (Man-Son-Hing et al., 1999). Weighing the increased risk for fall-related intracranial haemorrhage against the substantial reduction in ischemic stroke risk among warfarin-treated AF patients as compared to non-anticoagulated patients, a person would have to fall about 295 times in 1 year for warfarin not to be the preferred therapy (Man-Son-Hing et al., 1999). In other words, AF patients at high risk of falling still appear to benefit from anticoagulation despite the associated risk for intracranial haemorrhage. Therefore, it is of importance to evaluate potential differences in outcomes between individual OACs, especially regarding intracranial haemorrhage as the most feared fall-related outcome. However, only two secondary analyses of phase III RCTs studies specifically assessed the impact of high falling risk on NOAC efficacy and safety, namely the ARISTOTLE (Rao et al., 2018) and ENGAGE AF-TIMI 48 trial (Steffel et al., 2016), though these were largely underpowered (**eTable 5**). In apixaban-treated AF patients with ≥1 prior fall in the last year, the risk of intracranial bleeding was significantly lower as compared to warfarin, whereas the risks of stroke/SE, major bleeding and mortality were similar (Rao et al., 2018). Likewise, a significantly lower intracranial bleeding risk, and similar stroke/SE, major bleeding, gastrointestinal bleeding and mortality risks were observed for edoxaban users at high risk of falling as compared to warfarin (Steffel et al., 2016). Besides lack of subgroup analyses of the RE-LY (Connolly et al., 2009) and ROCKET AF trial (Patel et al., 2011), to the best of our knowledge, no large observational studies have been performed so far specifically comparing the effectiveness and safety of individual NOACs in AF patients at high falling risk. This emphasizes an urgent need for more research on the topic to help guide physicians in their OAC choice for AF patients at high falling risk.

While awaiting more results, the preserved efficacy and safety outcomes of apixaban and edoxaban may warrant their use in AF patients prone to fall, especially because of the significantly lower intracranial bleeding risk.

### Frailty

Unfortunately, as the four pivotal phase III RCTs did not specifically include or investigate frail AF patients, especially since patients with an estimated life expectancy of <1–2 years or less than the expected trial duration were excluded (Connolly et al., 2009; Lopes et al., 2010; Ruff et al., 2010; Granger et al., 2011; Patel et al., 2011; Giugliano et al., 2013), randomized data is lacking on the impact of frailty on the efficacy and safety of NOACs in AF. Luckily, limited yet useful observational data is emerging on this clinically relevant topic, highlighting comparable results as seen in studies on increased age, namely the similar effectiveness of all NOACs and the most favourable safety profile of apixaban in contrast to the least favourable profile of rivaroxaban (**eTable 6**).

Indeed, in a retrospective cohort study including frail AF patients using the Johns Hopkins Claims-based Frailty Indicator (Segal et al., 2017), NOAC use was associated with a similar stroke/SE and gastrointestinal bleeding risk, and a significantly lower intracranial and other major bleeding risk as compared to phenprocoumon (Hohmann et al., 2019). Importantly, as seen in studies investigating older AF patients, differential safety outcomes between individual NOACs were noted in frail patients. Apixaban was associated with a significantly lower gastro-intestinal bleeding risk, dabigatran with a similar risk, whereas rivaroxaban with a significantly higher risk as compared to phenprocoumon. Moreover, another retrospective cohort study identified frail AF patients using the same Johns Hopkins Claims-based Frailty Indicator (Segal et al., 2017), and observed similar stroke/SE risk for NOACs as compared to warfarin (Martinez et al., 2018). Apixaban was associated with a significantly lower major bleeding but similar intracranial bleeding risk (though the number of events was very low), whereas dabigatran and rivaroxaban with a similar major bleeding but significantly lower intracranial bleeding risk. Additionally, apixaban and dabigatran were associated with a similar gastrointestinal bleeding risk, but rivaroxaban with a higher risk.

In conclusion, although evidence is limited, these studies illustrate that the effectiveness and safety of NOACs appear to be consistent in frail patients, as observed in older AF patients, with apixaban having the most favourable benefit-risk profile. Nonetheless, more studies are needed on the role of individual NOACs in frail AF patients, especially of edoxaban.

### Dementia

Data on the effectiveness and safety of OACs, especially NOACs, in AF patients with dementia are limited. Unfortunately, phase III RCTs did not include AF patients with dementia due to inability to comply with study-related procedures or to give an informed consent, so no randomized data in this population is available (Connolly et al., 2009; Lopes et al., 2010; Ruff et al., 2010; Granger et al., 2011; Patel et al., 2011; Giugliano et al., 2013; Fanning et al., 2020). However, some observational studies have provided exploratory data on this topic, illustrating the benefit of OAC continuation over discontinuation, as seen in the oldest AF patients ≥90 years old (**eTable 7**). Indeed, warfarin-treated AF patients with dementia in the Swedish Dementia Registry and Veterans Affairs database had significantly lower thromboembolic and mortality risks as compared to non-anticoagulated AF patients with dementia, without significantly increasing major bleeding or non-traumatic intracranial bleeding risks (Orkaby et al., 2017; Subic et al., 2018).

Regarding the comparative effectiveness and safety of NOACs versus VKAs, only one retrospective cohort study provided some preliminary data, illustrating similar stroke/SE and other major bleeding risks, a significantly lower intracranial bleeding risk, and significantly higher gastrointestinal bleeding and mortality risks for NOACs versus warfarin in AF patients with dementia (Fanning et al., 2020). However, analyses were not time-dependent, and results may have been influenced by selective prescribing and pooling of NOAC data, necessitating cautious interpretation of these results.

In conclusion, these limited results are comparable to those observed in AF patients ≥90 years old, namely a potential beneficial role for OAC continuation in AF patients with dementia instead of stopping the OAC (Orkaby et al., 2017; Subic et al., 2018). In other words, dementia in itself should not be viewed as a general contraindication for OACs. However, the severity of dementia should also be assessed when evaluating the necessity for OAC continuation. Moreover, it is still unclear what type of OAC should be preferred in these patients as strong evidence is lacking. This highlights the urgent need for more studies investigating the benefit-risk profile of NOACs in AF patients with cognitive impairment and dementia.

## Discussion

### General Trends

The use of OACs in vulnerable geriatric AF patients is a matter of concern for physicians, faced with the challenge of outweighing the benefits of stroke reduction against the risk of bleeding. Vulnerable older AF patients are frequently characterized by multimorbidity, polypharmacy, increased falling risk, frailty and dementia (Jaspers Focks et al., 2016; Piccini et al., 2016; Steffel et al., 2016; Martinez et al., 2018; Rao et al., 2018; Alexander et al., 2019). Consequently, OACs tend to be inappropriately underdosed or discontinued in these patients subgroups (Viscogliosi et al., 2017; Oqab et al., 2018; Madhavan et al., 2019; Proietti et al., 2019; Besford et al., 2020; Kapoor et al., 2020; Sanghai et al., 2020). However, even in AF patients ≥90 years old (Raposeiras-Roubín et al., 2020), at high risk of falling (Man-Son-Hing et al., 1999) or with dementia (Orkaby et al., 2017; Subic et al., 2018), OAC continuation was still beneficial compared to omitting the OAC. Therefore, very high age, recent fall or cognitive impairment should not be considered as strict contraindications for OAC use, provided that an individual benefit-risk assessment is performed.

Even though the pivotal phase III RCTs were not designed and powered to investigate OAC use in geriatric patients, the available randomized evidence and also post-surveillance observational studies suggest that the effectiveness and safety of NOAC as compared to warfarin remain consistent, with apixaban exhibiting the most favourable benefit-risk profile of all OACs across patient subgroups (see **Table 1** for general overview of results). Our meta-analysis including results of 6 *post hoc* analyses of RCTs and 6 observational studies, highlighted superior results on stroke/SE, mortality and intracranial bleeding risks, whereas non-inferior results on major bleeding and gastrointestinal bleeding risks for NOACs as compared to VKAs in AF patients ≥75 years old. Even after additionally including seven observational studies investigating patients ≥80, ≥85, or ≥90 years old, consistent results were demonstrated, though the major bleeding risk was significantly lower for NOACs as compared to VKAs. However, safety differences between individual NOACs were identified, as increasing age above 75 years significantly interacted with the safety of dabigatran and rivaroxaban, illustrating non-inferior to inferior safety results in older AF patients, especially due to a higher gastrointestinal bleeding risk of both NOACs and a similar intracranial bleeding risk of rivaroxaban as compared to warfarin (Eikelboom et al., 2011; Halperin et al., 2014; Lin et al., 2015; Avgil-Tsadok et al., 2016; Lai et al., 2018; Deitelzweig et al., 2019; Hohmann et al., 2019; Wong et al., 2020). On the contrary, the superior safety profile of apixaban was preserved in older AF patients, with a significantly lower major, intracranial and gastrointestinal bleeding risk as compared to warfarin (Halvorsen et al., 2014; Lin et al., 2015; Deitelzweig et al., 2019; Hohmann et al., 2019; Alcusky et al., 2020; Wong et al., 2020). Likewise, edoxaban was associated with a similar (standard dose) to significantly lower (reduced dose) major bleeding risk and a lower intracranial bleeding risk as compared to warfarin, although higher gastrointestinal bleedings risks were also noted (Kato et al., 2016).

**Table 1:** The effectiveness and safety of each NOAC as compared to vitamin K antagonists in atrial fibrillation patients at increased age (≥75 years old), multimorbidity, polypharmacy, high falling risk, frailty, and baseline dementia.

		**DABIGATRAN**		**RIVAROXABAN**		**APIXABAN**		**EDOXABAN**
**≥75 YEARS OLD**		**150 mg**	**110 mg**					
**Stroke/systemic embolism (SE)**	*RCT*	**↘**	**=**	**=**		**↘**		**=**
	*Obs.*	**= to ↘**		**= to ↘**		**= to ↘**		NR
**Major bleeding**	*RCT*	**=**	**=**	**=**		**↘**		**↘**
	*Obs.*	**= to ↘**		**= to ↗**		**↘**		NR
**Intracranial hemorrhage (ICH)**	*RCT*	**↘**	**↘**	**=**		**↘**		**↘**
	*Obs.*	**↘**		**= to ↘**		**↘**		NR
**Gastrointestinal bleeding (GIB)**	*RCT*	**↗**	**↗**	**↗**		NR		**↗**
	*Obs.*	**= to ↗**		**= to ↗**		**= to ↘**		NR
**Mortality**	*RCT*	**=**	**=**	NR		NR		NR
	*Obs.*	**↘**		**= to ↘**		**↘**		NR
**MULTIMORBIDITY^‡^**		**150 mg**	**110 mg**			**3-5^†^**	**≥6^†^**	
**Stroke/SE**	*RCT*	**↘**	**=**	**=**		**↘**	**=**	**=**
						**↘^‡^**		
	*Obs.*	**= to ↘**		**= to ↘**		**= to ↘**		NR
**Major bleeding**	*RCT*	**=**	**=**	**=**		**↘**	**=**	**=**
						**↘^‡^**		
	*Obs.*	**=**		**= to ↗**		**=**		NR
**ICH**	*RCT*	**↘**	**↘**	NR		NR		NR
	*Obs.*	**= to ↘**		**= to ↘**		**= to ↘**		NR
**GIB**	*RCT*	NR	NR	NR		NR		NR
	*Obs.*	**= to ↘**		**↗**		**= to ↘**		NR
**Mortality**	*RCT*	**=**	**=**	NR		**=**	**=**	NR
	*Obs.*	**↘**		**= to ↘**		**↘**		NR
**POLYPHARMACY**				**≥5 drugs**	** ≥10 drugs**	** >5 drugs**	** ≥9 drugs**	
**Stroke/SE**	*RCT*	NR		**=**		**=**	**=**	NR
	*Obs.*	NR		**↘**	**=**	NR		NR
**Major bleeding**	*RCT*	NR		**↗**	**=**	**↘**	**=**	NR
	*Obs.*	NR		**=**	**=**	NR		NR
**ICH**	*RCT*	NR		**=**		**↘**	**↘**	NR
	*Obs.*	NR		NR		NR		NR
**GIB**	*RCT*	NR		NR	NR	**=**	**=**	NR
	*Obs.*	**=**		**↗**		**↘**		NR
**Mortality**	*RCT*	NR		**=**		**=**	**=**	NR
	*Obs.*	NR		NR		NR		NR
**HIGH FALLING RISK**								
**Stroke/SE**	*RCT*	NR		NR		**=**		**=**
**Major bleeding**	*RCT*	NR		NR		**=**		**=**
**ICH**	*RCT*	NR		NR		**↘**		**↘**
**GIB**	*RCT*	NR		NR		NR		**=**
**Mortality**	*RCT*	NR		NR		**=**		**=**
**FRAILTY**								
**Stroke/SE**	*Obs.*	**=**		**=**		**=**		NR
**Major bleeding**	*Obs.*	**=**		**=**		**↘**		NR
**ICH**	*Obs.*	**↘**		**↘**		**= to ↘**		NR
**GIB**	*Obs.*	**=**		**↗**		**= to ↘**		NR
**Mortality**	*Obs.*	NR		NR		NR		NR
**DEMENTIA^i^**		**NOACs^i^**						
**Stroke/SE**	*Obs.*	**=**						
**Major bleeding**	*Obs.*	**=**						
**ICH**	*Obs.*	**↘**						
**GIB**	*Obs.*	**↗**						
**Mortality**	*Obs.*	**↗**						

= (yellow): non-inferior results (similar risk) when comparing NOAC to VKAs; ↘ (green): superior results (significantly lower risk) when comparing NOAC to VKAs; ↗ (red): inferior results (significantly higher risk) when comparing NOAC to VKAs; = **to** ↘ (yellow-green): non-inferior to superior results, varying across studies; = **to** ↗ (yellow-red): non-inferior to inferior results, varying across studies.

^†^number of baseline comorbidities; ^‡^high clinical risk score (e.g. CHA_2_DS_2_-VASc score ≥3); ^i^careful interpretation of results necessary, as only one observational study provided preliminary (pooled) data.

GIB, gastrointestinal bleeding; ICH, intracranial bleeding; NR, not reported; Obs., longitudinal observational cohort study; RCT, (post hoc analysis of) randomized clinical trial; Stroke/SE, stroke/systemic embolism.

Similarly, in AF patients with multimorbidity or polypharmacy, apixaban (Granger et al., 2011; Jaspers Focks et al., 2016; Alexander et al., 2019; Harskamp et al., 2019; Hohmann et al., 2019) was associated with the most favourable effectiveness and safety profile of all NOACs, followed by edoxaban (Giugliano et al., 2013), dabigatran (Connolly et al., 2009; Oldgren et al., 2011; Hernandez et al., 2018; Mentias et al., 2018; Hohmann et al., 2019; Wong et al., 2020), and rivaroxaban (Piccini et al., 2016; Hernandez et al., 2018; Mentias et al., 2018; Harskamp et al., 2019; Hohmann et al., 2019; Martinez et al., 2019; Wong et al., 2020).

In AF patients at high risk of falling, with frailty or dementia, considerably less evidence was available, mostly due to exclusion of these subjects in RCTs, which complicates recommendations for clinical practice. Therefore, more studies are necessary in these patient subgroups. Notwithstanding, apixaban’s preferential benefit-risk profile was maintained in patients prone to fall and with frailty, illustrating a similar effectiveness and non-inferior to superior safety as compared to warfarin (Rao et al., 2018; Hohmann et al., 2019). The preserved significantly lower intracranial bleeding risk is of particular importance in high-risk fallers (Rao et al., 2018). Furthermore, dabigatran in frail patients (Hohmann et al., 2019) and edoxaban in patients prone to fall (Steffel et al., 2016) illustrated similar benefit-risk profiles as compared to warfarin, whereas rivaroxaban showed a non-inferior to inferior safety profile in frail patients (Martinez et al., 2018; Hohmann et al., 2019). As only one study examined the effectiveness and safety of NOACs as compared to warfarin in AF patients with dementia, illustrating a similar stroke/SE and major bleeding risk, as opposed to a higher gastrointestinal bleeding and mortality risk, there is an urgent need for more research on the effectiveness and safety of individual NOACs in dement AF patients (Fanning et al., 2020).

### Pathophysiological Mechanisms

Several mechanisms for differential safety results of individual NOACs in older AF patients have been proposed. As the decline in renal function gradually progresses with age and the metabolism of dabigatran is the most dependent on renal clearance of all NOACs (80% renal clearance as opposed to only 27% for apixaban) (Steffel et al., 2018), the subsequent higher plasma concentrations of fixed-dose dabigatran may partially explain the increased bleeding risk in older patients (Eikelboom et al., 2011; Lauw et al., 2017). Moreover, as the bioavailability of dabigatran after oral ingestion is the lowest of all NOACs (only 3–7%) (Steffel et al., 2018), intra-intestinal metabolism of the prodrug dabigatran etexilate to the active drug during transit could lead to gradually higher concentrations and local bleeding of the gastrointestinal tract by direct drug exposure at bleeding sensitive foci such as diverticulosis, angiodysplasia and colorectal polyposis (Eikelboom et al., 2011). Since warfarin has a high bioavailability and its anticoagulant mechanism of action depends on hepatic enzymes (vitamin K-dependent γ-carboxylation of coagulation factors II, VII, IX, and X) resulting in less direct drug exposure at intra-intestinal bleeding sensitive foci, this could explain the higher gastrointestinal bleeding risk of dabigatran at higher age as compared to warfarin (Eikelboom et al., 2011). Although rivaroxaban has a very high bioavailability (80%–100% if taken together with food), intestinal clearance through P-glycoprotein (P-gp)-dependent biliary and intestinal excretion is substantial, as rivaroxaban’s clearance is 65% non-renal, 47% of which through intestinal excretion (Steffel et al., 2018). This may lead to high intra-intestinal concentrations of rivaroxaban, locally affecting diseased mucosa and resulting in higher gastrointestinal bleeding risks in older patients as compared to warfarin (Eikelboom et al., 2011). Similarly, the higher gastrointestinal bleeding risk of edoxaban in older patients (Kato et al., 2016) may be due to its 62% bioavailability and 46% intestinal clearance (Steffel et al., 2018). However, as the bioavailability of apixaban is also 50% and the intestinal clearance is similar (48%) (Steffel et al., 2018), this pathophysiological mechanism cannot explain why the gastrointestinal bleeding risk is less pronounced in apixaban. Other age-related pharmacokinetic and -dynamic changes may also play a role, such as the decreased hepatic function with reduced drug clearance (relevant for apixaban and rivaroxaban, being +/- 18% and 25% respectively hepatically metabolized (Steffel et al., 2018)), changes in plasma protein binding due to decreasing albumin levels (most important for rivaroxaban and apixaban, being 95% and 87% plasma protein bound respectively (Steffel et al., 2018)) and the prolonged elimination half-life in older patients (11–13 h for rivaroxaban versus 5–9 h in younger patients) (Grandison and Boudinot, 2000; McLean and Le Couteur, 2004; Mueck et al., 2011; Steffel et al., 2018).

Potential mechanisms on the reduced risk for intracranial haemorrhage in NOACs as compared to VKAs have also been suggested in previous literature. As the elimination half-life of NOACs is approximately 12 h, which is significantly shorter than that of VKAs, early discontinuation in case of head trauma or spontaneous bleeding might limit development and progression of intracranial bleeding (Rao et al., 2018; Steffel et al., 2018). Moreover, as NOACs only target factor IIa (dabigatran) or Xa (rivaroxaban, apixaban and edoxaban), whereas VKAs target factor II, VII, IX, and X, it has been proposed that the lack of impact on factor VII by NOACs may help to decrease trauma-related bleeding, especially intracranial haemorrhage (Eikelboom et al., 2011; Rao et al., 2018). Factor VII is an important coagulation factor of the extracellular pathway, initiating clot formulation together with tissue factor (Eikelboom et al., 2011; Rao et al., 2018). Tissue factor is found in high concentrations in the brain, where it may provide supplemental haemostatic protection together with factor VII in case of trauma (Mackman, 2009; Eikelboom et al., 2011). Indeed, in an exploratory case series analysis in factor VII deficient AF patients, severe bleeding risk was increased in warfarin-treated patients, whereas no haemorrhagic events occurred in dabigatran-treated patients, providing preliminary data on the importance of factor VII in major bleeding events (Arletti et al., 2019). However, larger studies are needed to confirm these findings.

The risk of stroke in older AF patients varies across studies, documenting similar to significantly lower stroke/SE risks for NOACs as compared to warfarin. This may be due to the VKA-associated increase in vascular calcification (Weijs et al., 2011; Deng et al., 2020; Millenaar et al., 2020). However, it should be mentioned that not all stroke events in AF patients are necessarily cardio-embolic in origin, which may affect stroke incidence rates of individual OACs in different studies by chance (Paciaroni et al., 2019). For example, in the RENo study examining NOAC-treated AF patients with an acute ischemic stroke, about 30% of patients had a stroke due to causes other than cardio-embolism (e.g. small vessel disease) (Paciaroni et al., 2019).

Another frequently proposed mechanism, increasing the risk for adverse outcomes in older AF patients, are DDIs. The risk of DDIs increases with the number of comorbidities and comedication use (Jaspers Focks et al., 2016; Piccini et al., 2016; Alexander et al., 2019; Harskamp et al., 2019). VKAs have multiple common drug-drug and drug-food interactions, requiring frequent dose adjustments due to the narrow therapeutic window (Kirchhof et al., 2016; Piccini et al., 2016; Steffel et al., 2018). NOACs have less DDIs, but these should not be neglected (Steffel et al., 2018). Two types can be identified: pharmacokinetic and pharmacodynamic DDIs. For NOACs, two major pharmacokinetic interaction mechanisms are present. First, all NOACs are a substrate of the P-gp efflux transporter, which is mostly present in the gastrointestinal lumen, resulting in gastrointestinal excretion of NOACs after absorption in the gut (Leslie et al., 2005; Steffel et al., 2018; Kim et al., 2019; Washam et al., 2019). Its presence in the liver contributes to hepatobiliary drug excretion, while P-gp transporters located in proximal tubules play a role in the active renal clearance of NOACs (Leslie et al., 2005; Gnoth et al., 2011; Steffel et al., 2018; Kim et al., 2019). Moreover, as P-gp is also expressed in capillary endothelial cells making up the blood-brain barrier to prevent passage of drugs into the brain, P-gp inhibition might slightly increase NOAC concentrations in the brain and potentially decrease the beneficial safety of NOACs on intracranial bleeding risks (Leslie et al., 2005; Gnoth et al., 2011; Kim et al., 2019). Second, apixaban and rivaroxaban are partially dependent on hepatic clearance, mostly mediated through the cytochrome P450 3A4 isoenzyme (CYP3A4) (Steffel et al., 2018; Washam et al., 2019). On the contrary, CYP3A4-mediated hepatic metabolism is not involved in the clearance of dabigatran and only minimally (<4%) in edoxaban (Steffel et al., 2018). Therefore, CYP3A4-mediated DDIs do not significantly affect dabigatran and edoxaban plasma concentrations. P-gp and/or CYP3A4 inhibitors (e.g. amiodarone, dronedarone, verapamil…) can increase NOAC plasma concentration due to a decreased gastrointestinal excretion and/or hepatic metabolism respectively, resulting in an increased bleeding risk (Piccini et al., 2016; Steffel et al., 2018; Kim et al., 2019; Washam et al., 2019). Similarly, P-gp and/or CYP3A4 inducers may decrease plasma concentrations, subsequently increasing thromboembolic risks (Steffel et al., 2018; Washam et al., 2019). It should be noted that in all phase III RCTs, the use of strong CYP3A4 and/or P-gp inhibitors and inducers was prohibited, limiting the generalizability of the results to real-life clinical practice (Connolly et al., 2009; Lopes et al., 2010; Ruff et al., 2010; Granger et al., 2011; Patel et al., 2011; Giugliano et al., 2013; Jaspers Focks et al., 2016). Common pharmacodynamically interacting drugs are antiplatelets, NSAIDs, selective serotonin reuptake inhibitors and corticosteroids, which may increase the risk of bleeding (Steffel et al., 2018). Several studies have been published on the impact of (strong) individual DDIs on NOAC effectiveness and safety. However, the potential synergistic impact of multiple weak-moderate DDIs in one patient may also influence outcomes and should not be neglected, especially not in patients with polypharmacy.

Nevertheless, the differential safety profiles of NOACs in older AF patients cannot be fully explained by these mechanisms, so other unidentified age-dependent pathophysiological mechanisms may contribute as well.

### Strengths and Limitations of Available Literature

The included RCTs have many strengths, such as the use of rigorous methodologies, detailed protocols, pre-specified statistical analyses and well-defined patient cohorts (Beyer-Westendorf et al., 2016). However, RCTs are usually underpowered for subgroups analyses and run too short for (long-term) safety outcomes, do not take into account the complexity of real-world clinical decision-making, and difficult-to-reach populations tend to be underrepresented due to ethical and practical considerations (Beyer-Westendorf et al., 2016; Maetens et al., 2016; Camm et al., 2018). The included observational studies tackle these shortcoming in part, including large vulnerable patient subgroups with long follow-up in a real-world setting. However, when comparing different studies in geriatric AF patients, several limitations were present influencing the interpretability of results.

First, lack of power due to small sample sizes was present in most studies, which frequently resulted in pooling data of all NOACs despite differential safety results. Some studies also included OAC-experienced patients, which may lead to healthy user bias (Giustozzi et al., 2019). Second, NOACs dosages differed across studies. For example, rivaroxaban was used in lower dosages in Japan than approved in Europe (15 and 10 mg as standard and reduced dose respectively) (Hori et al., 2012; Group JCSJW, 2014). Likewise, 75 mg twice daily is the approved reduced dosage of dabigatran in the US (Pradaxa), whereas 110 mg twice daily in Europe (Steffel et al., 2018). Moreover, differences in off-label NOAC over- or underdosing in observational studies complicated the comparability of results (Alcusky et al., 2020; Raposeiras-Roubín et al., 2020). Third, most results were compared to warfarin, but also other VKAs such as phenprocoumon were sometimes used. Besides VKAs, other studies used aspirin, no OAC or non-AF patients as comparator arm, necessitating exclusion of these studies. Fourth, outcomes varied notably, with studies investigating ischemic stroke, overall stroke, stroke/TIA, stroke/SE, stroke/TIA/SE or stroke/SE/myocardial infarction as effectiveness outcome. Likewise, primary safety endpoints varied, from location-specific bleeding, major bleeding, major or clinically relevant non-major bleeding to any bleeding. These differential outcomes made comparisons between studies difficult. Fifth, many included observational studies were performed in an Asian setting. However, results from Asian studies cannot always be automatically generalized to other populations. For example, Asian AF patients seem to have higher stroke rates (especially haemorrhagic stroke) than Caucasian AF patients, and are also more prone to warfarin-related major bleeding events, especially intracranial bleeding (Hori et al., 2013; Chiang et al., 2014; Chao et al., 2019). Japanese guidelines therefore recommend a target INR of 1.6–2.6 in AF patients ≥70 years old [instead of 2.0–3.0 in Western countries] (Hori et al., 2012; Group JCSJW, 2014; Steffel et al., 2018). Moreover, the mean TTR in warfarin-treated Asian patients tends to be lower than in Caucasian patients (Chiang et al., 2014; Piccini et al., 2014; Chao et al., 2019). Due to these underlying ethnic differences, NOACs tend to have a better effectiveness and safety than VKAs in Asian patients. Sixth, the classification of the geriatric patient subgroups varied across studies. For example, the assessment methods of frailty varied across studies, identifying frailty based on a questionnaire (Gullón et al., 2019), clinical frailty score (Shinohara et al., 2019) or a healthcare claims-based scoring algorithm (Segal et al., 2017). Likewise, different definitions for polypharmacy and multimorbidity were used, limiting the comparability of results (Jaspers Focks et al., 2016; Piccini et al., 2016). Lastly, differences in design and selection bias may have also influenced results. For example, in the RE-LY trial, no pre-specified dose reduction criteria for dabigatran were defined, resulting in approximately similar numbers of dabigatran 110 and 150 mg users in older AF patients, due to randomization (Connolly et al., 2009; Eikelboom et al., 2011). This potential inappropriate use of standard dose dabigatran may have resulted in worse adverse outcomes in older AF patients. Moreover, selection bias due to differences in baseline characteristics of the included trial population may have affected safety results. Exemplary, the median CHADS2 score ranged from 2.1 in the ARISTOTLE trial (Granger et al., 2011) to 3.5 in the ROCKET AF trial (Patel et al., 2011), which may suggest inclusion of healthier AF patients in the ARISTOTLE trial. Similarly, only 13.9% of older subjects required a reduced dose of apixaban in the ARISTOTLE trial (Halvorsen et al., 2014). Importantly, when assessing the quality of studies using the quality assessment tool ‘QUALSYST’ (Kmet et al., 2004), *post hoc* analyses of the RE-LY (Eikelboom et al., 2011; Lauw et al., 2017) and ARISTOTLE (Halvorsen et al., 2014) trial lacked the comparison between the baseline characteristics of NOAC versus VKA users in the subgroup of patients ≥75 years old, since only overall characteristics of this subgroup were reported. Included observational studies (Avgil-Tsadok et al., 2016; Hohmann et al., 2019; Alcusky et al., 2020; Chao et al., 2020; Wong et al., 2020) frequently lacked well defined outcomes which are robust to measurement bias or were limited in their controlling for important confounders.

### Recommendation for Clinical Practice

Overall, across characteristics typical for vulnerable geriatric AF patients, apixaban was consistently associated with the best efficacy and safety profile and appears to be therefore preferred in geriatric patients. Although edoxaban ranked second on safety endpoints and third on efficacy outcomes in AF patients ≥75 years old (Deng et al., 2020), data are lacking on the impact of other geriatric patient characteristics, limiting the generalizability of the reassuring edoxaban results in older patients to all multimorbid, frail AF patients with polypharmacy. Dabigatran appears to have a more intermediate place in geriatric AF patients, especially due to the frequently noted higher gastrointestinal bleeding risks. Despite solid effectiveness results, rivaroxaban was mostly associated with worse safety outcomes across geriatric patient subgroups, due to similar intracranial and higher gastrointestinal bleeding risks.

Besides continuing and appropriately dosing NOACs, meticulous attention has to be addressed to recognizing and tackling modifiable bleeding risk factors such as hypertension, non-indicated NSAID or antiplatelet use, and excessive alcohol consumption (Kirchhof et al., 2016; Steffel et al., 2018). Moreover, prevention and management of falls using strength, balance and gait training; walking aids; correction of environmental hazards (e.g. loose carpets); and correction of footwear or structural impairments of the feet, are essential in the general approach of these geriatric patients, especially at high risk of falling (Avin et al., 2015). Furthermore, therapy adherence in community-dwelling AF patients, especially with cognitive impairment, should be optimized, for example by using weekly tablet boxes, electronically monitored medication dispensing systems or administration by a home health nurse or family member (Steffel et al., 2018). In addition, a thorough medication review and switching or discontinuing unnecessary, interacting or contraindicated comedication should be the cornerstone of management of older AF patients, especially with polypharmacy, in order to increase therapy adherence and avoid potential clinically relevant drug-drug interactions (DDIs). To identify and address the presence of (multiple) DDIs, the 2018 European Heart Rhythm Association (EHRA) guidelines have made a practical guide on NOAC dosing in patients using interacting drugs (Steffel et al., 2018). Lastly, an individual benefit-risk assessment and shared decision making must always be the cornerstone of clinical practice when deciding on whether a vulnerable geriatric patient with AF should be anticoagulated or not. Severe cases, such as patients suffering major non-traumatic intracranial haemorrhage, highly repetitive falling due to generalized epilepsy or severe multisystem atrophy, or severely frail patients with limited life expectancy, should warrant OAC discontinuation (Kirchhof et al., 2016). Performing a comprehensive geriatric assessment (CGA) in hospitalized older patients with AF may help guide clinicians in this individual benefit-risk assessment (Ellis et al., 2011).

### Research Gaps

Although a vast amount of evidence on the impact of increased age on the efficacy and safety of OACs was present, data are substantially lacking on the impact of most other geriatric patient subgroup characteristics. This systematic review has identified considerable research gaps on the impact of high falling risk, frailty and especially baseline dementia on NOAC effectiveness and safety. Moreover, more research on the impact of the number of baseline comorbidities to identify multimorbidity, as well as post-surveillance data on edoxaban in other than high age geriatric subgroups are needed.

## Conclusion

Increased age, multimorbidity, polypharmacy, high falling risk, frailty and dementia are no formal contraindications for anticoagulation in geriatric AF patients, since the benefit-risk profile of NOAC as compared to VKAs remained consistently favourable in these patient subgroups. Indeed, our meta-analysis highlighted a superior effectiveness and non-inferior safety profile of NOACs in AF patients ≥75 years old as compared to VKAs. Instead of off-label underdosing or discontinuing OACs, physicians should tackle modifiable bleeding risk factors, optimize therapy adherence, initialize fall prevention, execute a thorough medication review and perform an individualized benefit-risk assessment with shared decision making in each geriatric AF patient. Importantly, apixaban was consistently associated with the most favourable benefit-risk profile across subgroups and should therefore be preferred in geriatric AF patients. However, regarding the impact of high falling risk, frailty and baseline dementia, important research gaps were identified, necessitating more research on these topics.

## Author Contributions

MG and LL contributed to the concept and design of the systematic review. MG performed the literature search, statistical analysis, interpretation and writing. SS, TB, MP, and LL revised the systematic review critically. All authors contributed to the article and approved the submitted version.

## Funding

MG was supported by grants from the Fund for Scientific Research Flanders (FWO) project 11C0820N.

## Conflict of Interest

The authors declare that the research was conducted in the absence of any commercial or financial relationships that could be construed as a potential conflict of interest.
