# Supplementary material for: Effectiveness and Safety of Oral Anticoagulants in Older Patients With Atrial Fibrillation: A Systematic Review and Meta-Analysis
Source: Front Pharmacol. 2020 Sep 9;11:583311. doi: 10.3389/fphar.2020.583311 (PMC7509201; doi:10.3389/fphar.2020.583311)
Supplement: Supplementary file 1 [file DataSheet_1.pdf]

Supplementary Material

Table of contents

1 Supplemental tables 3
1.1 eTable 1: Search strategy 3
1.2 eTable 2: Increased age 4
1.3 eTable 3: Multimorbidity 14
1.4 eTable 4: Polypharmacy 19
1.5 eTable 5: High falling risk 21
1.6 eTable 6: Frailty 22
1.7 eTable 7: Baseline dementia 23
1.8 eTable 8: Assessment of bias within studies 24
1.9 eTable 9: PRISMA 2009 checklist 37
2 Supplemental figures 39
2.1 eFigure 1: Sensitivity analysis for all-cause mortality 39
2.2 eFigure 2: Sensitivity analyses for major bleeding 40
2.3 eFigure 3: Sensitivity analyses for gastrointestinal bleeding 42
2.4 eFigure 4: Subgroup analyses including studies with patients ≥75, ≥80, ≥85 or ≥90 years old. 44
2.5 eFigure 5: Assessment of publication bias at outcome level 47
3 References 50

1 Supplemental tables

1.1 eTable 1: Search strategy

The following search terms were used:

**P**atient	Atrial Fibrillation"[Mesh] OR "Atrial Fibrillation"[TIAB]
**I**ntervention and **C**ontrol	“Apixaban"[TIAB] OR "Apixaban"[Supplementary Concept] OR "Rivaroxaban"[Mesh] OR "Rivaroxaban"[TIAB] OR "Edoxaban"[TIAB] OR “Edoxaban"[Supplementary Concept] OR "Dabigatran"[Mesh] OR "Dabigatran"[TIAB] OR "Antithrombins"[Mesh] OR "Factor Xa Inhibitors"[Mesh] OR "New oral anticoagulants"[TIAB] OR "NOAC"[TIAB] OR "Direct oral anticoagulants"[TIAB] OR "DOAC"[TIAB] OR "Non-vitamin K antagonist oral anticoagulants"[TIAB]
**O**utcome	"Thromboembolism"[Mesh] OR "Thromboembolism"[TIAB] OR "Thrombosis"[TIAB] OR "Stroke"[TIAB] OR "Hemorrhage"[TIAB] OR "Hemorrhage"[Mesh] OR "Bleeding"[TIAB]
Filter	English

eTable 1: Search strategy.

1.2 eTable 2: Increased age

Author	Study design	Study cohort	n	Mean/median age (years +/- SD; [IQR])	Mean/median follow-up (+/- SD; [IQR])	Stroke/SE (HR [95% CI])	Major bleeding (HR [95% CI])	Intracranial bleeding (HR [95% CI])	Gastrointestinal bleeding (HR [95% CI])	All-cause mortality (HR [95% CI])
Eikelboom et al. 2011[(1)]	Phase III RCT (worldwide)	AF patients included in the RE-LY trial (dabi vs warf), **≥75y** old. Industry-sponsored.	7258	71.4y +/- 8.6 (dabi 110); 71.5y +/- 8.8 (dabi 150); 71.6y +/- 8.6 (warf); (overall, no separate results in ≥75y group)	2 years	Dabi 150 vs warf:<br>**0.67 [0.49-0.90]**<br>Dabi 110 vs warf:<br>0.88 [0.66-1.17]	Dabi 150 vs warf:<br>1.18 [0.98-1.42]<br>Dabi 110 vs warf:<br>1.01 [0.83-1.23]	Dabi 150 vs warf:<br>**0.42 [0.25-0.70]**<br>Dabi 110 vs warf:<br>**0.37 [0.21-0.64]**	Dabi 150 vs warf:<br>*1.79 [1.35-2.37]*<br>Dabi 110 vs warf:<br>*1.39 [1.03-1.98]*	NR
Lauw et al. 2017[(2)]	Phase III RCT (worldwide)	AF patients included in the RE-LY trial (dabi vs warf), categorized according to age **75-79y, 80-84y and ≥85y**. Industry-sponsored.	**75-79y:**<br>4231;<br>**80-84y:**<br>2305;<br>**≥85y:**<br>722	**75-79y:**<br>76.8y +/- 1.4;<br>**80-84y:**<br>81.7y +/- 1.4;<br>**≥85y:**<br>86.8y +/- 2.2	2 years	**75-79y:**<br>Dabi 150 vs warf:<br>0.65 [0.42-1.01]<br>Dabi 110 vs warf:<br>1.08 [0.73-1.60]<br>**80-84y:**<br>Dabi 150 vs warf:<br>0.67 [0.41-1.10]<br>Dabi 110 vs warf:<br>0.75 [0.46-1.23]<br>**≥85y:**<br>Dabi 150 vs warf:<br>0.70 [0.31-1.57]<br>Dabi 110 vs warf:<br>0.52 [0.21-1.29]	**75-79y:**<br>Dabi 150 vs warf:<br>1.04 [0.81-1.35]<br>Dabi 110 vs warf:<br>0.93 [0.71-1.21]<br>**80-84y:**<br>Dabi 150 vs warf:<br>*1.41 [1.02-1.94]*<br>Dabi 110 vs warf:<br>1.18 [0.84-1.65]<br>**≥85y:**<br>Dabi 150 vs warf:<br>1.22 [0.74-2.02]<br>Dabi 110 vs warf:<br>1.01 [0.59-1.73]	**75-79y:**<br>Dabi 150 vs warf:<br>**0.23 [0.09-0.60]**<br>Dabi 110 vs warf:<br>0.51 [0.25-1.04]<br>**80-84y:**<br>Dabi 150 vs warf:<br>0.55 [0.25-1.21]<br>Dabi 110 vs warf:<br>**0.30 [0.11-0.82]**<br>**≥85y:**<br>Dabi 150 vs warf:<br>0.61 [0.20-1.87]<br>Dabi 110 vs warf:<br>0.13 [0.02-1.04]	***Extracranial bleeding:***<br>**75-79y:**<br>Dabi 150 vs warf:<br>1.22 [0.93-1.61]<br>Dabi 110 vs warf:<br>1.03 [0.78-1.37]<br>**80-84y:**<br>Dabi 150 vs warf:<br>*1.68 [1.18-2.41]*<br>Dabi 110 vs warf:<br>*1.50 [1.03-2.18]*<br>**≥85y:**<br>Dabi 150 vs warf:<br>1.41 [0.80-2.49]<br>Dabi 110 vs warf:<br>1.32 [0.73-2.38]	**75-79y:**<br>Dabi 150 vs warf:<br>0.82 [0.63-1.07]<br>Dabi 110 vs warf:<br>0.86 [0.66-1.11]<br>**80-84y:**<br>Dabi 150 vs warf:<br>1.16 [0.87-1.55]<br>Dabi 110 vs warf:<br>**≥85y:**<br>Dabi 150 vs warf:<br>1.15 [0.74-1.79]<br>Dabi 110 vs warf:<br>1.37 [0.89-2.11]
Halperin et al. 2014[(3)]	Phase III RCT (worldwide)	AF patients included in the ROCKET AF trial (riva vs warf), **≥75y** old. Industry-sponsored.	6229	79y [76-82]	696 days [507-873]	Riva vs warf:<br>0.80 [0.63-1.02]	***Major bleeding:***<br>Riva vs warf:<br>1.11 [0.92-1.34]	Riva vs warf:<br>0.80 [0.50-1.28]	Riva vs warf:<br>*NR; event rate 2.81%/y vs 1.66%/y, p-value <0.001*	NR
Hori et al. 2014[(4)]	Phase III RCT (Japan)	AF patients included in the J-ROCKET AF trial (riva 15/10 vs warf), **≥75y** old. Industry-sponsored.	252 riva, 246 warf	79y	30 months (maximum, mean follow-up NR)	Riva vs warf:<br>0.51 [0.20-1.27]	***Major bleeding:***<br>Riva vs warf:<br>1.51 [0.68-3.32]	NR	NR	NR
Halvorsen et al. 2014[(5)]	Phase III RCT (worldwide)	AF patients included in the ARISTOTLE trial (api vs warf), **≥75y and ≥80y** old. Industry-sponsored.	**≥75y:**<br>5678 (790 api 2.5 mg);<br>**≥80y:**<br>2436	NR	1.8 years [1.4-2.3]	**≥75y:**<br>Api vs warf:<br>**0.71 [0.53-0.95]**<br>Api 5 vs warf:<br>0.75 [0.55-1.03]<br>Api 2.5 vs warf:	**≥75y:**<br>Api vs warf:<br>**0.64 [0.52-0.79]**<br>Api 5 vs warf:<br>**0.66 [0.53-0.83]**<br>Api 2.5 vs warf:	**≥75y:**<br>Api vs warf:<br>**0.34 [0.20-0.57]**<br>**≥80y:**<br>Api vs warf:<br>**0.36 [0.17-0.77]**	NR	**≥75y:**<br>Api vs warf:<br>0.91 [0.77-1.07]<br>**≥80y:**<br>Api vs warf:<br>NR

						0.52 [0.25-1.08]<br>**≥80y:**<br>Api vs warf:<br>0.81 [0.51-1.29]	**0.55 [0.31-0.94]**<br>**≥80y:**<br>Api vs warf:<br>**0.66 [0.48-0.90]**			
Kato et al. 2016[6]	Phase III RCT (worldwide)	AF patients included in the ENGAGE AF-TIMI 48 trial (edo vs warf), **≥75y, ≥80y and ≥85y** old. Industry-sponsored.	**≥75y:**<br>8474 (3488 edo 30);<br>**≥80y:**<br>3591;<br>**≥85y:**<br>899	79y [76.0-82.0]	2.8 years	**≥75y:**<br>Edo vs warf:<br>0.83 [0.66-1.04]<br>Edo 60 vs warf:<br>0.82 [0.60-1.12]<br>Edo 30 vs warf:<br>0.84 [0.61-1.15]<br>**≥80y:**<br>Edo vs warf:<br>0.88 [0.64-1.20]<br>**≥85y:**<br>Edo vs warf:<br>0.73 [0.40-1.33]	**≥75y:**<br>Edo vs warf:<br>**0.83 [0.70-0.99]**<br>Edo 60 vs warf:<br>1.06 [0.84-1.33]<br>Edo 30 vs warf:<br>**0.58 [0.43-0.77]**<br>**≥80y:**<br>Edo vs warf:<br>**0.75 [0.58-0.98]**<br>**≥85y:**<br>Edo vs warf:<br>**0.58 [0.35-0.94]**	**≥75y:**<br>Edo vs warf:<br>**0.40 [0.26-0.62]**<br>**≥80y:**<br>Edo vs warf:<br>**0.41 [0.22-0.77]**<br>**≥85y:**<br>Edo vs warf:<br>0.61 [0.20-1.88]	**≥75y:**<br>Edo vs warf:<br>*1.32 [1.01-1.72]*<br>**≥80y:**<br>Edo vs warf:<br>1.44 [0.97-2.13]<br>**≥85y:**<br>Edo vs warf:<br>0.76 [0.39-1.50]	NR
Ruff et al. 2014[7]	Meta-analysis	Pooled data of 4 phase III RCTs in AF, standard dose NOACs vs warfarin, **≥75y** old.	11188 NOAC,<br>11095 warf	71.6y NOAC, 71.5y warf (overall, NR for ≥75y subgroup)	2.2 years	Standard dose NOAC vs warf:<br>**RR 0.78 [0.68-0.88]**	Standard dose NOAC vs warf:<br>RR 0.93 [0.74-1.17]	NR	NR	NR
Sadlon et al. 2016[8]	Network meta-analysis	Pooled data of 8 phase III RCTs (4 in AF, 4 in VTE, separate data on AF reported), **≥75y** old. Specifically for comparisons with dabigatran 110 mg, the low-dose edoxaban regimen (30/15 mg) was used.	**≥75y AF:**<br>11236 NOAC,<br>11145 warf	NR	1.8-2.8 years (AF)	**≥75y AF:**<br>NOAC vs warf:<br>**OR 0.71 [0.62-0.82]**<br>Api vs dabi 150:<br>OR 1.07 [0.70-1.64]<br>Api vs dabi 110:<br>OR 0.80 [0.53-1.21]<br>Api vs riva:<br>OR 0.88 [0.60-1.29]<br>Edo 60 vs api:<br>OR 0.89 [0.59-1.36]<br>Edo 60 vs dabi 150:<br>OR 0.96 [0.62-1.47]<br>Edo 30/15 (low-dose regimen) vs dabi 110:<br>OR 1.07 [0.72-1.59]<br>Edo 60 vs riva:<br>OR 0.79 [0.54-1.16]<br>Riva vs dabi 150:<br>OR 1.21 [0.82-1.80]<br>Riva vs dabi 110:<br>OR 0.91 [0.62-1.32]	***Major bleeding or CRNMB:***<br>**≥75y AF:**<br>NOAC vs warf:<br>OR 0.98 [0.90-1.06] (I^2 89%)<br>Api vs dabi 150:<br>**OR 0.54 [0.41-0.73]**<br>Api vs dabi 110:<br>**OR 0.63 [0.47-0.86]**<br>Api vs riva:<br>**OR 0.57 [0.45-0.73]**<br>Edo 60 vs api:<br>OR 1.23 [0.93-1.64]<br>Edo 60 vs dabi 150:<br>**OR 0.67 [0.51-0.89]**<br>Edo 30/15 (low-dose regimen) vs dabi 110:	NR	NR	NR

							**OR 0.46 [0.34-0.62]**<br>Edo 60 vs riva:<br>**OR 0.71 [0.57-0.89]**<br>Riva vs dabi 150:<br>OR 0.95 [0.75-1.20]<br>Riva vs dabi 110:<br>OR 1.10 [0.86-1.41]			
Caldeira et al. 2019[9]	Meta-analysis	Pooled data of 4 phase III RCTs in AF, **≥75y** old.	13576 NOAC, 11133 warf	NR	1.8-2.8 years	NOAC vs warf:<br>**RR 0.70 [0.61-0.80]**	NOAC vs warf:<br>RR 0.91 [0.72-1.16] (I² 86%)	NR	NR	NR
Kim et al. 2018[10]	Meta-analysis	Pooled data of 5 phase III RCTs in AF, **≥75y and ≥80y** old.	**≥75y:**<br>16704 NOAC, 11433 warf;<br>**≥80y:**<br>NR	NR	1.8-2.8 years	**≥75y:**<br>NOAC vs warf:<br>**RR 0.83 [0.69-1.00], p-value 0.04**<br>Standard dose NOAC vs warf:<br>**RR 0.78 [0.69-0.90]**<br>Reduced dose NOAC vs warf:<br>RR 0.99 [0.71-1.37]<br><br>**≥80y:**<br>Standard dose NOAC vs warf:<br>**RR 0.68 [0.47-0.97]**<br>Reduced dose NOAC vs warf:<br>RR 0.72 [0.40-1.31]	**≥75y:**<br>NOAC vs warf:<br>RR 0.90 [0.68-1.19]<br>Standard dose NOAC vs warf:<br>RR 0.91 [0.72-1.15] (I² 85%)<br>Reduced dose NOAC vs warf:<br>RR 0.87 [0.45-1.70] (I² 94%)<br><br>**≥80y:**<br>Standard dose NOAC vs warf:<br>RR 0.89 [0.62-1.26]<br>Reduced dose NOAC vs warf:<br>RR 0.59 [0.30-1.18]	**≥75y:**<br>Standard dose NOAC vs warf:<br>**RR 0.49 [0.35-0.69]**<br>Reduced dose NOAC vs warf:<br>**RR 0.42 [0.29-0.61]**<br><br>**≥80y:**<br>NR	**≥75y:**<br>Standard dose NOAC vs warf:<br>*RR 1.53 [1.27-1.85]*<br>Reduced dose NOAC vs warf:<br>RR 1.04 [0.56-1.95]<br><br>**≥80y:**<br>NR	**≥75y:**<br>NOAC vs warf:<br>**RR 0.91 [0.83-1.00], p-value 0.05**<br>Standard dose NOAC vs warf:<br>**RR 0.93 [0.86-1.00], p-value 0.04**<br>Reduced dose NOAC vs warf:<br>RR 0.88 [0.61-1.27]<br>**≥80y:**<br>Standard dose NOAC vs warf:<br>**RR 0.86 [0.75-0.99]**<br>Reduced dose NOAC vs warf:<br>**RR 0.87 [0.75-1.00], p-value 0.05**
Malik et al. 2019[11]	Network meta-analysis	Pooled data of 5 phase III RCTs in AF, **≥75y** old.	27639	NR	1.8-2.8 years	NOAC vs warf:<br>**0.76 [0.67-0.86]**<br>Api vs dabi 150:<br>1.06 [0.70-1.62]<br>Api vs dabi 110:<br>0.81 [0.54-1.21]<br>Api vs riva:<br>0.91 [0.63-1.33]	NOAC vs warf:<br>0.95 [0.74-1.23] (I² 84%)<br>Api vs dabi 150:<br>**0.54 [0.41-0.72]**<br>Api vs dabi 110:<br>**0.63 [0.48-0.84]**<br>Api vs riva:	NOAC vs warf:<br>**0.48 [0.34-0.67]**<br>Api vs dabi 150:<br>0.81 [0.39-1.69]<br>Api vs dabi 110:<br>0.92 [0.43-1.97]<br>Api vs riva:<br>**0.44 [0.22-0.86]**	NR	NR

						Edo 60 vs api:<br>1.17 [0.81-1.69]<br>Edo 60 vs dabi 150:<br>1.24 [0.85-1.81]<br>Edo 60 vs dabi 110:<br>0.94 [0.65-1.36]<br>Edo 60 vs riva:<br>1.07 [0.77-1.48]<br>Riva vs dabi 150:<br>1.16 [0.79-1.70]<br>Riva vs dabi 110:<br>0.88 [0.61-1.28]<br>**Rank probability*:**<br>1: dabi 150 (83%)<br>2: api (74%)<br>3: riva (58%)<br>4: edo (45%)<br>5: dabi 110 (34%)<br>6: warf (6%)	**0.57 [0.43-0.75]**<br>Edo 60 vs api:<br>1.30 [0.99-1.70]<br>Edo 60 vs dabi 150:<br>**0.70 [0.55-0.91]**<br>Edo 60 vs dabi 110:<br>0.82 [0.63-1.07]<br>Edo 60 vs riva:<br>**0.74 [0.57-0.95]**<br>Riva vs dabi 150:<br>0.96 [0.74-1.24]<br>Riva vs dabi 110:<br>1.12 [0.85-1.46]<br>**Rank probability*:**<br>1: api (99%)<br>2: edo (79%)<br>3: warf (48%)<br>4: dabi 110 (44%)<br>5: riva (19%)<br>6: dabi 150 (11%)	Edo 60 vs api:<br>1.18 [0.60-2.32]<br>Edo 60 vs dabi 150:<br>0.95 [0.49-1.87]<br>Edo 60 vs dabi 110:<br>1.08 [0.53-2.19]<br>Edo 60 vs riva:<br>**0.51 [0.28-0.95]**<br>Riva vs dabi 150:<br>1.86 [0.94-3.66]<br>Riva vs dabi 110:<br>*2.11 [1.04-4.29]*<br>**Rank probability*:**<br>1: api (79%)<br>2: dabi 110 (72%)<br>3: edo (65%)<br>4: dabi 150 (61%)<br>5: riva (19%)<br>6: warf (0.3%)		
Lin et al. 2015[12]	Network meta-analysis	Pooled data of 25 RCTs and 24 observational studies in AF, 65-74y and **≥75y** old.	897748 (overall, ≥75y NR)	NR	NR	**≥75y:**<br>Dabi 150 vs warf:<br>**RR 0.74 [0.55-0.98]**<br>Dabi 110 vs warf:<br>RR 0.92 [0.69-1.22]<br><br>Riva vs warf:<br>RR 0.80 [0.64-1.00]<br>Api vs warf:<br>**RR 0.69 [0.53-0.90]**<br>Edo vs warf:<br>RR 0.83 [0.66-1.03]<br>Api vs dabi 150:<br>RR 0.94 [0.64-1.39]<br>Api vs dabi 110:<br>RR 0.75 [0.50-1.11]<br>Api vs riva:<br>RR 0.87 [0.61-1.23]<br>Edo vs api:<br>RR 1.20 [0.85-1.69]<br>Edo vs dabi 150:<br>RR 1.12 [0.78-1.61]<br>Edo vs dabi 110:	**≥75y:**<br>Dabi 150 vs warf:<br>RR 1.17 [0.99-1.39]<br><br>Dabi 110 vs warf:<br>RR 1.02 [0.84-1.23]<br>Riva vs warf:<br>RR 1.12 [0.93-1.36]<br>Api vs warf:<br>**RR 0.67 [0.55-0.82]**<br>Edo vs warf:<br>RR 0.84 [0.69-1.01]<br>Api vs dabi 150:<br>**RR 0.57 [0.44-0.75]**<br>Api vs dabi 110:	**≥75y:**<br>Dabi 150 vs warf:<br>**RR 0.39 [0.29-0.51]**<br><br>Dabi 110 vs warf:<br>**RR 0.36 [0.21-0.62]**<br>Riva vs warf:<br>RR 0.79 [0.51-1.23]<br>Api vs warf:<br>**RR 0.33 [0.20-0.56]**<br>Edo vs warf:<br>**RR 0.41 [0.27-0.62]**<br>Api vs dabi 150:<br>RR 0.87 [0.48-1.55]<br>Api vs dabi 110:	**≥75y:**<br>Dabi 150 vs warf:<br>*RR 1.51 [1.16-1.96]*<br><br>Dabi 110 vs warf:<br>RR 1.27 [0.78-2.07]<br>Riva vs warf:<br>RR 1.16 [0.81-1.66]<br>Riva vs dabi 150:<br>RR 0.77 [0.49-1.20]<br>Riva vs dabi 110:<br>RR 0.91 [0.50-1.68]<br><br>Otherwise NR	**≥75y:**<br>Dabi 150 vs warf:<br>RR 0.89 [0.78-1.02]<br><br>Api vs warf:<br>RR 0.89 [0.76-1.04]<br>Api vs dabi 150:<br>RR 1.00 [0.82-1.23]<br><br>Otherwise NR

						RR 0.90 [0.63-1.29]<br>Edo vs riva:<br>RR 1.04 [0.76-1.43]<br>Riva vs dabi 150:<br>RR 1.08 [0.75-1.55]<br>Riva vs dabi 110:<br>RR 0.87 [0.60-1.24]	**RR 0.66 [0.50-0.87]**<br>Api vs riva:<br>**RR 0.60 [0.45-0.79]**<br>Edo vs api:<br>RR 1.25 [0.95-1.64]<br>Edo vs dabi 150:<br>**RR 0.71 [0.55-0.92]**<br>Edo vs dabi 110:<br>RR 0.82 [0.63-1.08]<br>Edo vs riva:<br>**RR 0.74 [0.57-0.97]**<br>Riva vs dabi 150:<br>RR 0.96 [0.75-1.24]<br>Riva vs dabi 110:<br>RR 1.11 [0.85-1.45]	RR 0.92 [0.44-1.93]<br>Api vs riva:<br>**RR 0.42 [0.21-0.83]**<br>Edo vs api:<br>RR 1.23 [0.64-2.38]<br>Edo vs dabi 150:<br>RR 1.07 [0.64-1.76]<br>Edo vs dabi 110:<br>RR 1.13 [0.57-2.24]<br>Edo vs riva:<br>**RR 0.52 [0.28-0.95]**<br>Riva vs dabi 150:<br>*RR 2.06 [1.22-3.46]*<br>Riva vs dabi 110:<br>*RR 2.18 [1.09-4.38]*		
Deng et al. 2020[13]	Network meta-analysis	Pooled data of 5 phase III RCTs in AF, ≥75y old.	28137	NR	1.8-2.8 years	**Rank probability*:**<br>1. api 5 (41.2%)<br>2. riva 20 (31.8%)<br>3. edo 60 (15.9%)<br>4. dabi 110 (10.9%)<br>5. warf (0.2%)	**Rank probability*:**<br>1. api 5 (71.4%)<br>2. edo 60 (21.0%)<br>3. dabi 110 (5.8%)<br>4. warf (0.9%)<br>5. riva 20 (0.8%)	NR	NR	NR
Chao et al. 2020[14]	Observational retrospective nationwide cohort study (Taiwan)	AF patients 65-74y, **75-89y and ≥90y** old from administrative claims database, OAC-naïve and –experienced, NOAC (dabi, riva, api) vs warf	**75-89y:**<br>28179 NOAC, 10609 warf<br>**≥90:**<br>3283 NOAC, 1497 warf	**75-89y:**<br>84.4y +/- 4.1 NOAC, 81.5y +/- 4.1 warf<br>**≥90y:**<br>92.4y +/- 2.5 NOAC, 92.5y +/- 2.6 warf	Maximum 3 years (median NR)	***Ischemic stroke***<br>**75-89y:**<br>NOAC vs warf:<br>**0.83 [0.76-0.90]**<br>**≥90y:**<br>NOAC vs warf:<br>0.90 [0.71-1.13]	**75-89y:**<br>NOAC vs warf:<br>**0.86 [0.80-0.92]**<br>**≥90y:**<br>NOAC vs warf:<br>0.86 [0.72-1.03]	**75-89y:**<br>NOAC vs warf:<br>**0.56 [0.47-0.67]**<br>**≥90y:**<br>NOAC vs warf:<br>**0.36 [0.23-0.58]**	NR	**75-89y:**<br>NOAC vs warf:<br>**0.50 [0.48-0.53]**<br>**≥90y:**<br>NOAC vs warf:<br>**0.58 [0.52-0.64]**
Deitelzweig et al. 2019[15]	Observational retrospective nationwide cohort study (USA)	AF patients **≥80y old**, included in ARISTOPHANES study (4 commercial claims databases),	53710 NOAC, 49801 warf; 6x 1:1 PSM cohorts:	84.8y +/- 3.8 – 85.3y +/- 4.0	5-6 months	***Stroke/SE:***<br>Dabi vs warf:<br>**0.77 [0.60-0.99]**<br>Riva vs warf:<br>**0.74 [0.65-0.85]**	Dabi vs warf:<br>0.92 [0.78-1.07]<br>Riva vs warf:<br>*1.16 [1.07-1.24]*<br>Api vs warf:	Dabi vs warf:<br>**0.51 [0.33-0.79]**<br>Riva vs warf:<br>**0.78 [0.64-0.95]**<br>Api vs warf:	Dabi vs warf:<br>1.17 [0.94-1.46]<br>Riva vs warf:<br>*1.33 [1.20-1.47]*<br>Api vs warf:	Dabi vs warf:<br>**0.87 [0.75-0.99]**<br>Riva vs warf:<br>**0.87 [0.81-0.93]**<br>Api vs warf:

		OAC-naïve, NOAC (reduced dose in 52% api, 37% dabi, 51% riva) vs warf. Industry-sponsored.	13396 dabi-warf, 51834 riva-warf, 37794 api-warf, 12954 api-dabi, 37116 api-riva, 13366 dabi-riva			Api vs warf:<br>**0.58 [0.49-0.69]**<br>Api vs dabi:<br>**0.65 [0.47-0.89]**<br>Api vs riva:<br>**0.72 [0.59-0.86]**<br>Dabi vs riva:<br>1.11 [0.84-1.46]<br><br>***Ischemic stroke:***<br>Dabi vs warf:<br>0.89 [0.66-1.19]<br>Riva vs warf:<br>**0.74 [0.64-0.86]**<br>Api vs warf:<br>**0.64 [0.53-0.78]**	**0.60 [0.54-0.67]**<br>Api vs dabi:<br>**0.60 [0.49-0.73]**<br>Api vs riva:<br>**0.50 [0.45-0.55]**<br>Dabi vs riva:<br>**0.77 [0.67-0.90]**	**0.53 [0.41-0.68]**<br>Api vs dabi:<br>0.89 [0.51-1.57]<br>Api vs riva:<br>**0.70 [0.53-0.94]**<br>Dabi vs riva:<br>0.72 [0.46-1.13]	**0.62 [0.53-0.72]**<br>Api vs dabi:<br>**0.50 [0.38-0.65]**<br>Api vs riva:<br>**0.45 [0.39-0.52]**<br>Dabi vs riva:<br>**0.74 [0.61-0.90]**	**0.61 [0.56-0.67]**<br>Api vs dabi:<br>**0.78 [0.66-0.91]**<br>Api vs riva:<br>**0.71 [0.64-0.77]**<br>Dabi vs riva:<br>0.95 [0.82-1.09]
Raposeiras-Roubín et al. 2020[16]	Observational retrospective multicenter cohort study (Spain)	AF patients **≥90y** old, included from medical records in 3 health areas (NON-AF NON-VALV project). OAC-experienced. Off-label dosing in 41.5% (35.3% under-, 6.1% overdosing), mean TTR ≥65% in only 32.5% of VKA users. Industry-sponsored.	716 NOAC (14.7% dabi, 47.3% riva, 33.1% api, 4.9% edo), 500 VKA, 534 no OAC	93.0y +/- 5.2 NOAC, 92.1y +/- 2.6 VKA, 93.5y +/- 3.6 no OAC	23.6 months +/- 6.6	***Composite stroke/TIA/SE, pulmonary embolism and death:***<br>NOAC vs no OAC:<br>**0.75 [0.61-0.92]**<br>VKA vs no OAC:<br>0.87 [0.72-1.05]	NOAC vs no OAC:<br>1.43 [0.97-2.13]<br>VKA vs no OAC:<br>*1.94 [1.31-2.88]*	NOAC vs no OAC:<br>1.59 [0.44-5.79]<br>VKA vs no OAC:<br>*4.43 [1.48-13.31]*	NR	NR
Nishida et al. 2019[17]	Observational prospective multicenter cohort study (Japan)	AF patients **75-84y and ≥85y** old, included in SAKURA AF Registry. OAC-naïve and –experienced, type of NOACs NR. Industry-sponsored.	**75-84y**:<br>569 NOAC, 509 warf;<br>**≥85y**:<br>121 NOAC, 143 warf	**75-84y**:<br>78.9y +/- 2.8<br>**≥85y**:<br>87.4y +/- 2.5	39.3 months [28.5-43.6] (overall, NR for 75-84y or ≥85y subgroups)	***Stroke/TIA/SE***<br>**75-84y:**<br>NOAC vs warf:<br>1.30 [0.73-2.33]<br>**≥85y:**<br>NOAC vs warf:<br>0.49 [0.15-1.56]	**75-84y:**<br>NOAC vs warf:<br>1.11 [0.61-2.01]<br>**≥85y:**<br>NOAC vs warf:<br>**0.22 [0.042-0.92]**	NR	NR	**75-84y:**<br>NOAC vs warf:<br>1.27 [0.92-1.97]<br>**≥85y:**<br>NOAC vs warf:<br>0.67 [0.33-1.33]
Kim et al. 2019[18]	Observational retrospective single-center cohort study (South-Korea)	AF patients **≥80y** old from database of one university hospital, NOAC (dabi, riva, api) vs warf, OAC-naïve and –experienced.	403 NOAC, 284 warf	83.4y +/- 3.2 NOAC, 83.5y +/- 3.1 warf	5.5 months [1.8-8.9] NOAC, 15.3 months [4.0-42.6]	NOAC vs warf:<br>**0.13 [0.04-0.48]**	NOAC vs warf:<br>**0.11 [0.02-0.49]**	NOAC vs warf:<br>**0.024 [0.002-0.35]**	NOAC vs warf:<br>0.37 [0.047-2.95]	NOAC vs warf:<br>**0.30 [0.11-0.82]**
Hohmann et al. 2019[19]	Observational retrospective nationwide cohort study (Germany)	AF patients <75y or **≥75y old** from administrative healthcare claims database, NOAC	**Overall:**<br>42562 NOAC, 27939 phen<br>**≥75y:**	**≥75y:**<br>81.5y +/- 4.8	**Overall:**<br>706 days +/- 378 NOAC, 856 days +/- 395 phen	**≥75y:**<br>NOAC vs phen:<br>0.97 [0.83-1.14]	***Major extracranial bleeding:***<br>NOAC vs phen:<br>**0.71 [0.58-0.85]**	**≥75y:**<br>NOAC vs phen:<br>**0.59 [0.47-0.73]**	**≥75y:**<br>NOAC vs phen:<br>1.10 [0.94-1.29]<br>Dabi vs phen:<br>0.99 [0.71-1.38]	NR

		(12.7% dabi, 56.2% riva, 31.1% api) vs phen, OAC-naïve.	37816 (NOAC and phen)		(overall, NR for ≥75y)		Dabi vs phen:<br>**0.51 [0.38-0.69]**<br>Riva vs phen:<br>0.89 [0.72-1.09]<br>Api vs phen:<br>**0.44 [0.26-0.74]**		Riva vs phen:<br>*1.44 [1.21-1.70]*<br>Api vs phen:<br>**0.64 [0.50-0.81]**	
Mitchell et al. 2019[20]	Meta-analysis	Pooled data of 20 observational studies in AF, NOACs vs warf, **≥75y** old.	428031	NR	NR	***Stroke/TIA/SE:***<br>NOAC vs warf:<br>0.93 [0.85-1.01]<br>***Ischemic stroke:***<br>NOAC vs warf:<br>**0.86 [0.75-0.99]**	NOAC vs warf:<br>0.96 [0.84-1.09]	NOAC vs warf:<br>**0.56 [0.48-0.67]**	NOAC vs warf:<br>*1.46 [1.31-1.63]*<br>*(note: only dabi and riva, no api data on GI bleeding)*	NOAC vs warf:<br>0.92 [0.77-1.10]
Russo et al. 2019[21]	Observational prospective multicenter cohort study (Italy)	AF patients **≥80y** old from AF research database, NOAC (48% riva, 26% dabi, 25% api, 1% edo) vs VKA (86% warf, 14% acenocoumarol), OAC-naïve and –experienced, ≥1y follow-up.	253 NOAC, 705 VKA (after 1:2 PSM: 252 and 504)	84.5y +/- 3.1 NOAC, 84.5y +/- 3.3 VKA (after PSM)	31.1 +/- 14.1 months	***Stroke/TIA/SE:***<br>NOAC vs warf:<br>1.10 [0.49-2.45]	NOAC vs warf:<br>0.89 [0.53-1.50]	NOAC vs warf:<br>0.33 [0.07-1.45]	NR	NOAC vs warf:<br>**0.65 [0.47-0.90]**
Shinohara et al. 2019[22]	Observational retrospective single center cohort study (Japan)	AF patients **≥80y** old with non-severe frailty (clinical frailty scale <7), included in single institution, OAC-naïve, 23.1% off-label dosing (15.4% under-, 7.7% overdosing)	273 NOAC (64 dabi, 81 riva, 100 api, 28 edo), 81 warf	83.8y +/- 3.6 (overall)	33.1 months [14.0-51.0]	NOAC vs warf:<br>0.63 [0.16-2.57]	***Major bleeding or CRNMB:***<br>NOAC vs warf:<br>**0.26 [0.07-0.91]**	NR	NR	NR
Giustozzi et al. 2019[23]	Observational prospective multicenter cohort study (Italy)	AF patients **≥90y** old, NOAC users (16.3% dabi, 49.4% riva and 34.3% api; OAC-naïve or –switcher) prospectively followed, VKA users (OAC-naïve or –experienced) retrospectively analysed.	245 NOAC (81.6% reduced dose; 128 OAC-naïve), 301 VKA (62 OAC-naïve)	91.5y +/- 1.8 NOAC;<br>92.4y +/- 2.0 VKA	Median: 404 days;<br>Mean: 596 +/- 539 days	***Stroke/TIA/SE:***<br>NOAC vs warf:<br>0.78 [0.30-2.04]	NOAC vs warf:<br>1.43 [0.77-2.65]	NR	NR	NR
Avgil-Tsadok et al. 2016[24]	Observational retrospective nationwide cohort study (Canada)	AF patients **≥75y old** from administrative healthcare claims database, dabi vs warf, OAC-naïve and –experienced.	1899 dabi 150;<br>7649 dabi 110;<br>32930 warf	78.3y +/- 9.3 (overall, no separate results in ≥75y group)	1.3 years	***Stroke/TIA:***<br>Dabi vs warf:<br>1.05 [0.93-1.19]<br>Dabi 150 vs warf:<br>1.05 [0.79-1.39]<br>Dabi 110 vs warf:	Dabi vs warf:<br>0.94 [0.86-1.01]<br>Dabi 150 vs warf:<br>0.93 [0.79-1.10]<br>Dabi 110 vs warf:<br>0.94 [0.86-1.03]	Dabi vs warf:<br>**0.60 [0.47-0.76]**<br>Dabi 150 vs warf:<br>0.79 [0.50-1.25]<br>Dabi 110 vs warf:<br>**0.55 [0.42-0.73]**	Dabi vs warf:<br>*1.30 [1.14-1.50]*<br>Dabi 150 vs warf:<br>*1.35 [1.01-1.82]*<br>Dabi 110 vs warf:<br>*1.31 [1.13-1.51]*	NR

						1.07 [0.94-1.22]				
Alcusky et al. 2020[25]	Observational retrospective nationwide cohort study (USA)	Older AF patients from administrative healthcare claims database, OAC-naïve, nursing-home residents. Off-label dosing in 33.5% api, 40.9% dabi and 55.6% riva (mostly underdosing)	1289 dabi, 3758 riva, 3422 api, warf NR (3x 1:1 PSM: 1289 dabi-warf, 3735 riva-warf, 2881 api-warf)	**Dabi-warf:** 83y [77-89] dabi, 83y [77-89] warf; **Riva-warf:** 84y [77-89] riva, 84y [77-89] warf; **Api-warf:** 84y [77-89] api 84y [76-89] warf (after 1:1 PSM)	**Dabi-warf:** 134 days [44-162] dabi, 212 days [57-580] warf; **Riva-warf:** 139 days [42-374] riva, 147 days [44-376] warf; **Api-warf:** 137 days [45-326] api, 124 days [40-285] warf	***Ischemic stroke/TIA:*** Dabi vs warf: 0.92 [0.51-1.65] Riva vs warf: 1.09 [0.73-1.63] Api vs warf: *1.86 [1.00-3.45]*	Dabi vs warf: 1.10 [0.80-1.53] Riva vs warf: 1.07 [0.87-1.33] Api vs warf: **0.66 [0.49-0.88]**	NR	NR	Dabi vs warf: **0.68 [0.59-0.79]** Riva vs warf: 0.79 [0.72-0.87] Api vs warf: **0.78 [0.70-0.88]**
Lai et al. 2018[26]	Observational retrospective nationwide cohort study (Taiwan)	AF patients **≥85y old** from administrative claims database, OAC-naïve	1489 dabi 110; 846 riva 15, 890 riva 10; 1497 warf (1:1 PSM: 1180 dabi-warf, 1207 riva-warf)	88.4y +/- 2.9 dabi; 88.8y +/- 3.1 riva; 88.7y +/- 3.1 warf (after 1:1 PSM)	6.6 months	***Ischemic stroke:*** Dabi 110 vs warf: 1.25 [0.75-2.09] Riva vs warf: 1.02 [0.64-1.65]	NR	Dabi 110 vs warf: **0.31 [0.10-0.97]** Riva vs warf: 0.47 [0.17-1.26]	Dabi 110 vs warf: 1.21 [0.76-1.91] Riva vs warf: 0.81 [0.47-1.38]	Dabi 110 vs warf: **0.59 [0.45-0.77]** Riva vs warf: **0.61 [0.47-0.79]**
Poli et al. 2019[27]	Observational prospective multicenter cohort study (Italy)	AF patients **≥85y** old, included in START2-REGISTER study, OAC-naïve and -switchers, ≥1y follow-up. Industry-sponsored.	322 NOAC (18% dabi, 34% riva, 41% api, 7% edo; 31% OAC-switcher), 660 VKA (all OAC-naïve)	88.4y +/ 2.8 NOAC, 87.4y +/- 2.2 VKA	12.7 months NOAC, 20.8 months VKA	***Stroke/TIA:*** NOAC vs warf: *4.04 [1.60-10.2]*	NOAC vs warf: 0.88 [0.42-1.80]	NOAC vs warf: 0.77%/year [0.33-1.79] for NOACs, 0.64%/year [0.34-1.23] for VKA (risk estimate NR)	NOAC vs warf: 2.00%/year [1.17-3.40] for NOACs, 0.86%/year [0.50-1.51] for VKA (risk estimate NR)	NOAC vs warf: **0.64 [0.46-0.91]**
Shah et al. 2019[28]	Markov state transition model (USA)	AF patients **≥75y** old, included from ATRIA-CVRN cohort. Net clinical benefit (NCB) in gain or loss of quality-adjusted life years (QALYs), based on the risk for stroke, bleeding and death	14946 (overall: no separate results on api, warf or no OAC)	Median 81y, range [75-106]	NR	***Median NCB (QALYs [IQR]):*** **≥75y** Api vs no OAC: 0.74 [0.49-1.06] Warf vs no OAC: 0.45 [0.25-0.72] **Significance threshold:**	NR	NR	NR	NR

		from another competing cause; NCB of 0.10 QALYs prespecified as non-significant				Api vs no OAC: **92y** (QALY 0.10 [0.07-0.13]) Warf vs no OAC: **87y** (QALY 0.10 [0.04-0.16])				
Wong et al. 2020[29]	Observational retrospective nationwide cohort study (USA)	AF patients **≥75y** old, included from NCDR PINNACLE national ambulatory registry matched to administrative healthcare claims database, OAC-naïve and –experienced.	91702 NOAC (32737 dabi, 40994 riva, 17971 api), 177318 warf, 154430 no OAC (overall, ≥75y NR)	75.5y +/- 7.3 dabi; 75.6y +/- 7.3 riva; 76.5y +/- 7.4 api; 77.3y +/- 7.5 warf; 77.1y +/- 8.5 no OAC (overall, no separate results in ≥75y group)	1.4 years +/- 0.6 (overall, no separate results in ≥75y group)	NR	NOAC vs warf: **0.93 [0.90-0.97]** Dabi vs warf: **0.83 [0.78-0.87]** Riva vs warf: *1.06 [1.01-1.12]* Api vs warf: **0.89 [0.81-0.98]**	NOAC vs warf: **0.70 [0.62-0.79]** Dabi vs warf: **0.59 [0.49-0.71]** Riva vs warf: **0.81 [0.69-0.96]** Api vs warf: **0.70 [0.53-0.94]**	NOAC vs warf: *1.10 [1.04-1.17]* Dabi vs warf: 0.95 [0.87-1.03] Riva vs warf: *1.32 [1.22-1.42]* Api vs warf: 0.93 [0.80-1.07]	NR

eTable 2: Overview of included studies investigating the impact of increased age (≥75 years) on the effectiveness and safety of oral anticoagulants.

Bold: significantly lower risk; *Italic*: significantly higher risk

*Rank probability: the rank probabilities reflect the hierarchy of drugs, with a larger first-rank probability value symbolizing that the drug is more likely to be the best.

AF: atrial fibrillation; Api 2.5: apixaban 2.5 mg (reduced dose); Api 5: apixaban 5 mg (standard dose); CI: confidence interval; CRNMB: clinically relevant non-major bleeding; Dabi 110: dabigatran 110 mg (reduced dose); Dabi 150: dabigatran 150 mg (standard dose); Edo 30: edoxaban 30 mg (reduced dose); Edo 60: edoxaban 60 mg (standard dose); HR: hazard ratio; I^2: statistic for heterogeneity between included trials in meta-analysis; IQR: interquartile range; NCB: net clinical benefit; NOAC: non-vitamin K antagonist oral anticoagulant; NR: not reported; OAC: oral anticoagulant; OR: odds ratio; Phen: phenprocoumon; PSM: propensity score matching; QALY: quality-adjusted life year; RCT: randomized controlled trial; Riva: rivaroxaban; Riva 20/15: rivaroxaban 20 mg (standard dose) and 15 mg (reduced dose); Riva 15/10: rivaroxaban 15 mg (standard dose) and 10 mg (reduced dose); RR: relative risk; SD: standard deviation; Stroke/SE: stroke/systemic embolism; TTR: time in therapeutic range (for VKA users); USA: United States of America; VKA: vitamin K antagonist; Vs: versus; Warf: warfarin; y: year

1.3 eTable 3: Multimorbidity

Author	Study design	Study cohort	n	Mean/median age (years +/- SD; [IQR])	Mean/median follow-up (+/- SD; [IQR])	Stroke/SE (HR [95% CI])	Major bleeding (HR [95% CI])	Intracranial bleeding (HR [95% CI])	Gastrointestinal bleeding (HR [95% CI])	All-cause mortality (HR [95% CI])
Alexander et al. 2019[30]	Phase III RCT (worldwide)	AF patients included in the ARISTOTLE trial (api vs warf), categorized according to the number of baseline comorbidities (17 in total): no multimorbidity (0-2), moderate multimorbidity (3-5) and high multimorbidity (≥6). Industry-sponsored.	**No multi-morbidity:** 6087; **Moderate multi-morbidity:** 8491; **High multi-morbidity:** 2222	**No multimorbidity:** 69y [63-75]; **Moderate multimorbidity:** 71y [65-77]; **High multimorbidity:** 74y [68-79]	1.8 years [1.4-2.3] (overall, no separate results in subgroups)	**Moderate multimorbidity:** Api vs warf: **0.72 [0.56-0.93]** **High multimorbidity:** Api vs warf: 0.93 [0.57-1.50]	**Moderate multimorbidity** Api vs warf: **0.67 [0.55-0.82]** **High multimorbidity** Api vs warf: 0.82 [0.59-1.13]	NR	NR	**Moderate multimorbidity** Api vs warf: 0.96 [0.82-1.13] **High multimorbidity** Api vs warf: 0.89 [0.70-1.11]
Connolly et al. 2009[31]	Phase III RCT (worldwide)	AF patients included in the RE-LY trial (dabi vs warf), categorized according to **CHADS2 score** 0-1, 2 or **≥3**. Industry-sponsored.	**CHADS2 ≥3**: 1981 dabi 150; 1968 dabi 110; 1933 warf	71.5y +/- 8.8 dabi 150, 71.4y +/- 8.6 dabi 110, 71.6y +/- 8.6 warf (overall, no separate results in CHADS2 ≥3)	2.0 years (overall, no separate results in CHADS2 ≥3)	**CHADS2 ≥3**: **1.88%/y dabi 150**, 2.12%/y dabi 110, 2.68%/y warf (incidence rates, significant CI for dabi 150 vs warf, non-significant for dabi 110 vs warf, risk estimates NR)	NR	NR	NR	NR
Oldgren et al. 2011[32]	Phase III RCT (worldwide)	AF patients included in the RE-LY trial (dabi vs warf), categorized according to **CHADS2 score** 0-1, 2 or **≥3**. Industry-sponsored.	**CHADS2 ≥3**: 1981 dabi 150; 1968 dabi 110; 1933 warf	**CHADS2 ≥3**: 73.0y +/- 9.0 (overall, no separate results in dabi 150, dabi 110 and warf)	2.0 years (overall, no separate results in CHADS2 ≥3)	**CHADS2 ≥3**: Dabi 150 vs warf: **0.69 [0.51-0.93]** Dabi 110 vs warf: 0.78 [0.58-1.04]	**CHADS2 ≥3**: Dabi 150 vs warf: 1.07 [0.87-1.31] Dabi 110 vs warf: 0.83 [0.66-1.03]	**CHADS2 ≥3**: Dabi 150 vs warf: **0.48 [0.28-0.82]** Dabi 110 vs warf: **0.24 [0.12-0.48]**	NR	**CHADS2 ≥3**: Dabi 150 vs warf: 1.02 [0.84-1.23] Dabi 110 vs warf: 0.87 [0.72-1.06]
Patel et al. 2011[33]	Phase III RCT (worldwide)	AF patients included in the ROCKET AF trial (riva vs warf), categorized according to **CHADS2 score** 2, **3, 4, 5 or 6.** Industry-sponsored.	**CHADS2 3**: 3036 riva, 3133 warf; **CHADS2 4**: 2078 riva, 1989 warf; **CHADS2 5**: 920 riva, 877 warf; **CHADS2 6**:	73y [65-78] riva, 73y [65-78] warf (overall, no separate results in CHADS2 score groups)	707 days (overall, no separate results in CHADS2 score groups)	**CHADS2 3**: Riva vs warf: 0.76 [0.57-1.01] **CHADS2 4**: Riva vs warf: 0.95 [0.72-1.24] **CHADS2 5**: Riva vs warf: 0.88 [0.58-1.34] **CHADS2 6**:	**CHADS2 3**: Riva vs warf: 1.03 [0.92-1.15] **CHADS2 4**: Riva vs warf: 0.92 [0.80-1.06] **CHADS2 5**: Riva vs warf: 1.09 [0.89-1.35] **CHADS2 6**:	NR	NR	NR

			122 riva, 156 warf			Riva vs warf:<br>1.49 [0.62-3.59]	Riva vs warf:<br>0.87 [0.53-1.44]			
Hori et al. 2014[34]	Phase III RCT (Japan)	AF patients included in the J-ROCKET AF trial (riva 15/10 vs warf), categorized according to **CHADS2 score** 2 or **≥3**. Industry-sponsored.	**CHADS2 ≥3**:<br>542 riva, 524 warf	**CHADS2 ≥3**:<br>71.6y riva, 72.2y warf	30 months (maximum duration, median follow-up NR)	**CHADS2 ≥3**:<br>Riva 15/10 vs warf:<br>0.49 [0.22-1.11]	**CHADS2 ≥3**:<br>Riva 15/10 vs warf:<br>1.11 [0.86-1.45]	NR	NR	NR
Granger et al. 2011[35]	Phase III RCT (worldwide)	AF patients included in the ARISTOTLE trial (api vs warf), categorized according to **CHADS2 score** 1, 2 or **≥3**. Industry-sponsored.	**CHADS2 ≥3**:<br>2758 api, 2744 warf	70y [63-76] api, 70y [63-76] warf (overall, no separate results in CHADS2 score groups)	1.8 years [1.4-2.3] (overall, no separate results in CHADS2 score groups)	**CHADS2 ≥3**:<br>**1.9%/y api,**<br>2.8%/y warf (incidence rates, significant CI, risk estimates NR)	**CHADS2 ≥3**:<br>**2.9%/y api,**<br>4.2%/y warf (incidence rates, significant CI, risk estimates NR)	NR	NR	NR
Lopes et al. 2012[36]	Phase III RCT (worldwide)	AF patients included in the ARISTOTLE trial (api vs warf), categorized according to **CHADS2, CHA_2DS_2-VASc or HAS-BLED score** 1, 2 or **≥3**. Industry-sponsored.	**CHADS2 ≥3**:<br>2758 api, 2744 warf;<br>**CHA_2DS_2-VASc ≥3**:<br>12826 (overall);<br>**HAS-BLED ≥3**:<br>4172 (overall)	**CHADS2 ≥3**:<br>75.0y [67-79] (overall, no separate results in CHA_2DS_2-VASc or HAS-BLED score groups)	1.8 years [1.4-2.3] (overall, no separate results in subgroups)	**CHADS2 ≥3**:<br>Api vs warf:<br>**0.70 [0.54-0.91]**<br>**CHA_2DS_2-VASc ≥3**:<br>Api vs warf:<br>**0.73 [0.60-0.89]**<br>**HAS-BLED ≥3**:<br>Api vs warf:<br>0.81 [0.58-1.13]	**CHADS2 ≥3**:<br>Api vs warf:<br>**0.70 [0.56-0.88]**<br>**CHA_2DS_2-VASc ≥3**:<br>Api vs warf:<br>**0.73 [0.63-0.86]**<br>**HAS-BLED ≥3**:<br>Api vs warf:<br>**0.74 [0.58-0.94]**	**CHADS2 ≥3**:<br>Api vs warf:<br>**0.29 [0.16-0.50]**<br>**CHA_2DS_2-VASc ≥3**:<br>Api vs warf:<br>**0.40 [0.28-0.58]**<br>**HAS-BLED ≥3**:<br>Api vs warf:<br>**0.22 [0.10-0.48]**	NR	**CHADS2 ≥3**:<br>Api vs warf:<br>0.87 [0.73-1.03]<br>**CHA_2DS_2-VASc ≥3**:<br>Api vs warf:<br>0.89 [0.79-1.01]<br>**HAS-BLED ≥3**:<br>Api vs warf:<br>**0.77 [0.62-0.96]**
Giugliano et al. 2013[37]	Phase III RCT (worldwide)	AF patients included in the ENGAGE AF-TIMI 48 trial, **CHADS2 score** ≤3 and **>3.** High-dose edo regimen (60/30 mg) vs warf. Industry-sponsored.	**CHADS2 >3**:<br>1613 high-dose edo (60/30);<br>1591 warf	72y [64-78] high-dose edo, 72y [64-78] warf (overall, no separate results in CHADS2 score groups)	2.8 years (overall, no separate results in CHADS2 score groups)	**CHADS2 >3**:<br>2.46%/y edo 60/30, 3.00%/y warf (incidence rates, non-significant CI, risk estimates NR)	**CHADS2 >3**:<br>3.79%/y edo 60/30, 4.68%/y warf (incidence rates, non-significant CI, risk estimates NR)	NR	NR	NR
Mentias et al. 2018[38]	Observational retrospective nationwide cohort study (USA)	AF patients from administrative healthcare claims database. Low, moderate or high multimorbidity based on CHA_2DS_2-VASc score 1-3, 4-5 and ≥6; HAS-BLED score 0-1, 2, ≥ 3; and the Gagne comorbidity score 0-2, 3-4, ≥5 respectively. Dabi	***Moderate multi-morbidity:***<br>**CHA_2DS_2-VASc 4-5:**<br>9631 dabi, 10253 riva, 44087 warf;<br><br>**HAS-BLED 2:**	75.83y +/- 6.4 dabi 150, 75.75y +/- 6.4 riva 20, 78.45y +/- 7.2 warf (overall, no separate results in multimorbidity groups)	Up to 4 years (maximum duration, median follow-up NR)	***Ischemic stroke***<br>**CHA_2DS_2-VASc 4-5:**<br>Dabi vs warf:<br>1.21 [0.90-1.64]<br>Riva vs warf:<br>1.06 [0.77-1.45]<br>Riva vs dabi:<br>0.87 [0.64-1.18]<br><br>**HAS-BLED 2:**	**CHA_2DS_2-VASc 4-5:**<br>Dabi vs warf:<br>0.91 [0.77-1.09]<br>Riva vs warf:<br>1.13 [0.95-1.34]<br>Riva vs dabi:<br>*1.24 [1.04-1.48]*<br><br>**HAS-BLED 2:**<br>Dabi vs warf:	NR	**CHA_2DS_2-VASc 4-5:**<br>Dabi vs warf:<br>1.06 [0.87-1.29]<br>Riva vs warf:<br>*1.30 [1.08-1.58]*<br>Riva vs dabi:<br>*1.23 [1.01-1.48]*<br><br>**HAS-BLED 2:**<br>Dabi vs warf:	**CHA_2DS_2-VASc 4-5:**<br>Dabi vs warf:<br>0.83 [0.68-1.01]<br>Riva vs warf:<br>**0.81 [0.66-0.98]**<br>Riva vs dabi:<br>0.98 [0.79-1.20]<br><br>**HAS-BLED 2:**<br>Dabi vs warf:

		150 vs riva 20 vs warf using 3-way propensity score matching (PSM). OAC-naïve.	7520 dabi, 7829 riva, 37291 warf;<br>**Gagne 3-4:** 6211 dabi, 6512 riva, 27524 warf;<br><br>***High multi-morbidity:*** **CHA_2DS_2-VASc ≥6:** 5075 dabi, 5230 riva, 36782 warf;<br>**HAS-BLED ≥3:** 2976 dabi, 3094 riva, 22347 warf;<br>**Gagne ≥5:** 4519 dabi, 4643 riva, 39348 warf;			Dabi vs warf:<br>0.76 [0.56-1.03]<br>Riva vs warf:<br>**0.69 [0.50-0.95]**<br>Riva vs dabi:<br>0.91 [0.65-1.27]<br>**Gagne 3-4:**<br>Dabi vs warf:<br>0.75 [0.52-1.07]<br>Riva vs warf:<br>0.84 [0.59-1.19]<br>Riva vs dabi:<br>1.12 [0.77-1.63]<br>***High multimorbidity:***<br>**CHA_2DS_2-VASc ≥6:**<br>Dabi vs warf:<br>0.87 [0.65-1.16]<br>Riva vs warf:<br>0.81 [0.59-1.10]<br>Riva vs dabi:<br>0.93 [0.68-1.27]<br>**HAS-BLED ≥3:**<br>Dabi vs warf:<br>1.26 [0.84-1.89]<br>Riva vs warf:<br>1.15 [0.76-1.75]<br>Riva vs dabi:<br>0.91 [0.61-1.35]<br>**Gagne ≥5:**<br>Dabi vs warf:<br>0.99 [0.70-1.38]<br>Riva vs warf:<br>0.74 [0.51-1.07]<br>Riva vs dabi:<br>0.75 [0.52-1.09]	0.91 [0.74-1.11]<br>Riva vs warf:<br>1.20 [0.98-1.45]<br>Riva vs dabi:<br>*1.32 [1.08-1.61]*<br>**Gagne 3-4:**<br>Dabi vs warf:<br>0.85 [0.68-1.06]<br>Riva vs warf:<br>1.09 [0.88-1.34]<br>Riva vs dabi:<br>*1.28 [1.03-1.59]*<br>***High multimorbidity:***<br>**CHA_2DS_2-VASc ≥6:**<br>Dabi vs warf:<br>0.91 [0.74-1.11]<br>Riva vs warf:<br>1.16 [0.96-1.41]<br>Riva vs dabi:<br>*1.28 [1.05-1.56]*<br>**HAS-BLED ≥3:**<br>Dabi 150 vs warf:<br>0.83 [0.64-1.07]<br>Riva vs warf:<br>1.09 [0.85-1.39]<br>Riva vs dabi:<br>*1.31 [1.02-1.69]*<br>**Gagne ≥5:**<br>Dabi vs warf:<br>0.93 [0.76-1.14]<br>Riva vs warf:<br>1.15 [0.95-1.40]<br>Riva vs dabi:<br>*1.24 [1.01-1.51]*		0.98 [0.79-1.22]<br>Riva vs warf:<br>*1.26 [1.02-1.56]*<br>Riva vs dabi:<br>*1.28 [1.04-1.59]*<br>**Gagne 3-4:**<br>Dabi vs warf:<br>1.01 [0.79-1.28]<br>Riva vs warf:<br>*1.25 [1.00-1.58]*<br>Riva vs dabi:<br>1.25 [0.99-1.57]<br>***High multimorbidity:***<br>**CHA_2DS_2-VASc ≥6:**<br>Dabi vs warf:<br>1.08 [0.87-1.35]<br>Riva vs warf:<br>*1.28 [1.04-1.59]*<br>Riva vs dabi:<br>1.18 [0.96-1.46]<br>**HAS-BLED ≥3:**<br>Dabi vs warf:<br>1.07 [0.80-1.42]<br>Riva vs warf:<br>*1.36 [1.04-1.79]*<br>Riva vs dabi:<br>1.28 [0.98-1.67]<br>**Gagne ≥5:**<br>Dabi vs warf:<br>1.11 [0.89-1.39]<br>Riva vs warf:<br>*1.28 [1.03-1.60]*<br>Riva vs dabi:<br>1.16 [0.94-1.43]	**0.75 [0.61-0.92]**<br>Riva vs warf:<br>**0.72 [0.58-0.88]**<br>Riva vs dabi:<br>0.95 [0.76-1.19]<br>**Gagne 3-4:**<br>Dabi vs warf:<br>0.79 [0.61-1.03]<br>Riva vs warf:<br>0.84 [0.65-1.08]<br>Riva vs dabi:<br>1.06 [0.81-1.39]<br>***High multimorbidity:***<br>**CHA_2DS_2-VASc ≥6:**<br>Dabi vs warf:<br>**0.77 [0.62-0.96]**<br>Riva vs warf:<br>**0.70 [0.56-0.87]**<br>Riva vs dabi:<br>0.91 [0.72-1.15]<br>**HAS-BLED ≥3:**<br>Dabi vs warf:<br>**0.70 [0.51-0.95]**<br>Riva vs warf:<br>0.80 [0.60-1.09]<br>Riva vs dabi:<br>1.15 [0.83-1.60]<br>**Gagne ≥5:**<br>Dabi vs warf:<br>**0.71 [0.59-0.86]**<br>Riva vs warf:<br>**0.74 [0.62-0.89]**<br>Riva vs dabi:<br>1.04 [0.85-1.27]
Hernandez et al. 2018[39]	Observational retrospective nationwide cohort study (USA)	AF patients from administrative healthcare claims database, categorized according to CHA_2DS_2-VASc score ≤3 (low multimorbidity), 4-5 (moderate multimorbidity) and ≥6	**CHA_2DS_2-VASc 4-5:** 553 dabi, 2189 riva, 1028 api, 5106 warf;<br>**CHA_2DS_2-VASc ≥6:**	**CHA_2DS_2-VASc 4-5:** 76.8y +/- 7.9 dabi, 77.4y +/-7.8 riva, 77.6y +/- 8.3 api, 76.6y +/- 9.5 warf;<br>**CHA_2DS_2-VASc ≥6:**	**CHA_2DS_2-VASc 4-5:** 307 days +/- 200 dabi, 253 days +/- 183 riva, 186 days +/- 139 api,	***Stroke/SE and mortality:***<br>**CHA_2DS_2-VASc 4-5:**Dabi vs warf:<br>**0.68 [0.54-0.86]**<br>Riva vs warf:<br>**0.73 [0.64-0.83]**<br>Api vs warf:	***Any bleeding:***<br>**CHA_2DS_2-VASc 4-5:**<br>Dabi vs warf:<br>0.91 [0.74-1.12]<br>Riva vs warf:<br>*1.19 [1.06-1.33]*<br>Api vs warf:	NR	**CHA_2DS_2-VASc 4-5:**<br>Dabi vs warf:<br>0.95 [0.67-1.34]<br>Riva vs warf:<br>*1.37 [1.14-1.64]*<br>Api vs warf:<br>0.76 [0.54-1.07]	**CHA_2DS_2-VASc 4-5:**<br>Dabi vs warf:<br>**0.28 [0.12-0.68]**<br>Riva vs warf:<br>0.87 [0.66-1.17]<br>Api vs warf:<br>**0.52 [0.31-0.88]**

		(high multimorbidity). NOAC (dabi, riva, api) vs warf. OAC-naïve.	332 dabi, 1441 riva, 721 api, 4222 warf	80.6y +/- 7.2 dabi, 81.1y +/- 7.2 riva, 82.2y +/- 6.6 api, 80.8y +/- 7.9 warf	273 days +/- 186 warf;<br>**CHA$_2$DS$_2$-VASc ≥6:**<br>279 days +/- 192 dabi, 253 days +/- 180 riva, 176 days +/- 138 api, 262 days +/- 183 warf	**0.71 [0.58-0.86]**<br>**CHA$_2$DS$_2$-VASc ≥6:**<br>Dabi vs warf:<br>**0.66 [0.53-0.83]**<br>Riva vs warf:<br>**0.78 [0.69-0.87]**<br>Api vs warf:<br>0.86 [0.74-1.01]<br>***Ischemic stroke:***<br>**CHA$_2$DS$_2$-VASc 4-5:**<br>Dabi vs warf:<br>1.07 [0.83-1.39]<br>Riva vs warf:<br>0.88 [0.74-1.04]<br>Api vs warf:<br>1.03 [0.81-1.31]<br>**CHA$_2$DS$_2$-VASc ≥6:**<br>Dabi vs warf:<br>0.77 [0.59-0.99]<br>Riva vs warf:<br>**0.80 [0.70-0.92]**<br>Api vs warf:<br>1.01 [0.84-1.22]<br>***Non-central SE:***<br>**CHA$_2$DS$_2$-VASc 4-5:**<br>Dabi vs warf:<br>**0.43 [0.28-0.68]**<br>Riva vs warf:<br>**0.50 [0.39-0.63]**<br>Api vs warf:<br>**0.33 [0.22-0.51]**<br>**CHA$_2$DS$_2$-VASc ≥6:**<br>Dabi vs warf:<br>**0.53 [0.34-0.84]**<br>Riva vs warf:<br>**0.69 [0.56-0.84]**<br>Api vs warf:<br>**0.57 [0.41-0.79]**	0.83 [0.68-1.00]<br>**CHA$_2$DS$_2$-VASc ≥6:**<br>Dabi vs warf:<br>1.04 [0.82-1.32]<br>Riva vs warf:<br>1.08 [0.95-1.23]<br>Api vs warf:<br>0.78 [0.63-0.96] (non-significant at alpha level of 0.016 after application of Bonferroni correction)		**CHA$_2$DS$_2$-VASc ≥6:**<br>Dabi vs warf:<br>1.19 [0.83-1.71]<br>Riva vs warf:<br>1.27 [1.04-1.54] (non-significant at alpha level of 0.016 after application of Bonferroni correction)<br>Api vs warf:<br>0.75 [0.52-1.07]	**CHA$_2$DS$_2$-VASc ≥6:**<br>Dabi vs warf:<br>0.72 [0.39-1.33]<br>Riva vs warf:<br>0.86 [0.64-1.17]<br>Api vs warf:<br>1.14 [0.78-1.67]
Hohmann et al. 2019[19]	Observational retrospective	AF patients from administrative healthcare claims database,	**Overall:**	**CCI ≥4:**<br>77.3y +/- 8.6	706 days +/- 378 NOAC;	**CCI ≥4:**<br>NOAC vs phen:<br>0.94 [0.79-1.13]	***Major extracranial bleeding:***	**CCI ≥4:**<br>NOAC vs phen:<br>**0.52 [0.39-0.69]**	**CCI ≥4:**<br>NOAC vs phen:<br>1.00 [0.84-1.19]	NR

	nationwide cohort study (Germany)	categorized according to Charlson Comorbidity score (CCI) of <4 and ≥4 (multimorbidity). NOAC (12.7% dabi, 56.2% riva, 31.1% api) vs phen, OAC-naïve.	42562 NOAC, 27939 phen. **CCI ≥4:** 26410 (NOAC and phen)		856 days +/- 395 phen (overall, NR for CCI ≥4)		**CCI ≥4:**<br>NOAC vs phen:<br>**0.70 [0.56-0.87]**<br>Dabi vs phen:<br>**0.48 [0.34-0.67]**<br>Riva vs phen:<br>0.84 [0.66-1.07]<br>Api vs phen:<br>0.79 [0.43-1.26]		Dabi vs phen:<br>0.84 [0.57-1.25]<br>Riva vs phen:<br>*1.23 [1.01-1.49]*<br>Api vs phen:<br>**0.71 [0.55-0.91]**	
Wong et al. 2020[(29)]	Observational retrospective nationwide cohort study (USA)	AF patients included from NCDR PINNACLE national ambulatory registry matched to administrative healthcare claims database, **HAS-BLED score** <4 and **≥4.** OAC-naïve and –experienced.	32737 dabi, 40994 riva, 17971 api, 177318 warf, 154430 no OAC (overall, NR in HAS-BLED ≥4 group)	75.5y +/- 7.3 dabi; 75.6y +/- 7.3 riva; 76.5y +/- 7.4 api; 77.3y +/- 7.5 warf; 77.1y +/- 8.5 no OAC (overall, no separate results in HAS-BLED ≥4 group)	1.4 years +/- 0.6 (overall, no separate results in HAS-BLED ≥4 group)	NR	**HAS-BLED ≥4:**<br>NOAC vs warf:<br>**0.87 [0.80-0.95]**<br>Dabi vs warf:<br>**0.75 [0.66-0.86]**<br>Riva vs warf:<br>1.01 [0.90-1.14]<br>Api vs warf:<br>**0.80 [0.65-0.99]**	**HAS-BLED ≥4:**<br>NOAC vs warf:<br>**0.74 [0.56-0.99]**<br>Dabi vs warf:<br>0.74 [0.49-1.11]<br>Riva vs warf:<br>0.80 [0.55-1.18]<br>Api vs warf:<br>0.58 [0.27-1.23]	**HAS-BLED ≥4:**<br>NOAC vs warf:<br>1.02 [0.89-1.16]<br>Dabi vs warf:<br>**0.76 [0.61-0.94]**<br>Riva vs warf:<br>*1.34 [1.14-1.58]*<br>Api vs warf:<br>0.81 [0.58-1.12]	NR

eTable 3: Overview of included studies investigating the impact of multimorbidity on the effectiveness and safety of oral anticoagulants.

Bold: significantly lower risk; *Italic*: significantly higher risk

AF: atrial fibrillation; Api: apixaban; CCI: Charlson Comorbidity Score; CI: confidence interval; Dabi 110: dabigatran 110 mg (reduced dose); Dabi 150: dabigatran 150 mg (standard dose); Edo 30: edoxaban 30 mg (reduced dose); Edo 60: edoxaban 60 mg (standard dose); HR: hazard ratio; IQR: interquartile range; NOAC: non-vitamin K antagonist oral anticoagulant; NR: not reported; OAC: oral anticoagulant; Phen: phenprocoumon; PSM: propensity score matching; RCT: randomized controlled trial; Riva 20: rivaroxaban 20 mg; Riva 15: rivaroxaban 15 mg (reduced dose); SD: standard deviation; SE: systemic embolism; Stroke/SE: stroke/systemic embolism; USA: United States of America; Vs: versus; Warf: warfarin; y: year

1.4 eTable 4: Polypharmacy

Author	Study design	Study cohort	n	Mean/median age (years +/- SD; [IQR])	Mean/median follow-up (+/- SD; [IQR])	Stroke/SE (HR [95% CI])	Major bleeding (HR [95% CI])	Intracranial bleeding (HR [95% CI])	Gastrointestinal bleeding (HR [95% CI])	All-cause mortality (HR [95% CI])
Jaspers Focks et al. 2016[40]	Phase III RCT (worldwide)	AF patients included in the ARISTOTLE trial (api vs warf), categorized according to 0-5, **6-8 and ≥9 drugs used.** Industry-sponsored.	**6-8 drugs:** 3320 api (288 api 2.5), 3182 warf; **≥9 drugs:** 2376 api (290 api 2.5), 2380 warf	**6-8 drugs:** 69y +/- 10; **≥9 drugs:** 71y +/- 9	1.8 years [1.3-2.3] (overall, no separate results in polypharmacy groups)	**6-8 drugs:** Api vs warf: 0.76 [0.57-1.03] **≥9 drugs:** Api vs warf: 0.76 [0.54-1.07]	***Major bleeding:*** **6-8 drugs:** Api vs warf: **0.72 [0.56-0.91]** **≥9 drugs:** Api vs warf: 0.84 [0.67-1.06] ***CRNMB:*** **6-8 drugs:** Api vs warf: **0.64 [0.50-0.81]** **≥9 drugs:** Api vs warf: **0.75 [0.59-0.96]**	**6-8 drugs:** Api vs warf: **0.43 [0.25-0.74]** **≥9 drugs:** Api vs warf: **0.29 [0.15-0.56]**	**6-8 drugs:** Api vs warf: 0.81 [0.52-1.26] **≥9 drugs:** Api vs warf: 1.14 [0.75-1.72]	**6-8 drugs:** Api vs warf: 0.89 [0.74-1.06] **≥9 drugs:** Api vs warf: 0.94 [0.77-1.14]
Piccini et al. 2016[41]	Phase III RCT (worldwide)	AF patients included in the ROCKET AF trial (riva vs warf), categorized according to 0-4, **5-9 and ≥10 drugs used.** Industry-sponsored.	**5-9 drugs:** 3627 riva, 3624 warf; **≥10 drugs:** 936 riva, 926 warf	**5-9 drugs:** 73y [66-78]; **≥10 drugs:** 75y [68-79]	707 days (overall, no separate results in polypharmacy groups)	**5-9 drugs:** Riva vs warf: 2.18%/y riva, 2.49%/y warf; **≥10 drugs:** Riva vs warf: 1.86%/y riva, 2.16%/y warf (incidence, no risk estimates)	**5-9 drugs:** Riva vs warf: *HR 1.23 [1.01-1.49]* **≥10 drugs:** Riva vs warf: HR 1.17 [0.87-1.56]	**5-9 drugs:** Riva vs warf: 0.61%/y riva, 0.79%/y warf; **≥10 drugs:** Riva vs warf: 0.67%/y riva, 0.80%/y warf (incidence, no risk estimates)	NR	**5-9 drugs:** Riva vs warf: 4.89%/y riva, 5.28%/y warf; **≥10 drugs:** Riva vs warf: 5.93%/y riva, 6.89%/y warf (incidence, no risk estimates)
Kim et al. 2019[42]	Meta-analysis	Pooled data of 2 phase III RCTs (ARISTOTLE and ROCKET-AF trial) in AF, categorized according to <5 and **≥5 drugs used.** NOAC (api, riva) vs warf.	**≥5 drugs:** 10286 NOAC (5696 api, 4590 riva), 10112 warf	NR	NR	**≥5 drugs:** NOAC vs warf: **RR 0.82 [0.71-0.96]**	**≥5 drugs:** NOAC vs warf: RR 0.95 [0.65-1.39]	**≥5 drugs:** NOAC vs warf: RR 0.53 [0.26-1.11]	NR	**≥5 drugs:** NOAC vs warf: **RR 0.91 [0.83-0.99]**
Harskamp et al. 2019[43]	Meta-analysis	Pooled data of 2 phase III RCTs (ARISTOTLE and ROCKET-AF trial) in AF, categorized	**≥5 drugs:** 23095 overall: 7022 api, 4590 riva,	NR	1.9 years	***Stroke/SE:*** **≥5 drugs:** NOAC vs warf: **RR 0.84 [0.74-0.94]**	**≥5 drugs:** NOAC vs warf: RR 0.94 [0.64-1.24] Riva vs warf: RR 1.16 [0.99-1.35]	**≥5 drugs:** NOAC vs warf: **RR 0.51 [0.38-0.70]**	NR	**≥5 drugs:** NOAC vs warf: **RR 0.91 [0.84-0.98]**

		according to <5 and **≥5 drugs used.** NOAC (api, riva) vs warf.	11483 warf			***Ischemic stroke:*** NOAC vs warf: RR 0.90 [0.70-1.10] Riva vs warf: RR 0.88 [0.72-1.08] Api vs warf: **RR 0.76 [0.62-0.93]**	Api vs warf: **RR 0.74 [0.63-0.86]** ***CRNMB:*** NOAC vs warf: RR 0.85 [0.56-1.14] Riva vs warf: RR 1.01 [0.92-1.10] Api vs warf: **RR 0.69 [0.59-0.81]**	Riva vs warf: RR 0.77 [0.52-1.14] Api vs warf: **RR 0.35 [0.24-0.52]**		Riva vs warf: RR 0.92 [0.81-1.04] Api vs warf: RR 0.91 [0.81-1.02]
Hohmann et al. 2019[19]	Observational retrospective nationwide cohort study (Germany)	AF patients from administrative healthcare claims database, categorized according to <7 and **≥7 drugs** used. NOAC (12.7% dabi, 56.2% riva, 31.1% api) vs phen, OAC-naïve.	**Overall:** 42562 NOAC, 27939 phen **≥7 drugs:** 33238 (NOAC and phen)	**≥7 drugs:** 75.9y +/- 9.4	706 days +/- 378 NOAC; 856 days +/- 395 phen (overall, NR for ≥7 drugs used)	**≥7 drugs:** NOAC vs phen: 0.89 [0.75-1.05]	***Major extracranial bleeding:*** **≥7 drugs:** NOAC vs phen: **0.72 [0.59-0.87]** Dabi vs phen: **0.46 [0.33-0.63]** Riva vs phen: 0.90 [0.73-1.12] Api vs phen: 0.63 [0.40-1.09]	**≥7 drugs:** NOAC vs phen: **0.54 [0.42-0.70]**	**≥7 drugs:** NOAC vs phen: 1.01 [0.86-1.20] Dabi vs phen: 0.88 [0.61-1.27] Riva vs phen: *1.30 [1.09-1.56]* Api vs phen: **0.62 [0.48-0.80]**	NR
Martinez et al. 2019[44]	Observational retrospective nationwide cohort study (USA)	AF patients from administrative healthcare claims database, categorized according to **≥5 and ≥10 drugs** used. Riva (24.1% and 30.4% reduced dose in respective subgroups) vs warf, OAC-naïve. Industry-sponsored.	**≥5 drugs:** 13981 riva, 13981 warf; **≥10 drugs:** 1765 riva, 1765 warf (after 1:1 PSM)	**≥5 drugs:** 71y [62-79] riva, 72y [63-80] warf; **≥10 drugs:** 71y [63-79] riva, 72y [64-80] warf	**≥5 drugs:** 1.7 year [0.7-3.0] **≥10 drugs:** 1.4 years [0.6-2.7]	**≥5 drugs:** Riva vs warf: **0.66 [0.50-0.88]** **≥10 drugs:** Riva vs warf: 0.44 [0.17-1.12]	**≥5 drugs:** Riva vs warf: 1.08 [0.92-1.28] **≥10 drugs:** Riva vs warf: 1.07 [0.73-1.58]	NR	NR	NR

eTable 4: Overview of included studies investigating the impact of polypharmacy on the effectiveness and safety of oral anticoagulants.

Bold: significantly lower risk; *Italic*: significantly higher risk

AF: atrial fibrillation; Api: apixaban; Api 2.5: apixaban 2.5 mg (reduced dose); CI: confidence interval; CRNMB: clinically relevant non-major bleeding; Dabi: dabigatran; HR: hazard ratio; IQR: interquartile range; NOAC: non-vitamin K antagonist oral anticoagulant; NR: not reported; OAC: oral anticoagulant; Phen: phenprocoumon; PSM: propensity score matching; RCT: randomized controlled trial; Riva: rivaroxaban; RR: relative risk; SD: standard deviation; Stroke/SE: stroke/systemic embolism; USA: United States of America; Vs: versus; Warf: warfarin; y: year

1.5 eTable 5: High falling risk

Author	Study design	Study cohort	n	Mean/median age (years +/- SD; [IQR])	Mean/median follow-up (+/- SD; [IQR])	Stroke/SE (HR [95% CI])	Major bleeding (HR [95% CI])	Intracranial bleeding (HR [95% CI])	Gastrointestinal bleeding (HR [95% CI])	All-cause mortality (HR [95% CI])
Steffel et al. 2016[45]	Phase III RCT (worldwide)	AF patients included in the ENGAGE AF-TIMI 48 trial at high risk of falling (based on presence of any of 8 criteria such as prior falls, lower extremity weakness, poor balance etc.). High-dose edo regimen (60/30 mg) vs warf. Industry-sponsored.	**High falling risk:** 310 high-dose edo regimen, 307 warf	**High falling risk:** 77y [72-82]	2.8 years (overall, no separate results in high falling risk group)	Edo 60/30 vs warf: 0.96 [0.53-1.75]	***Major bleeding*** Edo 60/30 vs warf: 0.96 [0.59-1.56] ***Major bleeding or CRNMB:*** Edo 60/30 vs warf: 0.83 [0.64-1.08] ***Life-threatening bleeding*** Edo 60/30 vs warf: **0.32 [0.10-0.98]**	Edo 60/30 vs warf: **0.16 [0.04-0.71]**	Edo 60/30 vs warf: 1.98 [0.88-4.46]	Edo 60/30 vs warf: 0.91 [0.64-1.29]
Rao et al. 2018[46]	Phase III RCT (worldwide)	AF patients included in the ARISTOTLE trial (api vs warf) with ≥1 fall within one year. Industry-sponsored.	**High falling risk:** 386 api, 367 warf	**High falling risk:** 75y [67-79]	1.8 years [1.4-2.3] (overall, no separate results in high falling risk group)	Api vs warf: 0.88 [0.40-1.93]	***Major bleeding*** Api vs warf: 0.81 [0.48-1.36] ***Major bleeding or CRNMB:*** Api vs warf: 0.95 [0.65-1.41] ***Any bleeding*** Api vs warf: **0.65 [0.52-0.81]**	Api vs warf: **0.19 [0.04-0.88]**	NR	Api vs warf: 0.96 [0.63-1.44]

eTable 5: Overview of included studies investigating the impact of high falling risk on the effectiveness and safety of oral anticoagulants.

Bold: significantly lower risk; *Italic*: significantly higher risk

AF: atrial fibrillation; Api: apixaban; CI: confidence interval; CRNMB: clinically relevant non-major bleeding; Edo 30: edoxaban 30 mg (reduced dose); Edo 60: edoxaban 60 mg (standard dose); HR: hazard ratio; IQR: interquartile range; NR: not reported; RCT: randomized controlled trial; SD: standard deviation; Stroke/SE: stroke/systemic embolism; Vs: versus; Warf: warfarin; y: year

1.6 eTable 6: Frailty

Author	Study design	Study cohort	n	Mean/median age (years +/- SD; [IQR])	Mean/median follow-up (+/- SD; [IQR])	Stroke/SE (HR [95% CI])	Major bleeding (HR [95% CI])	Intracranial bleeding (HR [95% CI])	Gastrointestinal bleeding (HR [95% CI])	All-cause mortality (HR [95% CI])
Hohmann et al. 2019[19]	Observational retrospective nationwide cohort study (Germany)	Frail AF patients from administrative healthcare claims database (based on Johns Hopkins Claims-based Frailty Indicator scoring algorithm), NOAC (12.7% dabi, 56.2% riva, 31.1% api) vs phen, OAC-naïve.	**Frail:**<br>36267 (NOAC and phen)	**Frail:**<br>76.7y +/- 9.5	706 days +/- 378 NOAC;<br>856 days +/- 395 phen<br>(overall, NR for frailty)	**Frailty:**<br>NOAC vs phen:<br>0.91 [0.77-1.07]	***Major extracranial bleeding:***<br>**Frailty:**<br>NOAC vs phen:<br>**0.73 [0.60-0.89]**<br>Dabi vs phen:<br>**0.53 [0.39-0.73]**<br>Riva vs phen:<br>0.90 [0.72-1.13]<br>Api vs phen:<br>**0.54 [0.32-0.89]**	**Frailty:**<br>NOAC vs phen:<br>**0.52 [0.41-0.67]**	**Frailty:**<br>NOAC vs phen:<br>1.09 [0.93-1.28]<br>Dabi vs phen:<br>1.00 [0.71-1.40]<br>Riva vs phen:<br>*1.38 [1.16-1.64]*<br>Api vs phen:<br>**0.68 [0.53-0.87]**	NR
Martinez et al. 2018[47]	Observational retrospective nationwide cohort study (USA)	Frail AF patients from administrative healthcare claims database (based on Johns Hopkins Claims-based Frailty Indicator scoring algorithm), NOAC (dabi, riva, api) vs warf, OAC-naïve. Industry-sponsored.	**Frail:**<br>3x 1:1 PSM cohorts:<br>1350 dabi-warf,<br>2635 riva-warf,<br>1392 api-warf	**Dabi-warf:**<br>85y [82-88] dabi, 86y [82-89] warf;<br>**Riva-warf:**<br>85y [82-89] riva, 86y [82-89] warf;<br>**Api-warf:**<br>86y [83-89] api, 86y [83-89] warf<br>(after 1:1 PSM)	**Dabi-warf:**<br>1.8 year [0.8-2.0];<br>**Riva-warf:**<br>1.4 year [0.7-2.0];<br>**Api-warf:**<br>0.9 year [0.4-1.6]	***After 1y follow-up:***<br>Dabi vs warf:<br>0.96 [0.55-1.66]<br>Riva vs warf:<br>0.79 [0.52-1.20]<br>Api vs warf:<br>0.71 [0.37-1.35]<br>***After 2y follow-up:***<br>Dabi vs warf:<br>0.94 [0.60-1.45]<br>Riva vs warf:<br>**0.68 [0.49-0.95]**<br>Api vs warf:<br>0.78 [0.46-1.35]	***1y follow-up:***<br>Dabi vs warf:<br>0.92 [0.62-1.37]<br>Riva vs warf:<br>1.06 [0.81-1.39]<br>Api vs warf:<br>**0.61 [0.39-0.93]**<br>***2y follow-up:***<br>Dabi vs warf:<br>0.87 [0.63-1.19]<br>Riva vs warf:<br>1.04 [0.81-1.32]<br>Api vs warf:<br>0.72 [0.49-1.06]	***1y follow-up:***<br>Dabi vs warf:<br>**0.18 [0.04-0.81]**<br>Riva vs warf:<br>**0.37 [0.15-0.94]**<br>Api vs warf:<br>0.97 [0.28-3.33]<br>***2y follow-up:***<br>Dabi vs warf:<br>0.14 [0.02-1.11]<br>Riva vs warf:<br>0.49 [0.23-1.04]<br>Api vs warf:<br>0.97 [0.28-3.33]	***1y follow-up:***<br>Dabi vs warf:<br>1.09 [0.69-1.72]<br>Riva vs warf:<br>*1.39 [1.01-1.90]*<br>Api vs warf:<br>0.62 [0.39-1.08]<br>***2y follow-up:***<br>Dabi vs warf:<br>0.94 [0.66-1.35]<br>Riva vs warf:<br>1.27 [0.96-1.68]<br>Api vs warf:<br>0.76 [0.48-1.21]	NR

eTable 6: Overview of included studies investigating the impact of frailty on the effectiveness and safety of oral anticoagulants.

Bold: significantly lower risk; *Italic*: significantly higher risk

AF: atrial fibrillation; Api: apixaban; CI: confidence interval; Dabi: dabigatran; HR: hazard ratio; IQR: interquartile range; NOAC: non-vitamin K antagonist oral anticoagulant; NR: not reported; OAC: oral anticoagulant; Phen: phenprocoumon; PSM: propensity score matching; Riva: rivaroxaban; SD: standard deviation; Stroke/SE: stroke/systemic embolism; USA: United States of America; Vs: versus; Warf: warfarin; y: year

1.7 eTable 7: Baseline dementia

Author	Study design	Study cohort	n	Mean/median age (years +/- SD; [IQR])	Mean/median follow-up (+/- SD; [IQR])	Stroke/SE (HR [95% CI])	Major bleeding (HR [95% CI])	Intracranial bleeding (HR [95% CI])	Gastrointestinal bleeding (HR [95% CI])	All-cause mortality (HR [95% CI])
Subic et al. 2018[48]	Observational retrospective nationwide cohort study (Sweden)	AF patients with new dementia diagnosis, included in the Swedish Dementia Registry from 2007 to 2014, using warf, antiplatelets (AP) or no OAC/AP	**Dement AF:** 2143 warf, 2975 antiplatelet, 2978 no OAC/AP	80.8y +/- 5.8 warf, 83.3y +/- 6.2 antiplatelet, 82.3y +/- 6.5 no OAC/AP	636 days [805]	***Ischemic stroke:*** Warf vs no OAC/AP: **0.76 [0.59-0.98]**	***Any bleeding:*** Warf vs no OAC/AP: 1.08 [0.87-1.35]	***Non-traumatic intracranial bleeding:*** Warf vs no OAC/AP: 1.47 [0.91-2.37]	NR	Warf vs no OAC/AP: **0.84 [0.59-0.98]**
Orkaby et al. 2017[49]	Observational retrospective nationwide cohort study (USA)	AF patients with new dementia diagnosis in 2007-2008 after ≥6 months warf use in 2006, included in the Veterans Affairs database, warf continuation vs discontinuation after dementia diagnosis.	**Dement AF overall:** 2572; 1:2 PSM cohort: 405 warf continuers, 810 warf discontinuers	**Overall:** 79.5y +/- 6.0	2.2 years (maximum duration up to 4 years)	Warf continuation vs discontinuation: **0.74 [0.54-0.996], p-value 0.047**	Warf continuation vs discontinuation: 0.78 [0.61-1.01]	NR	NR	Warf continuation vs discontinuation: **0.72 [0.60-0.87]**
Fanning et al. 2020[50]	Observational retrospective nationwide cohort study (UK)	AF patients with baseline dementia, newly started on OAC (OAC-naïve), included in the THIN database from 2011 to 2017, propensity score-adjusted poisson regression	**Dement AF:** 1013 NOAC (77 dabi, 503 riva, 428 api, 5 edo; 47% reduced dose), 1386 warf	84y [79-88] NOAC, 81y [77-86] warf	1978 PY	***Stroke/SE:*** NOAC vs warf: IRR: 0.91 [0.67-1.25] ARD: -4.0/1000 PY [-5.4, 11.5] ***Ischemic stroke:*** NOAC vs warf: IRR: 1.16 [0.78-1.73] ARD: 4.0/1000 PY [-5.5, 18.1]	***Other major bleeding:*** NOAC vs warf: IRR: 0.87 [0.59-1.28] ARD: -4.1/1000 PY [-12.6, 8.4]	NOAC vs warf: IRR: **0.27 [0.08-0.86]** ARD: **-5.2/1000 PY [-6.5, -1.0]**	***Gastro-intestinal bleeding:*** NOAC vs warf: IRR: *2.11 [1.30-3.42]* ARD: *14.8/1000 PY [4.0-32.4]*	NOAC vs warf: IRR: *2.06 [1.60-2.65]* ARD: *53.0/1000 PY [30.2, 82.2]*

eTable 7: Overview of included studies investigating the impact of baseline dementia on the effectiveness and safety of oral anticoagulants.
Bold: significantly lower risk; *Italic*: significantly higher risk
AF: atrial fibrillation; AP: antiplatelet; Api: apixaban; ARD: absolute risk difference; CI: confidence interval; Dabi: dabigatran; Edo: edoxaban; HR: hazard ratio; IQR: interquartile range; IRR: Incidence rate ratio; NOAC: non-vitamin K antagonist oral anticoagulant; NR: not reported; OAC: oral anticoagulant; PY: person-year; Riva: rivaroxaban; SD: standard deviation; Stroke/SE: stroke/systemic embolism; UK: United Kingdom; USA: United States of America; Vs: versus; Warf: warfarin; y: year

1.8 eTable 8: Assessment of bias within studies

A)

Reference: Eikelboom et al. 2011[1]					
**Criteria**		**Yes (2)**	**Partial (1)**	**No (0)**	**N/A**
**1**	Question / objective sufficiently described?	2			
**2**	Study design evident and appropriate?	2			
**3**	Method of subject/comparison group selection or source of information/input variables described and appropriate?	2			
**4**	Subject and comparison group (if applicable) characteristics sufficiently described?		1 (randomized study with comparable baseline characteristics reported for total included cohort using NOAC or VKA, but not for subset of AF patients ≥75 years)		
**5**	If interventional and random allocation was possible, was it reported?	2			
**6**	If interventional and blinding of investigators was possible, was it reported?	2			
**7**	If interventional and blinding of subjects was possible, was it reported?	2			
**8**	Outcome and (if applicable) exposure measure(s) well defined and robust to measurement / misclassification bias? Means of assessment reported?	2			
**9**	Sample size appropriate?	2			
**10**	Analytic methods described/justified and appropriate?	2			
**11**	Some estimate of variance is reported for the main results?	2			
**12**	Controlling for confounding?		1 (randomized study with comparable baseline characteristics reported for total included cohort using NOAC or VKA, but not for subset of AF patients ≥75 years; no adjustment for potential confounders)		
**13**	Results reported in sufficient detail?	2			
**14**	Conclusion supported by the results?	2			
Total score: 26/28 (92.6%)					

B)

Reference: Lauw et al. 2017[2]					
**Criteria**		**Yes (2)**	**Partial (1)**	**No (0)**	**N/A**
**1**	Question / objective sufficiently described?	2			
**2**	Study design evident and appropriate?	2			
**3**	Method of subject/comparison group selection or source of information/input variables described and appropriate?	2			
**4**	Subject and comparison group (if applicable) characteristics sufficiently described?		1 (randomized study with baseline characteristics reported for included anticoagulated AF cohort aged <75, 75-80, 80-85 and ≥ 85 years, but not specifically compared between NOAC and VKA)		
**5**	If interventional and random allocation was possible, was it reported?	2			
**6**	If interventional and blinding of investigators was possible, was it reported?	2			
**7**	If interventional and blinding of subjects was possible, was it reported?	2			
**8**	Outcome and (if applicable) exposure measure(s) well defined and robust to measurement / misclassification bias? Means of assessment reported?	2			
**9**	Sample size appropriate?	2			
**10**	Analytic methods described/justified and appropriate?	2			
**11**	Some estimate of variance is reported for the main results?	2			
**12**	Controlling for confounding?		1 (randomized study with baseline characteristics reported for included anticoagulated AF cohort aged <75, 75-80, 80-85 and ≥ 85 years, but not specifically compared between NOAC and VKA; no adjustment for potential confounders)		
**13**	Results reported in sufficient detail?	2			
**14**	Conclusion supported by the results?	2			
Total score: 26/28 (92.6%)					

C)

Reference: Halperin et al. 2014[3]					
**Criteria**		**Yes (2)**	**Partial (1)**	**No (0)**	**N/A**
**1**	Question / objective sufficiently described?	2			
**2**	Study design evident and appropriate?	2			
**3**	Method of subject/comparison group selection or source of information/input variables described and appropriate?	2			
**4**	Subject and comparison group (if applicable) characteristics sufficiently described?	2			
**5**	If interventional and random allocation was possible, was it reported?	2			
**6**	If interventional and blinding of investigators was possible, was it reported?	2			
**7**	If interventional and blinding of subjects was possible, was it reported?	2			
**8**	Outcome and (if applicable) exposure measure(s) well defined and robust to measurement / misclassification bias? Means of assessment reported?	2			
**9**	Sample size appropriate?	2			
**10**	Analytic methods described/justified and appropriate?	2			
**11**	Some estimate of variance is reported for the main results?		1 (results on stroke/SE, major bleeding and intracranial bleeding risk reported in sufficient detail, results on gastrointestinal bleeding risk only reported as event rates without estimate of variance)		
**12**	Controlling for confounding?	2			
**13**	Results reported in sufficient detail?		1 (results on stroke/SE, major bleeding and intracranial bleeding risk reported in sufficient detail, results on gastrointestinal bleeding risk only reported as event rates without estimate of variance)		
**14**	Conclusion supported by the results?		1 (conclusion that efficacy and safety of rivaroxaban relative to warfarin did not differ with age, while the risk of major or clinically relevant non-major bleeding and gastrointestinal bleeding was significantly increased with rivaroxaban at higher age)		
Total score: 25/28 (89.3%)					

D)

Reference: Hori et al. 2014[4]					
**Criteria**		**Yes (2)**	**Partial (1)**	**No (0)**	**N/A**
**1**	Question / objective sufficiently described?	2			
**2**	Study design evident and appropriate?	2			
**3**	Method of subject/comparison group selection or source of information/input variables described and appropriate?	2			
**4**	Subject and comparison group (if applicable) characteristics sufficiently described?	2			
**5**	If interventional and random allocation was possible, was it reported?	2			
**6**	If interventional and blinding of investigators was possible, was it reported?	2			
**7**	If interventional and blinding of subjects was possible, was it reported?	2			
**8**	Outcome and (if applicable) exposure measure(s) well defined and robust to measurement / misclassification bias? Means of assessment reported?	2			
**9**	Sample size appropriate?		1 (limited sample sizes for subgroup of patients ≥75 years old: 252 rivaroxaban, 246 warfarin)		
**10**	Analytic methods described/justified and appropriate?	2			
**11**	Some estimate of variance is reported for the main results?	2			
**12**	Controlling for confounding?	2			
**13**	Results reported in sufficient detail?	2			
**14**	Conclusion supported by the results?	2			
Total score: 27/28 (96.4%)					

E)

Reference: Halvorsen et al. 2014[5]					
**Criteria**		**Yes (2)**	**Partial (1)**	**No (0)**	**N/A**
**1**	Question / objective sufficiently described?	2			
**2**	Study design evident and appropriate?	2			
**3**	Method of subject/comparison group selection or source of information/input variables described and appropriate?	2			
**4**	Subject and comparison group (if applicable) characteristics sufficiently described?		1 (randomized study with baseline characteristics reported for included anticoagulated AF cohort aged <65, 65-75 and ≥75 years, but not specifically compared between NOAC and VKA)		
**5**	If interventional and random allocation was possible, was it reported?	2			
**6**	If interventional and blinding of investigators was possible, was it reported?	2			
**7**	If interventional and blinding of subjects was possible, was it reported?	2			
**8**	Outcome and (if applicable) exposure measure(s) well defined and robust to measurement / misclassification bias? Means of assessment reported?	2			
**9**	Sample size appropriate?	2			
**10**	Analytic methods described/justified and appropriate?	2			
**11**	Some estimate of variance is reported for the main results?	2			
**12**	Controlling for confounding?	2			
**13**	Results reported in sufficient detail?	2			
**14**	Conclusion supported by the results?	2			
Total score: 27/28 (96.4%)					

F)

Reference: Kato et al. 2016[6]					
**Criteria**		**Yes (2)**	**Partial (1)**	**No (0)**	**N/A**
**1**	Question / objective sufficiently described?	2			
**2**	Study design evident and appropriate?	2			
**3**	Method of subject/comparison group selection or source of information/input variables described and appropriate?	2			
**4**	Subject and comparison group (if applicable) characteristics sufficiently described?		1 (randomized study with baseline characteristics reported for included anticoagulated AF cohort aged <65, 65-75 and ≥75 years, but not specifically compared between NOAC and VKA)		
**5**	If interventional and random allocation was possible, was it reported?	2			
**6**	If interventional and blinding of investigators was possible, was it reported?	2			
**7**	If interventional and blinding of subjects was possible, was it reported?	2			
**8**	Outcome and (if applicable) exposure measure(s) well defined and robust to measurement / misclassification bias? Means of assessment reported?	2			
**9**	Sample size appropriate?	2			
**10**	Analytic methods described/justified and appropriate?	2			
**11**	Some estimate of variance is reported for the main results?	2			
**12**	Controlling for confounding?	2			
**13**	Results reported in sufficient detail?	2			
**14**	Conclusion supported by the results?	2			
Total score: 27/28 (96.4%)					

G)

Reference: Chao et al. 2020[14]					
**Criteria**		**Yes (2)**	**Partial (1)**	**No (0)**	**N/A**
**1**	Question / objective sufficiently described?	2			
**2**	Study design evident and appropriate?	2			
**3**	Method of subject/comparison group selection or source of information/input variables described and appropriate?	2			
**4**	Subject and comparison group (if applicable) characteristics sufficiently described?	2			
**5**	If interventional and random allocation was possible, was it reported?				N/A
**6**	If interventional and blinding of investigators was possible, was it reported?				N/A
**7**	If interventional and blinding of subjects was possible, was it reported?				N/A
**8**	Outcome and (if applicable) exposure measure(s) well defined and robust to measurement / misclassification bias? Means of assessment reported?		1 (outcome measures retrospectively assessed in administrative healthcare claims database using ICD-codes, which are prone to misclassification bias)		
**9**	Sample size appropriate?	2			
**10**	Analytic methods described/justified and appropriate?	2			
**11**	Some estimate of variance is reported for the main results?	2			
**12**	Controlling for confounding?		1 (adequately adjusted for predefined set of covariates using propensity score matching, but due to retrospective use of administrative healthcare claims database, unmeasured confounders and biases (such as confounding by indication) may still be present)		
**13**	Results reported in sufficient detail?	2			
**14**	Conclusion supported by the results?	2			
Total score: 20/22 (90.9%)					

H)

Reference: Nishida et al. 2019[17]					
**Criteria**		**Yes (2)**	**Partial (1)**	**No (0)**	**N/A**
**1**	Question / objective sufficiently described?	2			
**2**	Study design evident and appropriate?	2			
**3**	Method of subject/comparison group selection or source of information/input variables described and appropriate?	2			
**4**	Subject and comparison group (if applicable) characteristics sufficiently described?		1 (prospective observational study with baseline characteristics reported for included anticoagulated AF cohort aged <75, 75-84 and ≥85 years, but not specifically compared between NOAC and VKA)		
**5**	If interventional and random allocation was possible, was it reported?				N/A
**6**	If interventional and blinding of investigators was possible, was it reported?				N/A
**7**	If interventional and blinding of subjects was possible, was it reported?				N/A
**8**	Outcome and (if applicable) exposure measure(s) well defined and robust to measurement / misclassification bias? Means of assessment reported?	2			
**9**	Sample size appropriate?		1 (limited sample sizes for subgroup of patients 75-84 years (569 NOAC, 509 warfarin) and ≥85 years old (121 NOAC, 143 warfarin)		
**10**	Analytic methods described/justified and appropriate?	2			
**11**	Some estimate of variance is reported for the main results?	2			
**12**	Controlling for confounding?	2			
**13**	Results reported in sufficient detail?	2			
**14**	Conclusion supported by the results?	2			
Total score: 20/22 (90.9%)					

I)

Reference: Hohmann et al. 2019[19]					
**Criteria**		**Yes (2)**	**Partial (1)**	**No (0)**	**N/A**
**1**	Question / objective sufficiently described?	2			
**2**	Study design evident and appropriate?	2			
**3**	Method of subject/comparison group selection or source of information/input variables described and appropriate?	2			
**4**	Subject and comparison group (if applicable) characteristics sufficiently described?		1 (retrospective observational study with baseline characteristics reported for included anticoagulated AF cohort aged <75 and ≥75 years, but not specifically compared between NOAC and VKA)		
**5**	If interventional and random allocation was possible, was it reported?				N/A
**6**	If interventional and blinding of investigators was possible, was it reported?				N/A
**7**	If interventional and blinding of subjects was possible, was it reported?				N/A
**8**	Outcome and (if applicable) exposure measure(s) well defined and robust to measurement / misclassification bias? Means of assessment reported?		1 (outcome measures retrospectively assessed in administrative healthcare claims database using ICD-codes, which are prone to misclassification bias)		
**9**	Sample size appropriate?	2			
**10**	Analytic methods described/justified and appropriate?	2			
**11**	Some estimate of variance is reported for the main results?	2			
**12**	Controlling for confounding?		1 (adequately adjusted for predefined set of covariates, but due to retrospective use of administrative healthcare claims database, unmeasured confounders and biases (such as confounding by indication) may still be present)		
**13**	Results reported in sufficient detail?	2			
**14**	Conclusion supported by the results?	2			
Total score: 19/22 (86.4%)					

J)

Reference: Avgil-Tsadok et al. 2016[24]					
**Criteria**		**Yes (2)**	**Partial (1)**	**No (0)**	**N/A**
**1**	Question / objective sufficiently described?	2			
**2**	Study design evident and appropriate?	2			
**3**	Method of subject/comparison group selection or source of information/input variables described and appropriate?	2			
**4**	Subject and comparison group (if applicable) characteristics sufficiently described?	2			
**5**	If interventional and random allocation was possible, was it reported?				N/A
**6**	If interventional and blinding of investigators was possible, was it reported?				N/A
**7**	If interventional and blinding of subjects was possible, was it reported?				N/A
**8**	Outcome and (if applicable) exposure measure(s) well defined and robust to measurement / misclassification bias? Means of assessment reported?		1 (outcome measures retrospectively assessed in administrative healthcare claims database using ICD-codes, which are prone to misclassification bias)		
**9**	Sample size appropriate?	2			
**10**	Analytic methods described/justified and appropriate?	2			
**11**	Some estimate of variance is reported for the main results?	2			
**12**	Controlling for confounding?		1 (adequately adjusted for predefined set of covariates, but due to retrospective use of administrative healthcare claims database, unmeasured confounders and biases (such as confounding by indication) may still be present)		
**13**	Results reported in sufficient detail?	2			
**14**	Conclusion supported by the results?	2			
Total score: 20/22 (90.9%)					

K)

Reference: Alcusky et al. 2020[25]					
**Criteria**		**Yes (2)**	**Partial (1)**	**No (0)**	**N/A**
**1**	Question / objective sufficiently described?	2			
**2**	Study design evident and appropriate?	2			
**3**	Method of subject/comparison group selection or source of information/input variables described and appropriate?	2			
**4**	Subject and comparison group (if applicable) characteristics sufficiently described?	2			
**5**	If interventional and random allocation was possible, was it reported?				N/A
**6**	If interventional and blinding of investigators was possible, was it reported?				N/A
**7**	If interventional and blinding of subjects was possible, was it reported?				N/A
**8**	Outcome and (if applicable) exposure measure(s) well defined and robust to measurement / misclassification bias? Means of assessment reported?		1 (outcome measures retrospectively assessed in administrative healthcare claims database using ICD-codes, which are prone to misclassification bias)		
**9**	Sample size appropriate?	2			
**10**	Analytic methods described/justified and appropriate?	2			
**11**	Some estimate of variance is reported for the main results?	2			
**12**	Controlling for confounding?		1 (adequately adjusted for predefined set of covariates using propensity score matching, but due to retrospective use of administrative healthcare claims database, unmeasured confounders and biases (such as confounding by indication) may still be present)		
**13**	Results reported in sufficient detail?	2			
**14**	Conclusion supported by the results?	2			
Total score: 20/22 (90.9%)					

L)

Reference: Wong et al. 2020[29]					
**Criteria**		**Yes (2)**	**Partial (1)**	**No (0)**	**N/A**
**1**	Question / objective sufficiently described?	2			
**2**	Study design evident and appropriate?	2			
**3**	Method of subject/comparison group selection or source of information/input variables described and appropriate?	2			
**4**	Subject and comparison group (if applicable) characteristics sufficiently described?		1 (retrospective observational study with baseline characteristics reported for included AF cohort using NOAC and VKA, but not specifically for subgroup aged ≥75 years)		
**5**	If interventional and random allocation was possible, was it reported?				N/A
**6**	If interventional and blinding of investigators was possible, was it reported?				N/A
**7**	If interventional and blinding of subjects was possible, was it reported?				N/A
**8**	Outcome and (if applicable) exposure measure(s) well defined and robust to measurement / misclassification bias? Means of assessment reported?		1 (outcome measures retrospectively assessed in administrative healthcare claims database using ICD-codes, which are prone to misclassification bias)		
**9**	Sample size appropriate?	2			
**10**	Analytic methods described/justified and appropriate?	2			
**11**	Some estimate of variance is reported for the main results?	2			
**12**	Controlling for confounding?		1 (adequately adjusted for predefined set of covariates, but due to retrospective use of administrative healthcare claims database, unmeasured confounders and biases (such as confounding by indication) may still be present)		
**13**	Results reported in sufficient detail?	2			
**14**	Conclusion supported by the results?	2			
Total score: 19/22 (86.4%)					

eTable 8: Assessment of bias within studies included in the meta-analysis (**A-F:** 6 post hoc analyses of randomized controlled trials; **G-L:** 6 longitudinal observational cohort studies) using the quality assessment tool 'QUALSYST' from the "Standard Quality Assessment Criteria for Evaluating Primary Research Papers

from a Variety of Fields" was used.[51] With this tool, 14 items of each quantitative study, were scored on the study and outcome levels depending on the degree to which the specific criteria were met or reported ("yes" = 2, "partial" = 1, "no" = 0). Items not applicable to a particular study design were marked "n/a" and were excluded from the calculation of the summary score. A percentage was calculated for each paper by dividing the total sum score obtained across rated items by the total possible score.

AF: atrial fibrillation; ICD: International Classification of Diseases.

1.9 eTable 9: PRISMA 2009 checklist

Section/topic	#	Checklist item	Reported on page #
**TITLE**			
Title	1	Identify the report as a systematic review, meta-analysis, or both.	1
**ABSTRACT**			
Structured summary	2	Provide a structured summary including, as applicable: background; objectives; data sources; study eligibility criteria, participants, and interventions; study appraisal and synthesis methods; results; limitations; conclusions and implications of key findings; systematic review registration number.	1-2
**INTRODUCTION**			
Rationale	3	Describe the rationale for the review in the context of what is already known.	2
Objectives	4	Provide an explicit statement of questions being addressed with reference to participants, interventions, comparisons, outcomes, and study design (PICOS).	2
**METHODS**			
Protocol and registration	5	Indicate if a review protocol exists, if and where it can be accessed (e.g., Web address), and, if available, provide registration information including registration number.	Not applicable
Eligibility criteria	6	Specify study characteristics (e.g., PICOS, length of follow-up) and report characteristics (e.g., years considered, language, publication status) used as criteria for eligibility, giving rationale.	2
Information sources	7	Describe all information sources (e.g., databases with dates of coverage, contact with study authors to identify additional studies) in the search and date last searched.	2
Search	8	Present full electronic search strategy for at least one database, including any limits used, such that it could be repeated.	eTable 1
Study selection	9	State the process for selecting studies (i.e., screening, eligibility, included in systematic review, and, if applicable, included in the meta-analysis).	2-4
Data collection process	10	Describe method of data extraction from reports (e.g., piloted forms, independently, in duplicate) and any processes for obtaining and confirming data from investigators.	3
Data items	11	List and define all variables for which data were sought (e.g., PICOS, funding sources) and any assumptions and simplifications made.	2-3
Risk of bias in individual studies	12	Describe methods used for assessing risk of bias of individual studies (including specification of whether this was done at the study or outcome level), and how this information is to be used in any data synthesis.	3-4
Summary measures	13	State the principal summary measures (e.g., risk ratio, difference in means).	2-3
Synthesis of results	14	Describe the methods of handling data and combining results of studies, if done, including measures of consistency (e.g., I^2) for each meta-analysis.	3-4

Section/topic	#	Checklist item	Reported on page #
Risk of bias across studies	15	Specify any assessment of risk of bias that may affect the cumulative evidence (e.g., publication bias, selective reporting within studies).	3-4
Additional analyses	16	Describe methods of additional analyses (e.g., sensitivity or subgroup analyses, meta-regression), if done, indicating which were pre-specified.	2 + 6-8 + eFigure 1-4
**RESULTS**			
Study selection	17	Give numbers of studies screened, assessed for eligibility, and included in the review, with reasons for exclusions at each stage, ideally with a flow diagram.	2-3 + Figure 1
Study characteristics	18	For each study, present characteristics for which data were extracted (e.g., study size, PICOS, follow-up period) and provide the citations.	eTable 2-7
Risk of bias within studies	19	Present data on risk of bias of each study and, if available, any outcome level assessment (see item 12).	eTable 8
Results of individual studies	20	For all outcomes considered (benefits or harms), present, for each study: (a) simple summary data for each intervention group (b) effect estimates and confidence intervals, ideally with a forest plot.	eTable 2-7
Synthesis of results	21	Present results of each meta-analysis done, including confidence intervals and measures of consistency.	6-8 + Figure 2-6
Risk of bias across studies	22	Present results of any assessment of risk of bias across studies (see Item 15).	8 + eFigure 5A-F
Additional analysis	23	Give results of additional analyses, if done (e.g., sensitivity or subgroup analyses, meta-regression [see Item 16]).	6-8 + eFigure 1-4
**DISCUSSION**			
Summary of evidence	24	Summarize the main findings including the strength of evidence for each main outcome; consider their relevance to key groups (e.g., healthcare providers, users, and policy makers).	10-11 + 14-15 + Table 1
Limitations	25	Discuss limitations at study and outcome level (e.g., risk of bias), and at review-level (e.g., incomplete retrieval of identified research, reporting bias).	14
Conclusions	26	Provide a general interpretation of the results in the context of other evidence, and implications for future research.	14-15
**FUNDING**			
Funding	27	Describe sources of funding for the systematic review and other support (e.g., supply of data); role of funders for the systematic review.	15

From: Moher D, Liberati A, Tetzlaff J, Altman DG, The PRISMA Group (2009). Preferred Reporting Items for Systematic Reviews and Meta-Analyses: The PRISMA Statement. PLoS Med 6(7): e1000097. doi:10.1371/journal.pmed1000097

For more information, visit: **www.prisma-statement.org**.

2 Supplemental figures

2.1 eFigure 1: Sensitivity analysis for all-cause mortality

Study	HR	95% CI	Weight
Lauw et al. 2017 (RCT: dabi 150)	0.82	[0.63-1.07]	7.4%
Lauw et al. 2017 (RCT: dabi 110)	0.86	[0.66-1.11]	7.9%
Alcusky et al. 2020 (Observational: dabi 150/110)	0.68	[0.59-0.79]	17.6%
Alcusky et al. 2020 (Observational: riva 20/15)	0.79	[0.72-0.87]	28.4%
Halvorsen et al. 2018 (RCT: api 5/2.5)	0.91	[0.77-1.07]	15.9%
Alcusky et al. 2020 (Observational: api 5/2.5)	0.78	[0.70-0.88]	22.8%
**Summary statistic (RE model)**	**0.79**	**[0.73-0.86]**	**100.0%**

Heterogeneity: I² = 34.7%, Q = 7.31, p(Q) = 0.20

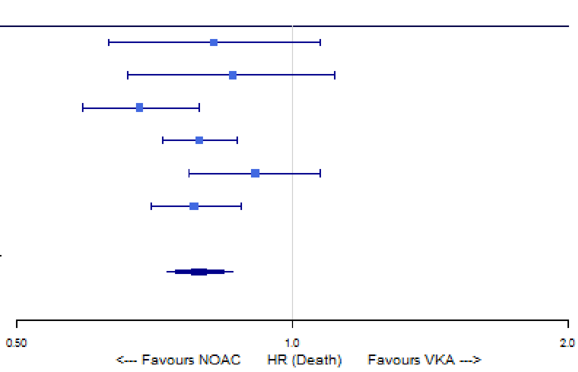

<u>eFigure 1:</u> Forest plot of the risk of all-cause mortality of NOACs versus VKAs in elderly atrial fibrillation patients ≥75 years old, after excluding the two observational studies with the most heterogeneous results.
Api 5/2.5: apixaban 5 mg (standard dose) and 2.5 mg (reduced dose); CI: confidence interval; Dabi 150: dabigatran 150 mg (standard dose); Dabi 110: dabigatran 110 mg (reduced dose); Death: all-cause mortality; HR: hazard ratio; NOAC: non-vitamin K antagonist oral anticoagulant; RCT: randomized controlled trial (post hoc analysis); RE model: random effects model Riva: rivaroxaban; Riva 20/15: rivaroxaban 20 mg (standard dose) and 15 mg (reduced dose); VKA: vitamin K antagonist

2.2 eFigure 2: Sensitivity analyses for major bleeding

A)

Study	HR	95% CI	Weight
Eikelboom et al. 2011 (RCT: dabi 150)	1.18	[0.98-1.42]	9.5%
Avgil-Tsadok et al. 2016 (Observational: dabi 150)	0.93	[0.79-1.10]	10.4%
Eikelboom et al. 2011 (RCT: dabi 110)	1.01	[0.83-1.23]	9.0%
Avgil-Tsadok et al. 2016 (Observational: dabi 110)	0.94	[0.86-1.03]	14.6%
Alcusky et al. 2020 (Observational: dabi 150/110)	1.10	[0.80-1.53]	4.8%
Wong et al. 2020 (Observational: dabi 150/110)	0.83	[0.78-0.87]	16.7%
Halperin et al. 2014 (RCT: riva 20/15)	1.11	[0.92-1.34]	9.4%
Hori et al. 2014 (RCT: riva 15/10)	1.51	[0.68-3.32]	1.1%
Alcusky et al. 2020 (Observational: riva 20/15)	1.07	[0.87-1.33]	8.1%
Wong et al. 2020 (Observational: riva 20/15)	1.06	[1.01-1.12]	16.4%
**Summary statistic (RE model)**	**1.00**	**[0.92-1.09]**	**100.0%**

Heterogeneity: I² = 76.8%, Q = 58.1, p(Q) <0.001

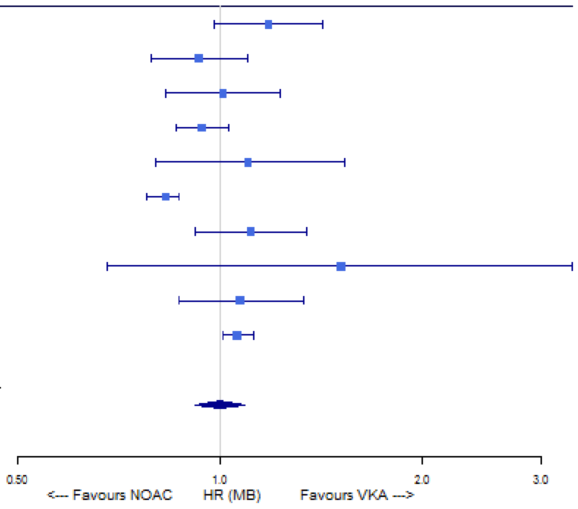

B)

Study	HR	95% CI	Weight
Eikelboom et al. 2011 (RCT: dabi 150)	1.18	[0.98-1.42]	34.4%
Eikelboom et al. 2011 (RCT: dabi 110)	1.01	[0.83-1.23]	30.4%
Halperin et al. 2014 (RCT: riva 20/15)	1.11	[0.92-1.34]	33.3%
Hori et al. 2014 (RCT: riva 15/10)	1.51	[0.68-3.32]	1.9%
**Summary statistic (RE model)**	**1.11**	**[0.99-1.24]**	**100.0%**

Heterogeneity: I² = 0.00%, Q = 1.89, p(Q) = 0.60

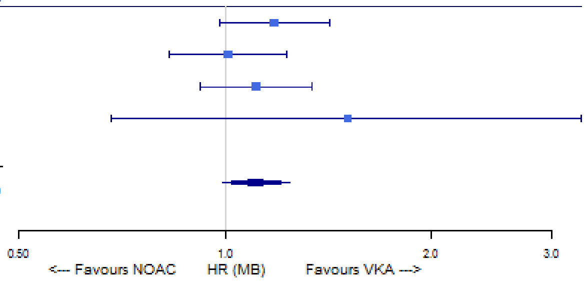

C)

Study	HR	95% CI	Weight
Avgil-Tsadok et al. 2016 (Observational: dabi 150)	0.93	[0.79-1.10]	14.5%
Avgil-Tsadok et al. 2016 (Observational: dabi 110)	0.94	[0.86-1.03]	20.7%
Alcusky et al. 2020 (Observational: dabi 150/110)	1.10	[0.80-1.53]	6.5%
Wong et al. 2020 (Observational: dabi 150/110)	0.83	[0.78-0.87]	23.8%
Alcusky et al. 2020 (Observational: riva 20/15)	1.07	[0.87-1.33]	11.2%
Wong et al. 2020 (Observational: riva 20/15)	1.06	[1.01-1.12]	23.3%
**Summary statistic (RE model)**	**0.96**	**[0.87-1.06]**	**100.0%**

Heterogeneity: I² = 82.6%, Q = 46.8, p(Q) <0.001

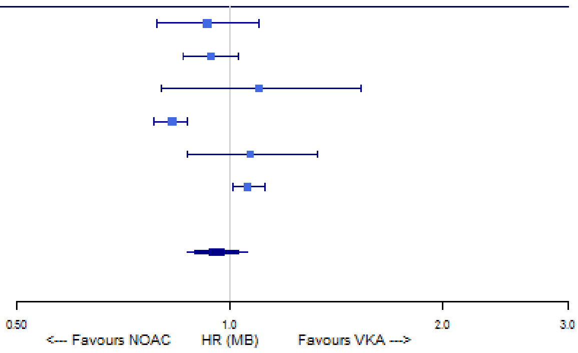

D)

Study	HR	95% CI	Weight
Halvorsen et al. 2018 (RCT: api 5/2.5)	0.64	[0.52-0.79]	23.3%
Alcusky et al. 2020 (Observational: api 5/2.5)	0.66	[0.49-0.88]	17.5%
Wong et al. 2020 (Observational: api 5/2.5)	0.89	[0.81-0.98]	33.0%
Kato et al. 2016 (RCT: edo 60/30)	0.83	[0.70-0.99]	26.2%
**Summary statistic (RE model)**	**0.77**	**[0.65-0.91]**	**100.0%**
Heterogeneity: I² = 70.9%, Q = 10.3, p(Q) = 0.016			

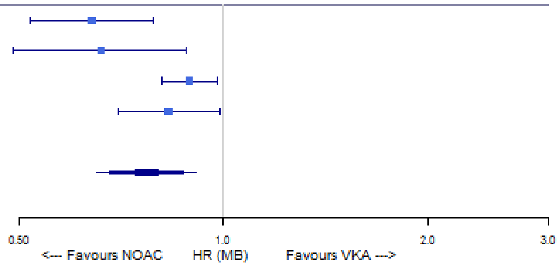

eFigure 2: Forest plot of the risk of major bleeding of NOACs versus VKAs in elderly atrial fibrillation patients ≥75 years old, stratified according to **A)** dabigatran and rivaroxaban versus VKAs, **B)** dabigatran and rivaroxaban versus VKAs (results from RCTs only), **C)** dabigatran and rivaroxaban versus VKAs (results from observational studies only), and **D)** apixaban and edoxaban versus VKAs.

Api 5/2.5: apixaban 5 mg (standard dose) and 2.5 mg (reduced dose); CI: confidence interval; Dabi 150: dabigatran 150 mg (standard dose); Dabi 110: dabigatran 110 mg (reduced dose); Edo 60/30: edoxaban 60 mg (standard dose) and 30 mg (reduced dose); HR: hazard ratio; MB: major bleeding; NOAC: non-vitamin K antagonist oral anticoagulant; RCT: randomized controlled trial (post hoc analysis); RE model: random effects model; Riva: rivaroxaban; Riva 20/15: rivaroxaban 20 mg (standard dose) and 15 mg (reduced dose); Riva 15/10: rivaroxaban 15 mg (standard dose) and 10 mg (reduced dose); VKA: vitamin K antagonist

2.3 eFigure 3: Sensitivity analyses for gastrointestinal bleeding

A)

Study	HR	95% CI	Weight
Eikelboom et al. 2011 (RCT: dabi 150)	1.79	[1.35-2.37]	9.2%
Avgil-Tsadok et al. 2016 (Observational: dabi 150)	1.35	[1.01-1.82]	8.7%
Eikelboom et al. 2011 (RCT: dabi 110)	1.39	[1.03-1.98]	7.3%
Avgil-Tsadok et al. 2016 (Observational: dabi 110)	1.31	[1.13-1.51]	13.6%
Hohmann et al. 2019 (Observational: dabi 150/110)	0.99	[0.71-1.38]	7.8%
Wong et al. 2020 (Observational: dabi 150/110)	0.95	[0.87-1.03]	15.4%
Hohmann et al. 2019 (Observational: riva 20/15)	1.44	[1.21-1.70]	12.8%
Wong et al. 2020 (Observational: riva 20/15)	1.32	[1.22-1.42]	15.5%
Kato et al. 2016 (RCT: edo 60/30)	1.32	[1.01-1.72]	9.7%
**Summary statistic (RE model)**	**1.28**	**[1.13-1.46]**	**100.0%**

Heterogeneity: I² = 82.6%, Q = 55.6, p(Q) < 0.001

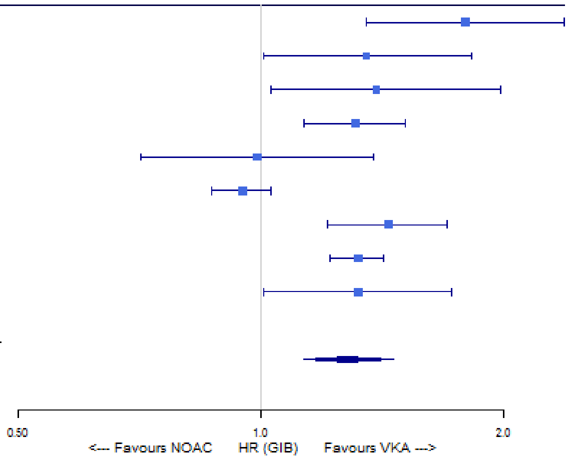

B)

Study	HR	95% CI	Weight
Eikelboom et al. 2011 (RCT: dabi 150)	1.79	[1.35-2.37]	15.6%
Avgil-Tsadok et al. 2016 (Observational: dabi 150)	1.35	[1.01-1.82]	14.9%
Eikelboom et al. 2011 (RCT: dabi 110)	1.39	[1.03-1.98]	13.1%
Avgil-Tsadok et al. 2016 (Observational: dabi 110)	1.31	[1.13-1.51]	20.5%
Hohmann et al. 2019 (Observational: dabi 150/110)	0.99	[0.71-1.38]	13.8%
Wong et al. 2020 (Observational: dabi 150/110)	0.95	[0.87-1.03]	22.1%
**Summary statistic (RE model)**	**1.25**	**[1.03-1.52]**	**100.0%**

Heterogeneity: I² = 81.9%, Q = 33.6, p(Q) < 0.001

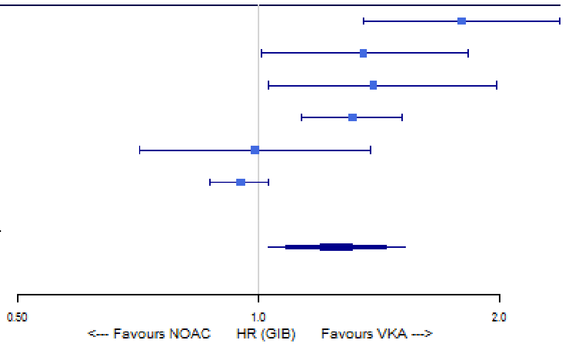

C)

Study	HR	95% CI	Weight
Hohmann et al. 2019 (Observational: riva 20/15)	1.44	[1.21-1.70]	16.2%
Wong et al. 2020 (Observational: riva 20/15)	1.32	[1.22-1.42]	83.8%
**Summary statistic (RE model)**	**1.34**	**[1.25-1.43]**	**100.0%**

Heterogeneity: I² = 0.00%, Q = 0.88, p(Q) = 0.35

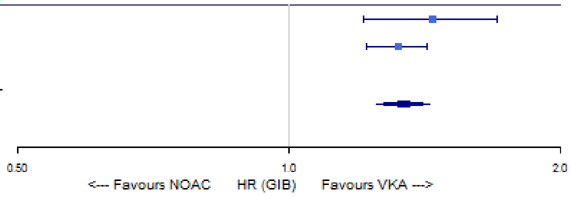

D)

Study	HR	95% CI	Weight
Hohmann et al. 2019 (Observational: api 5/2.5)	0.64	[0.50-0.81]	46.7%
Wong et al. 2020 (Observational: api 5/2.5)	0.93	[0.80-1.07]	53.3%
**Summary statistic (RE model)**	**0.78**	**[0.54-1.13]**	**100.0%**
Heterogeneity: I² = 86.0%, Q = 7.14, p(Q) = 0.008			

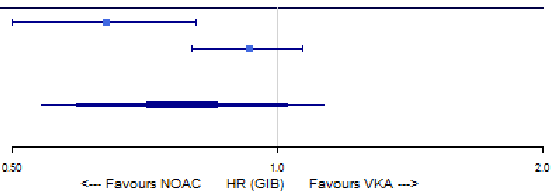

<u>eFigure 3:</u> Forest plot of the risk of gastrointestinal bleeding of NOACs versus VKAs in elderly atrial fibrillation patients ≥75 years old, stratified according to **A)** dabigatran, rivaroxaban and edoxaban versus VKAs, **B)** dabigatran versus VKAs, **C)** rivaroxaban versus VKAs, and **D)** apixaban versus VKAs.

Api 5/2.5: apixaban 5 mg (standard dose) and 2.5 mg (reduced dose); CI: confidence interval; Dabi 150: dabigatran 150 mg (standard dose); Dabi 110: dabigatran 110 mg (reduced dose); Edo 60/30: edoxaban 60 mg (standard dose) and 30 mg (reduced dose); GIB: gastrointestinal bleeding; HR: hazard ratio; NOAC: non-vitamin K antagonist oral anticoagulant; RCT: randomized controlled trial (post hoc analysis); RE model: random effects model; Riva: rivaroxaban; Riva 20/15: rivaroxaban 20 mg (standard dose) and 15 mg (reduced dose); VKA: vitamin K antagonist

2.4 eFigure 4: Subgroup analyses including studies with patients ≥75, ≥80, ≥85 or ≥90 years old.

A)

Study	HR	95% CI	Weight
Eikelboom et al. 2011 (RCT: dabi 150, 75+)	0.67	[0.49-0.90]	7.8%
Eikelboom et al. 2011 (RCT: dabi 110, 75+)	0.88	[0.66-1.17]	8.1%
Halperin et al. 2014 (RCT: riva 20/15, 75+)	0.80	[0.63-1.02]	9.4%
Hori et al. 2014 (RCT: riva 15/10, 75+)	0.51	[0.20-1.27]	1.3%
Halvorsen et al. 2018 (RCT: api 5/2.5, 75+)	0.71	[0.53-0.95]	7.9%
Kato et al. 2016 (RCT: edo 60/30, 75+)	0.83	[0.66-1.04]	10.0%
Nishida et al. 2019 (Obs: pooled NOAC, 75+)	1.30	[0.73-2.33]	3.0%
Hohmann et al. 2019 (Obs: dabi/riva/api, 75+)	0.97	[0.83-1.14]	12.6%
Kim et al. 2019 (obs: pooled NOAC, 80+)	0.13	[0.04-0.48]	0.7%
Russo et al. 2019 (obs: pooled NOAC, 80+)	1.10	[0.49-2.45]	1.7%
Shinohara et al. 2019 (obs: pooled NOAC, 80+)	0.63	[0.16-2.57]	0.6%
Deitelzweig et al. 2019 (obs: dabi 150/110, 80+)	0.77	[0.60-0.99]	9.1%
Deitelzweig et al. 2019 (obs: riva 20/15, 80+)	0.74	[0.65-0.85]	13.6%
Deitelzweig et al. 2019 (obs: api 5/2.5, 80+)	0.58	[0.49-0.69]	12.1%
Nishida et al. 2019 (obs: pooled NOAC, 85+)	0.49	[0.15-1.56]	0.9%
Giustozzi et al. 2019 (obs: pooled NOAC, 90+)	0.78	[0.30-2.04]	1.2%
**Summary statistic (RE model)**	**0.76**	**[0.68-0.85]**	**100.0%**

Heterogeneity: I^2 = 47.3%, Q = 33.3, p(Q) = 0.004

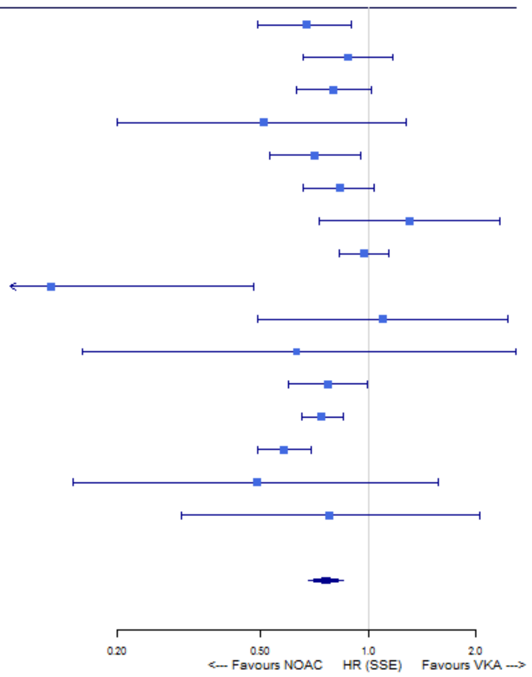

B)

Study	HR	95% CI	Weight
Eikelboom et al. 2011 (RCT: dabi 150, 75+)	1.18	[0.98-1.42]	4.9%
Avgil-Tsadok et al. 2016 (Obs: dabi 150, 75+)	0.93	[0.79-1.10]	5.1%
Eikelboom et al. 2011 (RCT: dabi 110, 75+)	1.01	[0.83-1.23]	4.7%
Avgil-Tsadok et al. 2016 (Obs: dabi 110, 75+)	0.94	[0.86-1.03]	5.9%
Alcusky et al. 2020 (Obs: dabi 150/110, 75+)	1.10	[0.80-1.53]	3.2%
Wong et al. 2020 (Obs: dabi 150/110, 75+)	0.83	[0.78-0.87]	6.2%
Halperin et al. 2014 (RCT: riva 20/15, 75+)	1.11	[0.92-1.34]	4.8%
Hori et al. 2014 (RCT: riva 15/10, 75+)	1.51	[0.68-3.32]	1.0%
Alcusky et al. 2020 (Obs: riva 20/15, 75+)	1.07	[0.87-1.33]	4.5%
Wong et al. 2020 (Obs: riva 20/15, 75+)	1.06	[1.01-1.12]	6.0%
Halvorsen et al. 2018 (RCT: api 5/2.5, 75+)	0.64	[0.52-0.79]	4.6%
Alcusky et al. 2020 (Obs: api 5/2.5, 75+)	0.66	[0.49-0.88]	3.7%
Wong et al. 2020 (Obs: api 5/2.5, 75+)	0.89	[0.81-0.98]	5.8%
Kato et al. 2016 (RCT: edo 60/30, 75+)	0.83	[0.70-0.99]	5.0%
Chao et al. 2020 (Ob: dabi/riva/api, 75+)	0.86	[0.80-0.92]	6.1%
Nishida et al. 2019 (Obs: pooled NOAC, 75+)	1.11	[0.61-2.01]	1.6%
Kim et al. 2019 (obs: pooled NOAC, 80+)	0.11	[0.02-0.49]	0.3%
Russo et al. 2019 (obs: pooled NOAC, 80+)	0.89	[0.53-1.50]	1.9%
Deitelzweig et al. 2019 (obs: dabi 150/110, 80+)	0.92	[0.78-1.07]	5.3%
Deitelzweig et al. 2019 (obs: riva 20/15, 80+)	1.16	[1.07-1.24]	6.1%
Deitelzweig et al. 2019 (obs: api 5/2.5, 80+)	0.60	[0.54-0.67]	5.7%
Poli et al. 2019 (obs: pooled NOAC, 85+)	0.88	[0.42-1.80]	1.2%
Giustozzi et al. 2019 (obs: pooled NOAC, 90+)	1.43	[0.77-2.65]	1.5%
Chao et al. 2020 (Obs: dabi/riva/api, 90+)	0.86	[0.72-1.03]	4.9%
**Summary statistic (RE model)**	**0.92**	**[0.84-0.998]**	**100.0%**

Heterogeneity: I^2 = 89.1%, Q = 197.0, p(Q) <0.001

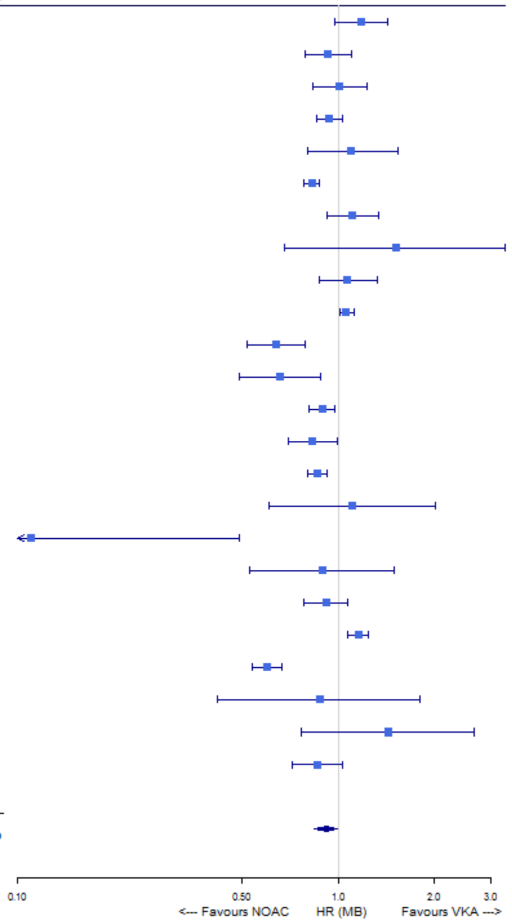

C)

Study	HR	95% CI	Weight
Eikelboom et al. 2011 (RCT: dabi 150, 75+)	0.42	[0.25-0.70]	3.9%
Avgil-Tsadok et al. 2016 (Obs: dabi 150, 75+)	0.79	[0.50-1.25]	4.4%
Eikelboom et al. 2011 (RCT: dabi 110, 75+)	0.37	[0.21-0.64]	3.5%
Avgil-Tsadok et al. 2016 (Obs: dabi 110, 75+)	0.55	[0.42-0.73]	6.9%
Wong et al. 2020 (Obs: dabi 150/110, 75+)	0.59	[0.49-0.71]	8.5%
Halperin et al. 2014 (RCT: riva 20/15, 75+)	0.80	[0.50-1.28]	4.3%
Wong et al. 2020 (Obs: riva 20/15, 75+)	0.81	[0.69-0.96]	8.8%
Halvorsen et al. 2018 (RCT: api 5/2.5, 75+)	0.34	[0.20-0.57]	3.8%
Wong et al. 2020 (Obs: api 5/2.5, 75+)	0.70	[0.53-0.94]	6.7%
Kato et al. 2016 (RCT: edo 60/30, 75+)	0.40	[0.26-0.62]	4.6%
Chao et al. 2020 (Obs: dabi/riva/api, 75+)	0.56	[0.47-0.67]	8.6%
Hohmann et al. 2019 (Obs: dabi/riva/api, 75+)	0.59	[0.47-0.73]	8.0%
Kim et al. 2019 (obs: pooled NOAC, 80+)	0.024	[0.002-0.35]	0.2%
Russo et al. 2019 (obs: pooled NOAC, 80+)	0.33	[0.07-1.45]	0.7%
Deitelzweig et al. 2019 (obs: dabi 150/110, 80+)	0.51	[0.33-0.79]	4.7%
Deitelzweig et al. 2019 (obs: riva 20/15, 80+)	0.78	[0.64-0.95]	8.3%
Deitelzweig et al. 2019 (obs: api 5/2.5, 80+)	0.53	[0.41-0.68]	7.4%
Lai et al. 2018 (obs: dabi 110, 85+)	0.31	[0.10-0.97]	1.1%
Lai et al. 2018 (obs: riva 20/15, 85+)	0.47	[0.17-1.26]	1.4%
Chao et al. 2020 (Obs: dabi/riva/api, 90+)	0.36	[0.23-0.58]	4.2%
**Summary statistic (RE model)**	**0.56**	**[0.50-0.64]**	**100.0%**

Heterogeneity: I^2 = 61.7%, Q = 49.8, p(Q) <0.001

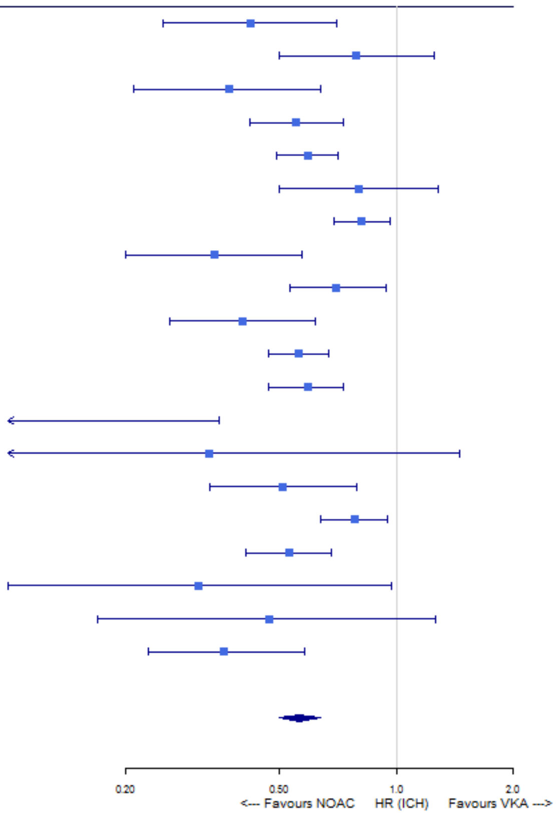

D)

Study	HR	95% CI	Weight
Eikelboom et al. 2011 (RCT: dabi 150, 75+)	1.79	[1.35-2.37]	5.9%
Avgil-Tsadok et al. 2016 (Obs: dabi 150, 75+)	1.35	[1.01-1.82]	5.8%
Eikelboom et al. 2011 (RCT: dabi 110, 75+)	1.39	[1.03-1.98]	5.3%
Avgil-Tsadok et al. 2016 (Obs: dabi 110, 75+)	1.31	[1.13-1.51]	7.0%
Hohmann et al. 2019 (Obs dabi 150/110, 75+)	0.99	[0.71-1.38]	5.5%
Wong et al. 2020 (Obs: dabi 150/110, 75+)	0.95	[0.87-1.03]	7.4%
Hohmann et al. 2019 (Obs: riva 20/15, 75+)	1.44	[1.21-1.70]	6.9%
Wong et al. 2020 (Obs: riva 20/15, 75+)	1.32	[1.22-1.42]	7.4%
Hohmann et al. 2019 (Obs: api 5/2.5, 75+)	0.64	[0.50-0.81]	6.3%
Wong et al. 2020 (Obs: api 5/2.5, 75+)	0.93	[0.80-1.07]	7.0%
Kato et al. 2016 (RCT: edo 60/30, 75+)	1.32	[1.01-1.72]	6.1%
Kim et al. 2019 (obs: pooled NOAC, 80+)	0.37	[0.047-2.95]	0.5%
Deitelzweig et al. 2019 (obs: dabi 150/110, 80+)	1.17	[0.94-1.46]	6.4%
Deitelzweig et al. 2019 (obs: riva 20/15, 80+)	1.33	[1.20-1.47]	7.3%
Deitelzweig et al. 2019 (obs: api 5/2.5, 80+)	0.62	[0.53-0.72]	7.0%
Lai et al. 2018 (obs: dabi 110, 85+)	1.21	[0.76-1.91]	4.4%
Lai et al. 2018 (obs: riva 20/15, 85+)	0.81	[0.47-1.38]	3.8%
**Summary statistic (RE model)**	**1.11**	**[0.96-1.29]**	**100.0%**

Heterogeneity: I^2 = 91.9%, Q = 167.0, p(Q) < 0.001

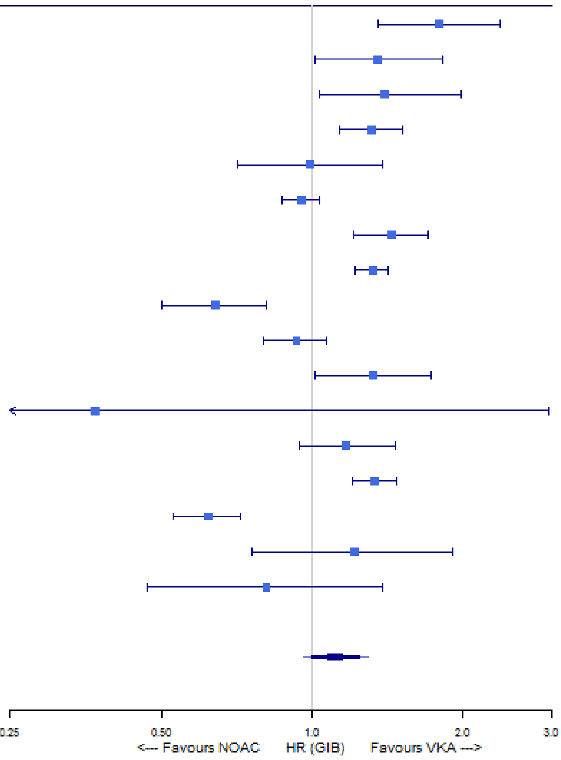

E)

Study	HR	95% CI	Weight
Lauw et al. 2017 (RCT: dabi 150, 75+)	0.82	[0.63-1.07]	4.6%
Lauw et al. 2017 (RCT: dabi 110, 75+)	0.86	[0.66-1.11]	4.7%
Alcusky et al. 2020 (Obs: dabi 150/110, 75+)	0.68	[0.59-0.79]	5.5%
Alcusky et al. 2020 (Obs: riva 20/15, 75+)	0.79	[0.72-0.87]	5.9%
Halvorsen et al. 2018 (RCT: api 5/2.5, 75+)	0.91	[0.77-1.07]	5.4%
Alcusky et al. 2020 (Obs: api 5/2.5, 75+)	0.78	[0.70-0.88]	5.7%
Chao et al. 2020 (Obs: dabi/riva/api, 75+)	0.50	[0.48-0.53]	6.0%
Nishida et al. 2019 (Obs: pooled NOAC, 75+)	1.27	[0.92-1.97]	3.2%
Kim et al. 2019 (obs: pooled NOAC, 80+)	0.30	[0.11-0.82]	1.1%
Russo et al. 2019 (obs: pooled NOAC, 80+)	0.65	[0.47-0.90]	4.1%
Deitelzweig et al. 2019 (obs: dabi 150/110, 80+)	0.87	[0.75-0.99]	5.7%
Deitelzweig et al. 2019 (obs: riva 20/15, 80+)	0.87	[0.81-0.93]	6.0%
Deitelzweig et al. 2019 (obs: api 5/2.5, 80+)	0.61	[0.56-0.67]	5.9%
Lauw et al. 2017 (RCT: dabi 150, 80+)	1.16	[0.87-1.55]	4.4%
Lauw et al. 2017 (RCT: dabi 110, 80+)	1.09	[0.80-1.47]	4.3%
Lai et al. 2018 (obs: dabi 110, 85+)	0.59	[0.45-0.77]	4.6%
Lai et al. 2018 (obs: riva 20/15, 85+)	0.61	[0.47-0.79]	4.7%
Poli et al. 2019 (obs: pooled NOAC, 85+)	0.64	[0.46-0.91]	3.9%
Nishida et al. 2019 (Obs: pooled NOAC, 85+)	0.67	[0.33-1.33]	1.9%
Lauw et al. 2017 (RCT: dabi 150, 85+)	1.15	[0.74-1.79]	3.2%
Lauw et al. 2017 (RCT: dabi 110, 85+)	1.37	[0.89-2.11]	3.3%
Chao et al. 2020 (Obs: dabi/riva/api, 90+)	0.58	[0.52-0.64]	5.9%
**Summary statistic (RE model)**	**0.77**	**[0.69-0.86]**	**100.0%**

Heterogeneity: $I^2 = 91.1\%$, Q = 270.5, p(Q) <0.001

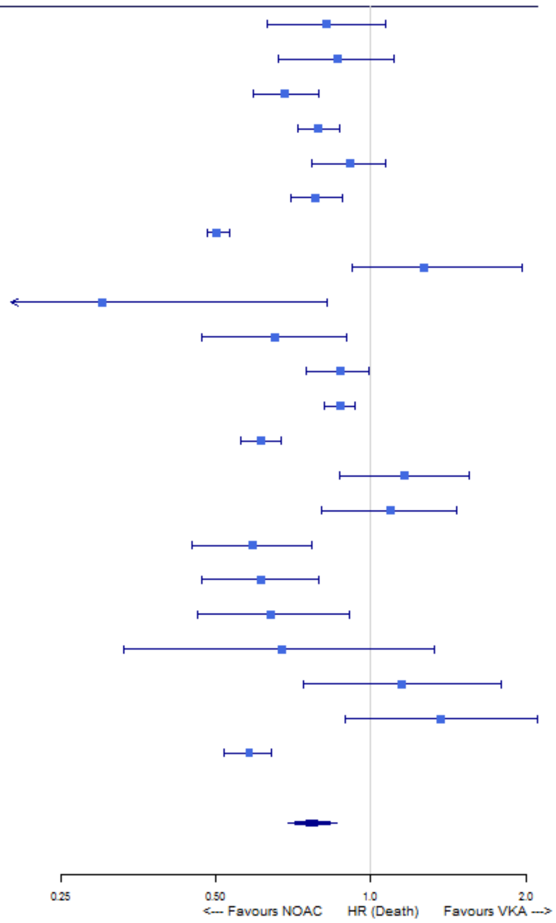

<u>eFigure 4:</u> Forest plot of the risk of **A)** stroke or systemic embolism, **B)** major bleeding, **C)** intracranial bleeding, **D)** gastrointestinal bleeding and **E)** all-cause mortality in elderly atrial fibrillation patients ≥75, ≥80, ≥85 or ≥90 years old.

Api 5/2.5: apixaban 5 mg (standard dose) and 2.5 mg (reduced dose); CI: confidence interval; Dabi 150: dabigatran 150 mg (standard dose); Dabi 110: dabigatran 110 mg (reduced dose); Death: all-cause mortality; Edo 60/30: edoxaban 60 mg (standard dose) and 30 mg (reduced dose); GIB: gastrointestinal bleeding; HR: hazard ratio; ICH: intracranial bleeding; MB: major bleeding; NOAC: non-vitamin K antagonist oral anticoagulant; Obs: longitudinal observational cohort study; RCT: randomized controlled trial (post hoc analysis); RE model: random effects model; Riva: rivaroxaban; Riva 20/15: rivaroxaban 20 mg (standard dose) and 15 mg (reduced dose); Riva 15/10: rivaroxaban 15 mg (standard dose) and 10 mg (reduced dose); Stroke/SE: stroke/systemic embolism; VKA: vitamin K antagonist.

2.5 eFigure 5: Assessment of publication bias at outcome level

A)

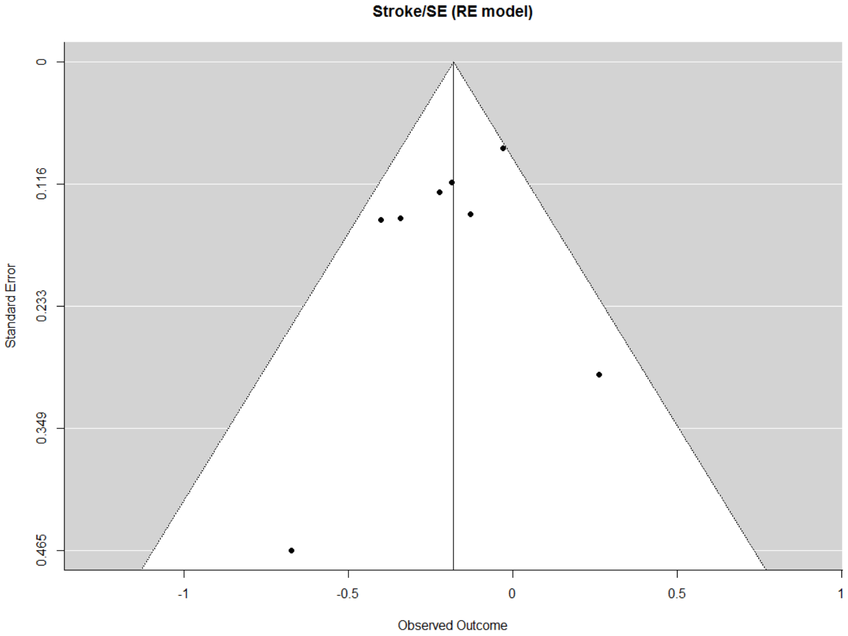

Egger's test: test for funnel plot asymmetry: z = -0.8177, p = 0.4135

B)

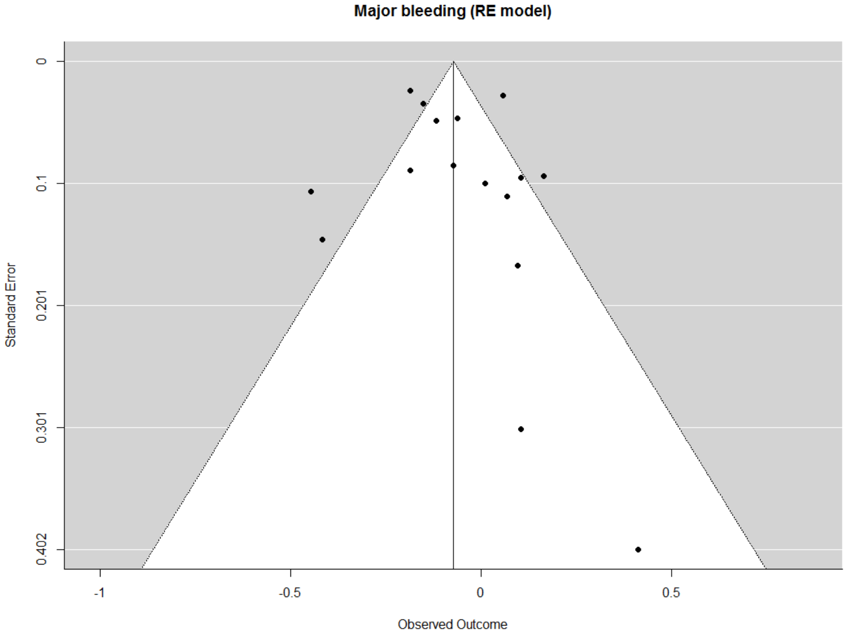

Egger's test: test for funnel plot asymmetry: z = 0.8869, p = 0.3751

C)

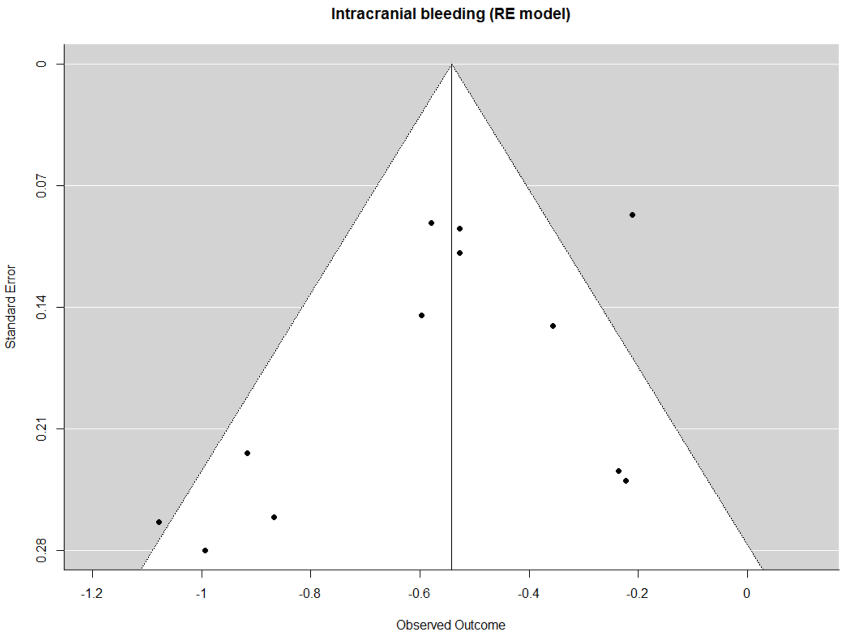

Egger's test: test for funnel plot asymmetry: z = -1.8763, p = 0.0606

D)

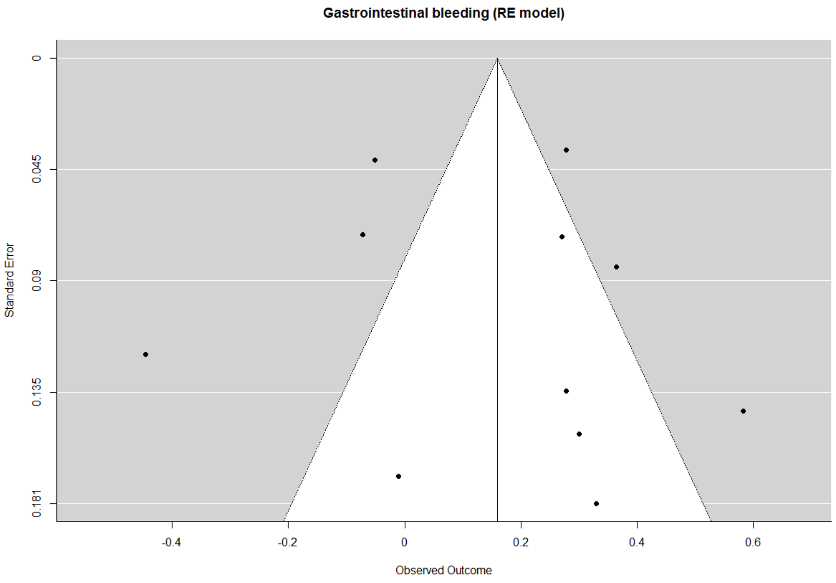

Egger's test: test for funnel plot asymmetry: z = 0.4875, p = 0.6259

E)

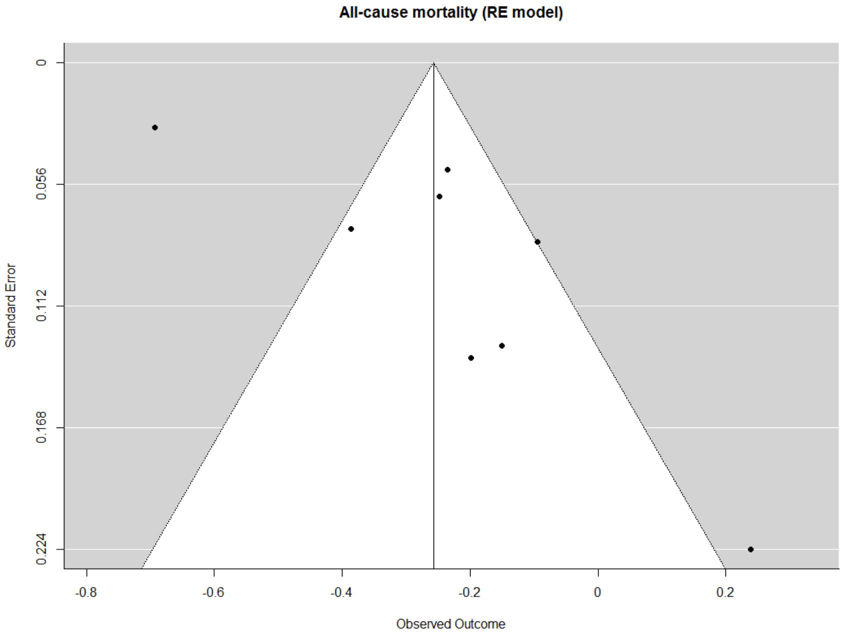

Egger's test: test for funnel plot asymmetry: z = 2.7487, p = 0.0060

F)

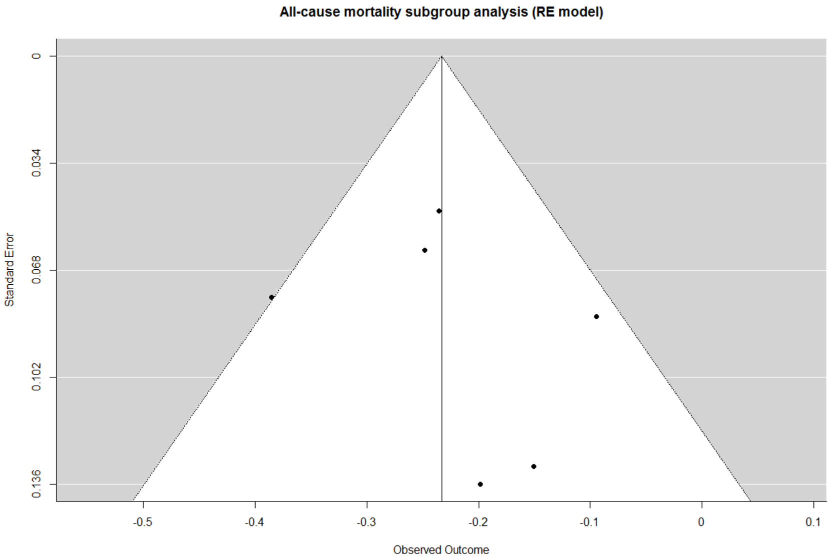

Egger's test: test for funnel plot asymmetry: z = 0.6227, p = 0.5335

<u>eFigure 5:</u> Funnel plot and Egger's test for assessment of potential publication bias for studies on **A)** stroke/SE, **B)** major bleeding, **C)** intracranial bleeding, **D)** gastrointestinal bleeding, **E)** all-cause mortality and **F)** subgroup analysis of all-cause mortality (after exclusion of two observational studies with the most heterogeneous results).
RE model: random effects model

3 References

1. Eikelboom JW, Wallentin L, Connolly SJ, Ezekowitz M, Healey JS, Oldgren J, et al. Risk of bleeding with 2 doses of dabigatran compared with warfarin in older and younger patients with atrial fibrillation: an analysis of the randomized evaluation of long-term anticoagulant therapy (RE-LY) trial. Circulation. 2011;123(21):2363-72.
2. Lauw MN, Eikelboom JW, Coppens M, Wallentin L, Yusuf S, Ezekowitz M, et al. Effects of dabigatran according to age in atrial fibrillation. Heart. 2017;103(13):1015-23.
3. Halperin JL, Hankey GJ, Wojdyla DM, Piccini JP, Lokhnygina Y, Patel MR, et al. Efficacy and safety of rivaroxaban compared with warfarin among elderly patients with nonvalvular atrial fibrillation in the Rivaroxaban Once Daily, Oral, Direct Factor Xa Inhibition Compared With Vitamin K Antagonism for Prevention of Stroke and Embolism Trial in Atrial Fibrillation (ROCKET AF). Circulation. 2014;130(2):138-46.
4. Hori M, Matsumoto M, Tanahashi N, Momomura S, Uchiyama S, Goto S, et al. Rivaroxaban vs. warfarin in Japanese patients with non-valvular atrial fibrillation in relation to age. Circulation journal : official journal of the Japanese Circulation Society. 2014;78(6):1349-56.
5. Halvorsen S, Atar D, Yang H, De Caterina R, Erol C, Garcia D, et al. Efficacy and safety of apixaban compared with warfarin according to age for stroke prevention in atrial fibrillation: observations from the ARISTOTLE trial. Eur Heart J. 2014;35(28):1864-72.
6. Kato ET, Giugliano RP, Ruff CT, Koretsune Y, Yamashita T, Kiss RG, et al. Efficacy and Safety of Edoxaban in Elderly Patients With Atrial Fibrillation in the ENGAGE AF-TIMI 48 Trial. J Am Heart Assoc. 2016;5(5).
7. Ruff CT, Giugliano RP, Braunwald E, Hoffman EB, Deenadayalu N, Ezekowitz MD, et al. Comparison of the efficacy and safety of new oral anticoagulants with warfarin in patients with atrial fibrillation: a meta-analysis of randomised trials. Lancet (London, England). 2014;383(9921):955-62.
8. Sadlon AH, Tsakiris DA. Direct oral anticoagulants in the elderly: systematic review and meta-analysis of evidence, current and future directions. Swiss medical weekly. 2016;146:w14356.
9. Caldeira D, Nunes-Ferreira A, Rodrigues R, Vicente E, Pinto FJ, Ferreira JJ. Non-vitamin K antagonist oral anticoagulants in elderly patients with atrial fibrillation: A systematic review with meta-analysis and trial sequential analysis. Archives of gerontology and geriatrics. 2019;81:209-14.
10. Kim IS, Kim HJ, Kim TH, Uhm JS, Joung B, Lee MH, et al. Non-vitamin K antagonist oral anticoagulants have better efficacy and equivalent safety compared to warfarin in elderly patients with atrial fibrillation: A systematic review and meta-analysis. Journal of cardiology. 2018;72(2):105-12.
11. Malik AH, Yandrapalli S, Aronow WS, Panza JA, Cooper HA. Meta-Analysis of Direct-Acting Oral Anticoagulants Compared With Warfarin in Patients >75 Years of Age. The American journal of cardiology. 2019;123(12):2051-7.
12. Lin L, Lim WS, Zhou HJ, Khoo AL, Tan KT, Chew AP, et al. Clinical and Safety Outcomes of Oral Antithrombotics for Stroke Prevention in Atrial Fibrillation: A Systematic Review and Network Meta-analysis. Journal of the American Medical Directors Association. 2015;16(12):1103.e1-19.
13. Deng K, Cheng J, Rao S, Xu H, Li L, Gao Y. Efficacy and Safety of Direct Oral Anticoagulants in Elderly Patients With Atrial Fibrillation: A Network Meta-Analysis. Frontiers in medicine. 2020;7:107.
14. Chao TF, Chiang CE, Liao JN, Chen TJ, Lip GYH, Chen SA. Comparing the Effectiveness and Safety of Nonvitamin K Antagonist Oral Anticoagulants and Warfarin in Elderly Asian Patients With Atrial Fibrillation: A Nationwide Cohort Study. Chest. 2020.
15. Deitelzweig S, Keshishian A, Li X, Kang A, Dhamane AD, Luo X, et al. Comparisons between Oral Anticoagulants among Older Nonvalvular Atrial Fibrillation Patients. Journal of the American Geriatrics Society. 2019;67(8):1662-71.
16. Raposeiras-Roubín S, Alonso Rodríguez D, Camacho Freire SJ, Abu-Assi E, Cobas-Paz R, Rodríguez Pascual C, et al. Vitamin K Antagonists and Direct Oral Anticoagulants in Nonagenarian Patients With Atrial Fibrillation. Journal of the American Medical Directors Association. 2020;21(3):367-73.e1.
17. Nishida T, Okumura Y, Yokoyama K, Matsumoto N, Tachibana E, Kuronuma K, et al. Oral anticoagulant use and clinical outcomes in elderly Japanese patients: findings from the SAKURA AF Registry. Heart and vessels. 2019.

18. Kim HM, Choi EK, Park CS, Cha MJ, Lee SY, Kwon JM, et al. Effectiveness and safety of non-vitamin K antagonist oral anticoagulants in octogenarian patients with non-valvular atrial fibrillation. PloS one. 2019;14(3):e0211766.
19. Hohmann C, Hohnloser SH, Jacob J, Walker J, Baldus S, Pfister R. Non-Vitamin K Oral Anticoagulants in Comparison to Phenprocoumon in Geriatric and Non-Geriatric Patients with Non-Valvular Atrial Fibrillation. Thromb Haemost. 2019;119(6):971-80.
20. Mitchell A, Watson MC, Welsh T, McGrogan A. Effectiveness and Safety of Direct Oral Anticoagulants versus Vitamin K Antagonists for People Aged 75 Years and over with Atrial Fibrillation: A Systematic Review and Meta-Analyses of Observational Studies. Journal of clinical medicine. 2019;8(4).
21. Russo V, Attena E, Di Maio M, Mazzone C, Carbone A, Parisi V, et al. Clinical profile of direct oral anticoagulants versus vitamin K anticoagulants in octogenarians with atrial fibrillation: a multicentre propensity score matched real-world cohort study. J Thromb Thrombolysis. 2019.
22. Shinohara M, Wada R, Yao S, Yano K, Akitsu K, Koike H, et al. Evaluation of oral anticoagulants in atrial fibrillation patients over 80 years of age with nonsevere frailty. Journal of arrhythmia. 2019;35(6):795-803.
23. Giustozzi M, Vedovati MC, Verso M, Scrucca L, Conti S, Verdecchia P, et al. Patients aged 90years or older with atrial fibrillation treated with oral anticoagulants: A multicentre observational study. Int J Cardiol. 2019;281:56-61.
24. Avgil-Tsadok M, Jackevicius CA, Essebag V, Eisenberg MJ, Rahme E, Behlouli H, et al. Dabigatran use in elderly patients with atrial fibrillation. Thromb Haemost. 2016;115(1):152-60.
25. Alcusky M, Tjia J, McManus DD, Hume AL, Fisher M, Lapane KL. Comparative Safety and Effectiveness of Direct-Acting Oral Anticoagulants Versus Warfarin: a National Cohort Study of Nursing Home Residents. Journal of general internal medicine. 2020.
26. Lai CL, Chen HM, Liao MT, Lin TT. Dabigatran, Rivaroxaban, and Warfarin in the Oldest Adults with Atrial Fibrillation in Taiwan. Journal of the American Geriatrics Society. 2018;66(8):1567-74.
27. Poli D, Antonucci E, Ageno W, Bertu L, Migliaccio L, Martinese L, et al. Oral anticoagulation in very elderly patients with atrial fibrillation: Results from the prospective multicenter START2-REGISTER study. PloS one. 2019;14(5):e0216831.
28. Shah SJ, Singer DE, Fang MC, Reynolds K, Go AS, Eckman MH. Net Clinical Benefit of Oral Anticoagulation Among Older Adults With Atrial Fibrillation. Circulation Cardiovascular quality and outcomes. 2019;12(11):e006212.
29. Wong JM, Maddox TM, Kennedy K, Shaw RE. Comparing Major Bleeding Risk in Outpatients With Atrial Fibrillation or Flutter by Oral Anticoagulant Type (from the National Cardiovascular Disease Registry's Practice Innovation and Clinical Excellence Registry). The American journal of cardiology. 2020.
30. Alexander KP, Brouwer MA, Mulder H, Vinereanu D, Lopes RD, Proietti M, et al. Outcomes of apixaban versus warfarin in patients with atrial fibrillation and multi-morbidity: Insights from the ARISTOTLE trial. Am Heart J. 2019;208:123-31.
31. Connolly SJ, Ezekowitz MD, Yusuf S, Eikelboom J, Oldgren J, Parekh A, et al. Dabigatran versus warfarin in patients with atrial fibrillation. N Engl J Med. 2009;361(12):1139-51.
32. Oldgren J, Alings M, Darius H, Diener HC, Eikelboom J, Ezekowitz MD, et al. Risks for stroke, bleeding, and death in patients with atrial fibrillation receiving dabigatran or warfarin in relation to the CHADS2 score: a subgroup analysis of the RE-LY trial. Annals of internal medicine. 2011;155(10):660-7, w204.
33. Patel MR, Mahaffey KW, Garg J, Pan G, Singer DE, Hacke W, et al. Rivaroxaban versus warfarin in nonvalvular atrial fibrillation. N Engl J Med. 2011;365(10):883-91.
34. Hori M, Matsumoto M, Tanahashi N, Momomura S, Uchiyama S, Goto S, et al. Rivaroxaban versus warfarin in Japanese patients with nonvalvular atrial fibrillation in relation to the CHADS2 score: a subgroup analysis of the J-ROCKET AF trial. J Stroke Cerebrovasc Dis. 2014;23(2):379-83.
35. Granger CB, Alexander JH, McMurray JJ, Lopes RD, Hylek EM, Hanna M, et al. Apixaban versus warfarin in patients with atrial fibrillation. N Engl J Med. 2011;365(11):981-92.
36. Lopes RD, Al-Khatib SM, Wallentin L, Yang H, Ansell J, Bahit MC, et al. Efficacy and safety of apixaban compared with warfarin according to patient risk of stroke and of bleeding in atrial fibrillation: a secondary analysis of a randomised controlled trial. Lancet (London, England). 2012;380(9855):1749-58.
37. Giugliano RP, Ruff CT, Braunwald E, Murphy SA, Wiviott SD, Halperin JL, et al. Edoxaban versus warfarin in patients with atrial fibrillation. N Engl J Med. 2013;369(22):2093-104.

38. Mentias A, Shantha G, Chaudhury P, Vaughan Sarrazin MS. Assessment of Outcomes of Treatment With Oral Anticoagulants in Patients With Atrial Fibrillation and Multiple Chronic Conditions: A Comparative Effectiveness Analysis. JAMA network open. 2018;1(5):e182870.
39. Hernandez I, Zhang Y, Saba S. Effectiveness and Safety of Direct Oral Anticoagulants and Warfarin, Stratified by Stroke Risk in Patients With Atrial Fibrillation. The American journal of cardiology. 2018;122(1):69-75.
40. Jaspers Focks J, Brouwer MA, Wojdyla DM, Thomas L, Lopes RD, Washam JB, et al. Polypharmacy and effects of apixaban versus warfarin in patients with atrial fibrillation: post hoc analysis of the ARISTOTLE trial. BMJ (Clinical research ed). 2016;353:i2868.
41. Piccini JP, Hellkamp AS, Washam JB, Becker RC, Breithardt G, Berkowitz SD, et al. Polypharmacy and the Efficacy and Safety of Rivaroxaban Versus Warfarin in the Prevention of Stroke in Patients With Nonvalvular Atrial Fibrillation. Circulation. 2016;133(4):352-60.
42. Kim IS, Kim HJ, Yu HT, Kim TH, Uhm JS, Kim JY, et al. Non-vitamin K antagonist oral anticoagulants with amiodarone, P-glycoprotein inhibitors, or polypharmacy in patients with atrial fibrillation: Systematic review and meta-analysis. Journal of cardiology. 2019;73(6):515-21.
43. Harskamp RE, Teichert M, Lucassen WAM, van Weert H, Lopes RD. Impact of Polypharmacy and P-Glycoprotein- and CYP3A4-Modulating Drugs on Safety and Efficacy of Oral Anticoagulation Therapy in Patients with Atrial Fibrillation. Cardiovascular drugs and therapy. 2019.
44. Martinez BK, Baker WL, Sood NA, Bunz TJ, Meinecke AK, Eriksson D, et al. Influence of Polypharmacy on the Effectiveness and Safety of Rivaroxaban Versus Warfarin in Patients With Nonvalvular Atrial Fibrillation. Pharmacotherapy. 2019;39(2):196-203.
45. Steffel J, Giugliano RP, Braunwald E, Murphy SA, Mercuri M, Choi Y, et al. Edoxaban Versus Warfarin in Atrial Fibrillation Patients at Risk of Falling: ENGAGE AF-TIMI 48 Analysis. Journal of the American College of Cardiology. 2016;68(11):1169-78.
46. Rao MP, Vinereanu D, Wojdyla DM, Alexander JH, Atar D, Hylek EM, et al. Clinical Outcomes and History of Fall in Patients with Atrial Fibrillation Treated with Oral Anticoagulation: Insights From the ARISTOTLE Trial. The American journal of medicine. 2018;131(3):269-75.e2.
47. Martinez BK, Sood NA, Bunz TJ, Coleman CI. Effectiveness and Safety of Apixaban, Dabigatran, and Rivaroxaban Versus Warfarin in Frail Patients With Nonvalvular Atrial Fibrillation. J Am Heart Assoc. 2018;7(8).
48. Subic A, Cermakova P, Religa D, Han S, von Euler M, Kåreholt I, et al. Treatment of Atrial Fibrillation in Patients with Dementia: A Cohort Study from the Swedish Dementia Registry. J Alzheimers Dis. 2018;61(3):1119-28.
49. Orkaby AR, Ozonoff A, Reisman JI, Miller DR, Zhao S, Rose AJ. Continued Use of Warfarin in Veterans with Atrial Fibrillation After Dementia Diagnosis. Journal of the American Geriatrics Society. 2017;65(2):249-56.
50. Fanning L, Lau WCY, Mongkhon P, Man KKC, Bell JS, Ilomäki J, et al. Safety and Effectiveness of Direct Oral Anticoagulants vs Warfarin in People With Atrial Fibrillation and Dementia. Journal of the American Medical Directors Association. 2020:S1525-8610(19)30831-X.
51. The quality assessment tool ‘QUALSYST’ from the “Standard Quality Assessment Criteria for Evaluating Primary Research Papers from a Variety of Fields”. https://www.ihe.ca/advanced-search/standard-quality-assessment-criteria-for-evaluating-primary-research-papers-from-a-variety-of-fields. [Accessed May 4, 2020].
